# Supplementary material for: BCG vaccination to reduce the impact of COVID-19 in healthcare workers: Protocol for a randomised controlled trial (BRACE trial)
Source: BMJ Open. 2021 Oct 27;11(10):e052101. doi: 10.1136/bmjopen-2021-052101 (PMC8557250; doi:10.1136/bmjopen-2021-052101)
Supplement: Supplementary data [file bmjopen-2021-052101supp001.pdf]

Supplementary material

BRACE trial documentation

This was provided as supplementary material to the publication by Pittet LF *et al.*

BCG vaccination to reduce the impact of COVID-19 in healthcare workers: protocol for a randomised controlled trial (BRACE trial)

Contents

BRACE trial protocol 2
BRACE trial code breaking procedure 87
BRACE Data and Safety Monitoring Committee (DSMC) charter 93
BRACE Steering committee composition and description 108
BRACE Leadership and Executive Team (BRACELET) composition and description 110
BRACE authorship guideline 115
BRACE trial Consortium Group composition 117
BRACE Clinicaltrial.gov registration 141
BRACE Participant information and consent form 153
BRACE Participant 3-monthly questionnaire 168

PROTOCOL

RCH HREC/protocol no: 62586

NCT04327206

BCG vaccination to Reduce the impact of COVID-19 in healthcare workers (BRACE) Trial

Version 10.3, 11 February 2021

CONFIDENTIAL

This protocol is confidential and is the property of Murdoch Children's Research Institute. No part of it may be transmitted, reproduced, published, or used without prior written authorisation from the institution.

Statement of Compliance

This clinical trial will be conducted in compliance with all stipulation of this protocol, the conditions of the ethics committees approvals, andthe Integrated Addendum to ICH E6 (R1): Guideline for Good Clinical Practice E6 (R2), dated 9 November 2016.

In Australia, this trial will also be conducted in compliance with with the NHMRC National Statement on ethical Conduct in Human Research (2007 and all updates), the Integrated Addendum to ICH E6 (R1): Guideline for Good Clinical Practice E6 (R2), dated 9 November 2016 annotated with TGA comments and the NHMRC guidance Safety monitoring and reporting in clinical trials involving therapeutic goods (EH59, 2016).

This clinical trial is not sponsored by any pharmaceutical company or other commercial entity.

Pittet LF, *et al. BMJ Open* 2023; 11:e052101. doi: 10.1136/bmjopen-2021-052101

CONTENTS

PROTOCOL SYNOPSIS 6
Primary objectives 6
Secondary objectives 6
GLOSSARY OF ABBREVIATIONS 10
INVESTIGATOR AGREEMENT 11
1. ADMINISTRATIVE INFORMATION 12
1.1. Trial registration 12
1.2. Overall Sponsor 12
1.3. Expected duration of study 12
1.4. Stakeholder involvement 12
2. INTRODUCTION AND BACKGROUND 12
2.1. Trial rationale and aim 12
2.2. Background 13
2.3. Risk/Benefit assessment 15
2.3.1. Known potential risks 15
2.3.2. Known potential benefits 19
2.3.3. Assessment of potential risks and benefits 19
3 TRIAL OBJECTIVES AND OUTCOMES 20
3.1 Objectives 20
3.1.1 Primary objective 20
3.1.2 Secondary objectives 20
3.1.3 Planned exploratory analyses 21
3.2 Outcomes 22
4 TRIAL DESIGN 25
4.1 Overall design 25
4.2 Justification for dose 26
4.3 Trial population 27
4.3.1 Eligibility criteria 27
4.3.2 Inclusion criteria 27
4.3.3 Exclusion criteria 27
4.4 Lifestyle considerations 29
4.5 Screen failures 29
4.6 Recruitment and Consent 29

4.7 Pre-randomisation blood sample 30
4.8 Re-consent 30
5 INTERVENTION 32
5.1 Treatment arms....32
5.2 Trial Intervention(s)....32
5.2.1 Description of trial investigational products 32
5.2.1.1 BCG vaccine SSI 32
5.2.1.2 Placebo to match BCG vaccine SSI 32
5.2.2 Dosage....33
5.2.3 Dose modification 33
5.2.4 Storage and dispensing of BCG vaccine SSI 33
5.2.5 Preparation 33
5.2.6 Administration of trial drug....34
5.2.7 Product accountability 36
5.2.8 Excluded medications and treatments....36
5.2.9 Discontinuation from trial intervention 36
6 RANDOMISATION AND BLINDING 36
6.1 Concealment mechanism....37
7 TRIAL VISITS AND PROCEDURES....38
7.1 Trial timeline 38
7.2 Schedule of assessments....39
7.3 Description of procedures....39
7.4 Notes on specific trial visits....43
7.4.1 Unscheduled visit 43
7.5 Procedure discontinuation, participant withdrawals and losses to follow up 43
7.5.1 Discontinuation of blood collection - participant remains in trial for follow up 43
7.5.2 Withdrawal of consent - participant withdraws from all trial participation....43
7.5.3 Losses to follow-up....43
7.5.4 Replacements....44
7.5.5 Trial Completion....44
7.5.6 Continuation of therapy....44
8 SAFETY MONITORING AND REPORTING....45
8.1 Definitions....45
8.2 Capturing and eliciting adverse event information....46

8.2.1 SAE capture 46
8.2.2 Non-Serious AE Capture 47
8.3 Documentation of AEs....47
8.4 Assessing the relatedness (causality) of a participant's AE 47
8.5 Assessing the severity of a participant's AE 48
8.6 Reporting of safety events 49
9 DATA AND INFORMATION MANAGEMENT 50
9.1 Overview 50
9.2 Data management....50
9.2.1 Data generation (source data) 50
9.2.2 Data capture methods and data use, storage, access and disclosure during the trial 51
9.2.3 Data confidentiality....52
9.2.4 Quality assurance....52
9.2.5 Archiving - Data and document retention....52
9.2.6 Data sharing 53
9.2.7 Long-term custodianship (after archive period finished)....54
9.2.8 Data retrieval and linkage 54
9.2.9 Sample management: Additional data management considerations 54
9.2.10 Sample management: Specimen collection & storage. 55
9.2.11 Sample management: Specimen & Biobanking....55
10 TRIAL OVERSIGHT....56
10.1 Governance structure 56
10.1.1 Trial Steering Committee (TSC)....56
10.1.2 Independent Data and Safety Monitoring Board (DSMB) 56
10.1.3 Independent Safety Monitor 56
10.1.4 Quality Control and Quality Assurance....57
11 STATISTICAL METHODS 57
11.1 Sample Size Estimation 57
11.2 Population to be analysed....58
11.2.1 Handling of missing data 58
11.3 Methods of analysis 58
11.4 Interim Analyses....60
12 ETHICS AND DISSEMINATION....61
12.1 Research Ethics Approval & Local Governance Authorisation 61

12.2 Amendments to the protocol 61
12.3 Protocol Deviations and Serious Breaches 61
13 CONFIDENTIALITY 61
14 PARTICIPANT REIMBURSEMENT 62
15 FINANCIAL DISCLOSURE AND CONFLICTS OF INTEREST 62
16 DISSEMINATION AND TRANSLATION PLAN 62
17 REFERENCES 62
17.1 Appendix 1: Specimens for biobanking - completed biobank registration form 65
17.2 Appendix 2. Collection of stool samples from a subset of BRACE participants 69
17.3 Appendix 3 UK Specific Requirements 73
17.4 Appendix 4 Brazil Specific Requirements 74
17.5 Appendix 5 The Netherlands Specific Requirements 76
17.6 Appendix 6 Spain Specific Requirements 77
17.7 Appendix 7 Optional Biological sample collection during episodes of illness 78
17.8 Appendix 8 Optional Sub-study: collection of blood samples to measure immune responses to COVID-19 specific vaccines. 81

PROTOCOL SYNOPSIS

TITLE	*BCG vaccination to Reduce the impact of COVID-19 in healthcare workers (BRACE) Trial*
TRIAL DESCRIPTION	Phase III, two group, multicentre, randomised placebo controlled trial in up to 7244 healthcare workers to determine if BCG vaccine reduces incidence and the severity of COVID-19 disease during the 2020 SARS-CoV-2 pandemic. The trial includes a pre-planned meta-analysis with data from the 2834 participants recruited in first stage of this study which followed the same protocol but where participants were randomised between BCG and no BCG at the time of receiving a flu vaccination, with a total sample size of 10078.<br><br>Randomisation and immunisation will occur at each participating site. Participants will be randomised to receive BCG vaccine or 0.9% NaCl placebo. Participants will be followed-up for 12 months with notification from a smartphone application (up to daily when ill) or via phone calls, electronic messages, home visits and surveys to identify and detail suspected COVID-19 infection. Additional information on severe disease will be obtained from hospital medical records and/or government databases. Blood samples will be collected prior to randomisation and at 3 and 6, months and in a sub-set of participants at 9 and 12 months to determine SARS-CoV-2 exposure. Where required swab/blood samples will be taken at illness episodes to assess SARS-CoV-2 infection.
OBJECTIVES	**Primary objectives**<br>1. To determine if BCG vaccination (Intervention) compared with placebo (Comparator) <u>reduces the incidence of COVID-19 disease</u> (Outcome) measured over the 6 months following randomisation (Time) in healthcare workers exposed to SARS-CoV-2 (Participants).<br>2. To determine if BCG vaccination (Intervention) compared with placebo (Comparator) <u>reduces the incidence of severe COVID-19 disease</u> (with <u>COVID 19 related</u> non-hospitalised severe disease, hospitalisation or death) (Outcome) measured over the 6 months following randomisation (Time) in healthcare workers exposed to SARS-CoV-2 (Participants).<br><br>**SECONDARY OBJECTIVES**<br>3. To determine if BCG vaccination (Intervention) compared with placebo (Comparator) <u>reduces the incidence of COVID-19 disease</u> (Outcome) measured over the 12 months following randomisation (Time) in healthcare workers exposed to SARS-CoV-2 (Participants).<br>4. To determine if BCG vaccination (Intervention) compared with placebo (Comparator) <u>reduces the incidence of severe COVID-19 disease</u> (non-hospitalised severe disease, hospitalisation or death)

	(Outcome) measured over the 12 months following randomisation (Time) in healthcare workers exposed to SARS-CoV-2 (Participants).<br>5. To determine if BCG vaccination (Intervention) compared with placebo (Comparator) prolongs the time to first SARS-CoV-2-proven respiratory illness (Outcome) measured over 6 and 12 months following randomisation (Time) in healthcare workers exposed to SARS-CoV-2 (Participants).<br>6. To determine if BCG vaccination (Intervention) compared with placebo (Comparator) reduces the severity of COVID-19 disease (Outcome) measured over 6 and 12 months following randomisation (Time) in healthcare workers exposed to SARS-CoV-2 (Participants).<br>7. To determine if BCG vaccination (Intervention) compared with placebo (Comparator) reduces the rate and severity of illness (fever or at least one sign or symptom of respiratory disease) measured over 6 and 12 months following randomisation (Time) in healthcare workers (Participants).<br>8. To determine if BCG vaccination (Intervention) compared with placebo (Comparator) reduces absenteeism (days off work) in healthcare workers (Participants).<br>9. To evaluate the safety of BCG vaccination in adult healthcare workers.<br>**Planned exploratory analyses**<br>10. To determine in a subgroup of adults with recurrent cold sores whether BCG vaccination compared with placebo reduces herpes simplex recurrences (such as cold sores).<br>11. To determine the BCG vaccination induced changes in the immune system that are associated with protection of adult healthcare workers from non-tuberculous infectious diseases including COVID-19.<br>12. To determine and compare changes in the immune system induced by vaccination of adult healthcare workers.<br>13. To identify factors (e.g. age, sex, chronic conditions such as diabetes and cardiovascular disease, smoking, asthma, prior BCG vaccination, genetics, other vaccinations including COVID-19-specific vaccines, latent TB, immunological/molecular factors) that influence adult immune responses, infection and COVID-19 risk.

OUTCOMES AND OUTCOME MEASURES	**Primary outcomes:**<br>1. Number of participants with COVID-19 disease defined as fever or at least one sign or symptom of respiratory disease including cough, sore throat, shortness of breath, respiratory distress/failure (using self-reported questionnaire), plus a positive SARS-Cov-2 test (PCR or serology) over the 6 months following randomisation.<br>2. Number of participants with COVID-19 positive test plus<br>1. Dead (as a consequence of COVID-19 disease)<br>OR<br>2. Hospitalised (including mechanical ventilation and death)<br>OR<br>3. Non-hospitalised severe disease, defined as Non-ambulant[1] for ≥ 3 consecutive days OR Unable to work[2] for ≥ 3 consecutive days<br><br>[1] "pretty much confined to bed (meaning finding it very difficult to do any normal daily activities"<br><br>[2] "I do not feel physically well enough to go to work"<br><br>**Secondary outcomes:** All assessed at 6 and12 months following randomisation unless otherwise indicated.<br>- The following outcomes are for both COVID-19 disease and fever or respiratory illness: Number of participants with: COVID-19 disease, days unable to work, days confined to bed, of days with symptoms, pneumonia, need for oxygen therapy, admission to critical care, need for mechanical ventilation<br>- Number of episodes of COVID-19 disease, fever or respiratory illness<br>- Time to first symptom of COVID-19, fever or respiratory illness<br>- Number of deaths<br>- Number of days of unplanned absenteeism<br>- Type and severity of local and systemic adverse event over the 3 months following randomisation<br>- Planned exploratory analyses : Number of participants with, episodes of and time to first recurrence of herpes simplex recurrence, immunological studies
TRIAL POPULATION	7244 adult healthcare workers from Brazil, Europe and Australia (Victoria, Western Australia, South Australia and New South Wales) will be involved in the study, plus 2834 recruited in the earlier stage of this study. Key exclusion criteria are having BCG vaccine contraindication, previously had a SARS-CoV-2 positive test result and prior involvement in this trial at an alternate study site. Participants will be randomised at 1:1 ratio giving approximately 5039 per group.
DESCRIPTION OF SITES	Multiple sites will enrol healthcare workers in Brazil, Europe and Australia.

ENROLLING PARTICIPANTS	Australian sites involved in this study include the RCH VIC, Monash Health VIC, Epworth Healthcare VIC, Perth Children's Hospital WA, Fiona Stanley Hospital WA, Sir Charles Gairdner Hospital WA, the Royal Adelaide Hospital SA, Women's and Children's Hospital Adelaide SA, The Children's Hospital at Westmead NSW, Westmead Hospital NSW, Prince of Wales Hospital NSW, St Vincent's Hospital NSW and Sydney Children's Hospital, Randwick NSW. Recruitment and follow-up may occur on site or at centrally identified locations overseen by Site Investigators.<br><br>In Brazil, the study will be carried out in two cities, Campo Grande-MS and Rio de Janeiro-RJ. In Campo Grande, the Faculty of Medicine of UFMS, State Regional Hospital of Mato Grosso do Sul, Municipal Health Units, CASSEMS Hospital and Santa Casa Hospital will participate. In Rio de Janeiro the Centro de Referência Professor Hélio Fraga (CRPHF) da Escola Nacional de Saúde Pública Sergio Arouca (ENSP), FIOCRUZ and Municipal Health Office of Rio de Janeiro.<br><br>Additional sites are not yet completely confirmed but will include various sites in Brazil, the Netherlands, Spain and the United Kingdom.
DESCRIPTION OF INTERVENTIONS	BCG vaccination group: BCG Denmark, 0.1 mL injected intradermal over the distal insertion of the deltoid muscle onto the humerus<br>Control group: 0.1 ml of 0.9% NaCl injected intradermal over the distal insertion of the deltoid muscle onto the humerus
TRIAL DURATION	2.5 years
PARTICIPANT DURATION	13.5 months from randomisation to final follow-ups

GLOSSARY OF ABBREVIATIONS

ABBREVIATION	TERM
AE	Adverse Event
AR	Adverse Reaction
BCG	Bacillus Calmette–Guérin vaccine
BRF	Biobank Registration Form (MCRI)
COVID-19	coronavirus disease 19
CRF / eCRF	Case Report Form / electronic Case Report Form
CPI	Chief Principal Investigator
DSMB	Data Safety Monitoring Board
ED	Emergency Department
GCP	Good Clinical Practice
HCW	Health Care Worker/s
HREC	Human Research Ethics Committee
ICH	International Conference on Harmonisation
ITT	Intention To Treat
MERS	Middle East respiratory syndrome
MCRI	Murdoch Children’s Research Institute
NHMRC	National Health and Medical Research Council
NSE	Non-specific effects
NSW	New South Wales
PPE	Personal Protective Equipment
PI	Principal Investigator
QC	Quality Control
RGO	Research Governance Office
RCH	Royal Children’s Hospital (Melbourne)
RPI	Region Principal Investigator
SAE	Serious Adverse Event
SAP	Statistical Analysis Plan
SAR	Serious Adverse Reaction
SARS-CoV-2	Severe Acute Respiratory Syndrome Coronavirus 2
SOP	Standard Operating Procedure
SSI	Significant Safety Issue
SPI	Site Principal Investigator
SUSAR	Suspected Unexpected Serious Adverse Reaction
TB	Tuberculosis
TGA	Therapeutic Goods Administration
UAR	Unexpected Adverse Reaction
USM	Urgent Safety Measure

We use the following terminology with regards to the term 'investigators':

- ***Chief Principal-Investigator** – is used to describe the **overall trial level** Investigator for this multi-site trial: Prof Nigel Curtis of MCRI in Australia (Overall Sponsor)*
- ***Region Principal Investigator** – is used to describe **the region-level Investigator** (i.e. the Region Principal Investigator) responsible for an area including multiple sites in this multi-site trial.*
- ***Site Principal Investigator** – is used to describe **the site-level** Investigator at a participating site in a multi-site trial.*

For some trial sites, one investigator fulfils the role of both Region Principal Investigator and Site Principal Investigator.

INVESTIGATOR AGREEMENT

I have read the protocol entitled "*BCG vaccination to Reduce the impact of COVID-19 in healthcare workers BRACE) Trial*".

By signing this protocol, I agree to conduct the clinical trial, after approval by a Human Research Ethics Committee or Institutional Review Board (as appropriate), in accordance with the protocol, the principles of the Declaration of Helsinki and the good clinical practice guidelines [Integrated Addendum to ICH E6 (R1): Guideline for Good Clinical Practice E6 (R2), dated 9 November 2016].

Changes to the protocol will only be implemented after written approval is received from the applicable Human Research Ethics Committee or Institutional Review Board (as appropriate), with the exception of medical emergencies.

I will ensure that study staff fully understand and follow the protocol and evidence of their training is documented.

Name	Role	Signature and date
Prof Nigel Curtis	Chief Principal Investigator	
Prof Marc Bonten	Region Principal Investigator for The Netherlands and Spain	
Prof Peter Richmond	Region Principal Investigator for Western Australia	
Prof David Lynn	Region Principal Investigator for South Australia	
A/Prof Nicholas Wood	Region Principal Investigator for New South Wales, Australia	
Prof John Campbell	Region Principal Investigator for United Kingdom	
Prof Julio Croda	Region Principal Investigator for Mato Grosso do Sul, Brazil	
Prof Margareth Dalcolmo	Region Principal Investigator for Rio de Janeiro, Brazil	

Pittet LF, *et al. BMJ Open* 2023; 11:e052101. doi: 10.1136/bmjopen-2021-052101

1. ADMINISTRATIVE INFORMATION

1.1. Trial registration

This trial is registered on ClinicalTrials.gov, **NCT04327206.**

1.2. Overall Sponsor

**Trial Sponsor**	MCRI
**Chief Principal Investigator Contact name**	Nigel Curtis
**Address**	Royal Children's Hospital, 50 Flemington Road

On behalf of the Sponsor, MCRI, the Chief Principal Investigator leading the trial will undertake and/or oversee those Sponsor responsibilities delegated by the Sponsor.

1.3. Expected duration of study

The recruitment and IP administration period is expected to take place from March 2020 to March 2021. The individual's follow-up will be 13.5 months from randomisation.

1.4. Stakeholder involvement

Stakeholder
Melbourne Children's Trials Centre (MCTC)
Royal Children's Hospital (RCH)
Hospital directors and staff where participants (staff) will be recruited
Hospitals whose staff will be included as sites
Department of Health (for each state)
Melbourne Academic Centre for Health (MACH)
Royal Children's Hospital Immunisation Service
Australian Health Research Alliance (AHRA)

2. INTRODUCTION AND BACKGROUND

2.1. Trial rationale and aim

In recent months severe acute respiratory syndrome-coronavirus 2 (SARS-CoV-2) has emerged as a novel human pathogen. With no pre-existing immunity against this virus, susceptibly among humans is presumed to be universal. Healthcare workers are at the frontline of novel infectious disease outbreaks such as this. Due to their contact with patients and production of aerosols during some medical procedures they have greater exposure and potentially risk of contracting newly emerged human pathogens. Current strategies to

protect healthcare workers rely on the use (and sustained supply) of personal protective equipment. Healthcare worker absenteeism due to infection with the outbreak pathogen or illness cause by another disease with similar symptoms, compounds the pressure already placed on the healthcare system.

Prophylactic interventions to protect against emerging pathogens are needed, particularly for healthcare workers. The tuberculosis (TB) vaccine, Bacillus Calmette-Guérin (BCG) has beneficial off-target effects and has been shown to protect against non-TB infections[1]. This is proposed to result from BCG mediated boosting of early immune responses. As such, BCG vaccination represents a potential prophylactic intervention to provide protection against emerging pathogens such as SARS-CoV-2.

The aim of this trial is to determine whether in healthcare workers, BCG can reduce the incidence and severity of illness caused by the novel coronavirus, SARS-CoV-2.

2.2. Background

Since the emergence of coronavirus disease 19 (COVID-19) in China in December 2019, there have been over 18,000,000 cases disease and greater than 690,000 deaths caused by the disease globally[2] (as of August 2020). The causative agent of COVID-19 a novel coronavirus, severe acute respiratory syndrome-coronavirus 2 (SARS-CoV-2), has already spread to 108 countries (including over 200 cases in Australia) and it is predicted that up to 60% of the global population could become infected[3]. Following from SARS in 2002[4] and Middle East respiratory syndrome (MERS) in 2012[5], SARS-CoV-2 is the third coronavirus to make the jump from animals to humans and emerge as a serious human pathogen in less than 20 years.

In approximately 80% of cases COVID-19 results in mild to moderate disease with symptoms similar to common respiratory diseases such as influenza-like illnesses, with fever in the majority (87.9%) of cases, followed by dry cough (67.7%), fatigue (38.1%), sputum production (33.4%)[6]. In 14% of cases, SARS-CoV-2 causes severe disease requiring oxygen supplementation and/or mechanical ventilation, with a further 6% being critical cases that have respiratory failure, septic shock and/or organ failure.

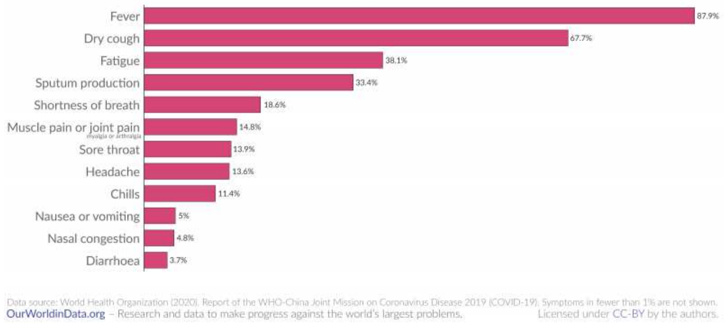

There are worldwide efforts to reduce the peak of SARS-CoV-2 infection, in order to have enough hospital resources. However, with no vaccines or preventative interventions available to protect against COVID-19 disease, current strategies rely on conventional control measures including travel restrictions, quarantines and increased hygiene practices. The overlap of COVID-19 symptoms with common respiratory diseases makes screening for SARS-CoV-2 infection difficult with diagnosis relying on microbiological confirmation of SARS-CoV-2 infection. Moreover, healthcare workers with these common respiratory symptoms are advised to be tested for SARS-CoV-2 infection prior to return to work. The loss of these healthcare workers with non-COVID-19 respiratory infections due to quarantine requirements places further pressure on the healthcare system during this critical time.

BCG, a vaccine given to over 120 million infants annually to protect against TB, represents a potential prophylactic intervention for the prevention of COVID-19 disease. In addition to protecting against TB, BCG has beneficial off target (also termed 'heterologous' or 'non-specific') effects that protect against unrelated infections in children and adults[7-11].

The beneficial off-target effects of BCG vaccination have been most extensively studied in children. A world health organisation (WHO)-commissioned meta-analysis of 12 studies in high mortality settings concluded that BCG vaccination reduces all-cause mortality in children under 5-years of age by 30-53%[8]. This protection is evident within days of vaccination is proposed to be attributable to reduced deaths from infections other than TB, particularly respiratory tract infections and sepsis. Two large cohort studies in children similarly found that BCG reduces non-TB infections. The first, a 25-year retrospective study of over 150,000 children from 33 countries reported that BCG-vaccinated children had an up to 37% lower risk of acute lower respiratory tract infections[12]. The second, a study of paediatric hospitalisations in Spain, found that BCG-vaccinated children had a 41% lower risk of serious respiratory infection and 53% lower risk of sepsis not related to TB[13].

In adults, in a human challenge model, prior BCG vaccination reduced viraemia by over 70% and improved anti-viral immune responses to yellow fever vaccine virus[14]. Notably, yellow fever virus is a single-stranded, positive-sense RNA virus like SARS-CoV-2. Consistent with BCG mediated protection against infections, in two randomised control trials in adults, BCG vaccination reduced incidence of acute upper tract respiratory infections by 70-80%[15,16]. Several studies have also shown that BCG can reduce symptoms in human papilloma virus infection and herpes simplex virus infection adults[17].

A plethora of studies in animal models, have also shown that BCG protects against disease and mortality caused by a wide range of bacterial, fungal, protozoan and viral infections including infections with single-stranded, positive-sense RNA viruses[18-20].

The beneficial off-target effects of BCG are proposed to result from BCG induced changes in immune responses[1,14,19]. In adults, BCG vaccination increases immune responses to unrelated pathogens, an effect that is sustained for at least a year after vaccination[21]. BCG vaccination also boosts antibody responses to several vaccines including influenza vaccine[22-24]. Thus, in addition to protecting against viral infections, BCG provides further protection by increasing the efficacy of other vaccinations.

Therefore, by boosting the immune system, BCG vaccination may provide early protection against new human pathogen thus reducing their spread and severity. This will be of particular benefit among healthcare workers and high-risk groups for whom contraction of the disease would have the greatest impact.

This trial will determine whether BCG vaccination reduces the incidence and severity of COVID-19 but also whether BCG vaccination reduces other respiratory illnesses in healthcare workers. In this case of COVID-19, where symptoms overlap with common respiratory diseases and diagnostic tests currently take several days, the prevention of non-CODVID-19 respiratory illnesses will also reduce the strain on the healthcare system caused by the outbreak. This is particularly important in Australia and other countries in the southern hemisphere as the outbreak peak is expected to occur during the winter influenza season.

The results of this trial will establish whether, in future novel disease outbreaks, BCG vaccination could be implemented as an early intervention to protect healthcare workers and high-risk groups.

2.3. Risk/Benefit assessment

2.3.1. Known potential risks

This study involves minimal risk to participants.

HCWs randomised to receive BCG vaccine will have known potential risks associated with BCG vaccination. These risks are slightly increased for HCWs who have previously had BCG vaccine (revaccination), compared to HCWs receiving BCG vaccine for the first time (vaccine naïve).

There are additional known minimal risks for all HCWs re: blood tests and respiratory swabs.

<u>BCG vaccination</u>

Expected (common) reactions to BCG vaccination[25]:

- A small swelling, redness and tenderness (measuring 0.5-1.5 cm in diameter) at the injection site appears within 1-2 weeks at the injection site. The local lesion evolves into a small ulcer. The ulcer heals over several weeks to months, usually healing into a small flat scar.
- Slightly swollen lymph nodes in the axilla in up to 10% of recipients, and usually resolve spontaneously.

Revaccination is associated with an earlier, accelerated reaction which begins within 24–48 hours of vaccination with induration followed by pustule formation in 5–7 days and healing within 10–15 days[26] (https://www1.health.gov.au/internet/main/publishing.nsf/Content/cda-cdi3701h.htm) ***Tuberculous skin lesions are more common in people over 15 years or with revaccination)***[27,28].

Uncommon side effects of BCG vaccination (up to 1 in 100)[25,29-31]:

- Large ulcer, abscess at the injection site
- Keloid scar at injection site
- Swelling of lymph nodes in the armpit larger than 1 cm across

Rare side effects (up to 1 in 1000)

- Significant inflammation of lymph nodes in the axilla, sometimes with oozing ulcers, possibly abscess
- Infection with the bacteria from the vaccine can occur. The infection can spread throughout the body, including the bones (osteomyelitis)
- Allergic reaction or anaphylaxis (e.g.: redness of the face and neck, swelling of the face, throat or neck, skin rash, breathing difficulties and collapse)
- Fainting, seizures and convulsions (rare among patients receiving injections)

Very rare side effects (1–4 cases per million vaccinated people[25]):

Disseminated BCG infection has been reported rarely after BCG vaccination, mainly in immunocompromised individuals (who are excluded from the trial).

Pittet LF, et al. BMJ Open 2023; 11:e052101. doi: 10.1136/bmjopen-2021-052101

Co-administration of vaccines:

As indicated in the Australian immunisation handbook, BCG vaccine can be given at the same time as, or at any time after, other inactivated vaccines thus there is no additional risk for co-administration of influenza and BCG vaccines[25].

BCG vaccination in Europe (current recommendations) [32]

In Europe, recommendation for BCG vaccination varies among countries. In some, BCG is no longer recommended (e.g. Spain), whereas in others it is given routinely to all neonates (mainly Eastern Europe).[32] In The Netherlands it is limited to the children of parents from countries with a high incidence of tuberculosis (>50/100,000 people) and is not routinely recommended for healthcare workers.[33] In the UK, routine BCG vaccination of adolescents was stopped in 2005, with subsequent efforts focusing on high-risk groups for tuberculosis (UK 'Green Book' chapter 32).

BCG vaccination in Australia (current recommendations)

BCG vaccination in Australia is limited to selected high risk groups and is not routinely recommended for most healthcare workers (HCW)[26]. BCG vaccination is recommended for Aboriginal and Torres Strait Islander neonates in communities with a high incidence of TB; neonates and children 5 years of age and under who will be travelling or living in areas with a high prevalence of TB for extended periods; and neonates born to parents with leprosy. BCG should be considered in HCWs who may be at high risk of exposure to drug resistant cases. It is usually recommended that all individuals have a tuberculin skin test (TST) prior to BCG vaccination, except infants less than 6 months of age with no history of tuberculosis (TB) contact, and that BCG should not be given to an individual with a tuberculin reading of 5mm or more. Additionally, BCG revaccination is not recommended, regardless of TST reaction size[26].

BCG vaccination in Brazil (current recommendations)

In Brazil, BCG vaccination has been mandatory since 1976 in newborns. Revaccination in school-aged children (<6 years) was suspended in 2019. The REVAC trial evaluated adverse reactions resulting from BCG vaccination and revaccination in 71,347 Brazilian school-aged children. The authors concluded that the rate of adverse reactions associated with BCG revaccination is approximately twice the rate associated with vaccination, but this difference was not statistically significant. Similar results have been observed in previous studies that concluded that BCG revaccination is not associated with a higher rate of serious adverse events than primary BCG vaccination.[43,44]

Current contraindications of BCG vaccination

- BCG is contraindicated in immunocompromised individuals due to the risk of disseminated BCG infection[26]. This includes individuals immunocompromised by HIV infection, primary immunodeficiencies, corticosteroids or other immunosuppressive agents, and malignancies involving bone marrow of lymphoid systems.
- BCG is also contraindicated in individuals with any serious illness and those with generalised septic skin diseases and active skin conditions such as eczema, dermatitis and psoriasis near the site of vaccination[25].
- While BCG has not been shown to cause foetal damage the use of live vaccines is contraindicated in pregnancy [26].

Pittet LF, *et al. BMJ Open* 2023; 11:e052101. doi: 10.1136/bmjopen-2021-052101

- Individuals who have previously had tuberculosis or a large tuberculin (TST) reaction

In this study, HCWs will be excluded from the study if they are immunocompromised, have serious illness, skin disease at site of vaccination or are pregnant.

Global BCG recommendations and practices

The current World Health Organization (WHO) position is that BCG revaccination is not recommended for any person, as there is no evidence to support the role of BCG revaccination in protection against tuberculosis[34]. A number of countries have previously included BCG revaccination as part of their national immunisation policies[35]. In 1999, 30 countries in Europe and an additional 18 countries in the Middle East, South East Asia and the Western Pacific region reported using BCG revaccination. In several countries the national policy included BCG in infancy and again at school entry or leaving. In other countries, particularly in Eastern Europe, revaccination with BCG up to age five has been recommended. Some countries, such as Poland, recommended universal revaccination while others restrict revaccination to individuals without a BCG scar or those with a 'negative' TST. Criteria for TST negativity differs between countries [36,37]. ***In countries where BCG revaccination has been part of national immunisation practice, passive surveillance has not reported any particular issues, nor any cases of disseminated BCG in immunocompetent individuals.***

Pre-vaccination screening

TST and interferon gamma release assay (IGRA) screening aims to identify individuals with latent tuberculosis infection (LTBI)[38]. The diameter of induration following TST gives an indication of the likelihood of LTBI, however, positive results can also arise from previous BCG vaccination and exposure to environmental mycobacteria. This is in contrast to IGRA which are unaffected by previous BCG vaccination. A positive IGRA indicates either current or past infection with TB[38]. Screening of individuals using TST prior to BCG vaccination is recommended in Australia and other countries on the grounds that it may prevent complications due to pre-existing immunity due to previous exposure to mycobacterial antigens [28]. ***However, a large review of adverse effects of over 1.5 billion doses of BCG vaccine in adults and children showed that a positive TST did not increase the likelihood of complications from the BCG vaccine and did not predict the development of local skin reactions, abscesses or axillary lymphadenitis[27].***

Trials of BCG revaccination

Three large randomised controlled trials of BCG revaccination in children and adults in Malawi (n=54865), children in Guinea Bissau (n=2871) and adolescents in South Africa (n=990) did not show increased rates of serious adverse events among BCG revaccinated participants[15,39,40]. Participants in the Malawi study did not undergo any pre-randomisation screening with tuberculin skin test (TST) or interferon gamma release assay (IGRA)[39]. This study found a lower rate of leprosy amongst revaccinated participants but no difference in the rates of tuberculosis or death between the groups. Of the children in the Guinea Bissau study, 3 of 6 children with a measurable TST (1-14mm) had increased rates of large local reaction compared to controls (18/388). Two months after revaccination all had healed vaccination scars with no axillary node enlargement, fever or suppurative lymphadenitis[40].

Participants in the South African study all had a negative IGRA at enrolment[15]. Among BCG revaccinated adolescents 93% reported mild local injection site reactions including swelling, induration, discharge, erythema, scab and ulceration. This was compared to 25% in the placebo group. The rates of moderate injection site reactions were similar between the BCG (5%) and placebo (6%) groups. There was 1 severe and 7 serious adverse events in each of the BCG and control groups. The serious adverse events reported in the BCG arm were not attributed to BCG revaccination and included gastroenteritis, chest injury, thermal burn, intentional self-injury, suicide attempt and small intestinal obstruction. The rate of upper respiratory tract infections was also lower in the BCG revaccinated group compared to placebo (2.1% compared to 7.9%, p<0.001).

Further studies looking at BCG revaccination in individuals with positive TST or IGRA do not show increased risk of significant adverse effects. A case-control study of 200 healthy nursing students in India included 28 participants with a positive IGRA who received BCG revaccination[41]. There were no serious side effects reported and no participants developed active tuberculosis during the follow-up study period. A randomised controlled trial of BCG revaccination in healthy adults with a positive TST (>15mm) with or without isoniazid pre-treatment (n=82) showed no difference in the rate of reactions between groups with only local injection site reactions (35-76%) and mild systemic adverse effects (19%) including headache, fever and nausea [42]. Among the 76% of participants who developed ulceration the median ulcer size was 5mm (IQR 4.0-6.0). Maximum ulcer diameter did not correlate with IGRA result prior to BCG vaccination in either group. There were no reports of regional lymphadenitis or serious morbidity.

Enhanced routine passive surveillance of BCG revaccinated school children in the BCG-REVAC trial in Brazil is available for 71718 individuals [43]. There are only 33 reported adverse events of which 60% were local cutaneous reactions and 28% axillary lymphadenopathy without suppuration. There were no deaths, permanent injuries or disseminated infections reported. In a case series of 13 children who experienced adverse events following BCG revaccination in Brazil all developed local ulceration or abscess formation with complete recovery following antimycobacterial therapy[44]. There were no cases of suppurative lymphadenitis or disseminated BCG. Further, an ongoing randomised trial in 150 participants in the US is giving repeat BCG (two vaccinations in the first year, then annually for 4 years) to adults aged 18-65 with type 1 diabetes to test if multiple BCG vaccinations can improve diabetic control and prevent complications[45]. They have reported variable local reactions but no increased risk of lymphadenopathy or disseminated BCG (Denise Faustman, personal communication).

The data presented above supports the WHO position that while BCG revaccination is not recommended due to a lack of evidence of efficacy against tuberculosis the risk of administering BCG vaccine to persons with positive tuberculin reactions due to either prior BCG vaccination or to natural infection is minimal.

One aim of the present study is to document the safety of BCG vaccination (and revaccination) in healthcare workers. The decision not to perform pre-vaccination TST screening in the study is pragmatic in order to reduce barriers to participation for already busy and stretched healthcare workers during the current COVID-19 outbreak. While it does not align with current Australian vaccination guidelines it has been carefully considered upon systematic review of the literature presented above.

Risks related to Placebo injection

Having an injection can sometimes cause very minor pain from the needle or be uncomfortable. The 0.9% NaCl is an inert salt solution that will not cause any degree of local reaction. The placebo injection will be administered by a trained immunization nurse.

Risks related to blood sample collection

Having a blood test can sometimes cause some pain from the needle or be uncomfortable. Occasionally a small amount of bruising can occur on the skin where the blood was taken. Trained members of the study team will collect the blood samples from participants.

Risks related to respiratory swab collection

Having a respiratory swab can sometimes be uncomfortable. Trained members of the study team will collect the respiratory swabs from participants. Self-testing swab kits may be provided as required, with clear instructions to participants on safe self-swabbing technique.

2.3.2. Known potential benefits

In most places in the world, BCG is given to infants and children living in or travelling to TB endemic areas. In adults its efficacy is variable, and likely to have little effect in adults living in low prevalence settings (such as Australia, UK, Spain or the Netherlands) as their risk of TB is very low. BCG also protects against non-TB mycobacterial infections (e.g. leprosy, Buruli ulcer) but these are also rare in Australia and in Europe.

However, BCG also induce beneficial off target effects, and therefore BCG vaccination may reduce COVID-19 illness and other respiratory infections in study participants. In addition to the direct benefit this would give the participants by reducing disease, this would also benefit the healthcare facilities that they work at by reducing their need to be absent (symptom related quarantine or illness) and thus enabling them to continue working and supporting the healthcare system during this period of intense demand.

2.3.3. Assessment of potential risks and benefits

BCG vaccination has a well-established safety profile in healthy individuals. While there are known adverse reactions to BCG, serious adverse reactions are rare. BCG vaccination does also cause a scar in over 80% participants. Participants will be screened prior to BCG vaccination to ensure they have no known contraindications for BCG vaccination. Vaccination will be done by staff trained in intradermal injection to reduce the potential subcutaneous injection which can increase scarring. Blood tests and respiratory swabs will be done by trained staff. If necessary (e.g. insufficient testing capacity or personal protective equipment) participants may be asked to self-collect throat/nose swabs for later collection by study staff.

Given the minor risks of BCG vaccination, the potential benefits of BCG vaccination for the participants (by reducing COVID-19 and other respiratory infections), the healthcare system (by reducing absenteeism) during this current COVID-19 outbreak far outweigh them. In addition to this current outbreak, the findings of this study have major implications for future outbreak responses globally. If BCG vaccination is found to be effective at reducing COVID-19, BCG vaccination could be implemented as an early preventative intervention in future outbreaks to protect healthcare workers globally. BCG vaccine is cheap and already administered to infants in over 80% of countries worldwide, therefore implementation of

BCG vaccination campaigns during outbreaks is a feasible intervention to complement other preventative strategies.

We will be using BCG vaccine outside of its standard/recommended use, therefore, as per use of any intervention outside of standard regulations we will be assessing the reactogenicity and safety of BCG vaccination in vaccine naïve and previously vaccinated healthcare workers.

Risks will be continuously reviewed by continuously checking the literature and communicating with the other research group doing similar BCG trials. We have planned an interim analysis as well within our own cohort.

3 TRIAL OBJECTIVES AND OUTCOMES

3.1 Objectives

Two primary outcomes have been chosen for this study: occurrence of COVID-19 disease and occurrence of severe COVID-19 disease. Considering the number of unknown factors and the little knowledge of this new virus, we deemed it of clinical importance to have sufficient power to detect the potential effect of BCG vaccine compared to control for both outcomes (occurrence of any COVID-19 disease, as well as occurrence of severe COVID-19). Our hypothesis is that, compared to control, the BCG vaccine will reduce both the number of cases of COVID-19 (increase the number of asymptomatic SARS-CoV-2 infections) and the number of severe cases of COVID-19. In other words, we have the hypothesis that BCG vaccine would be able to shift the “severity of COVID-19” curve down, i.e. to generally reduce the severity of the symptoms in healthcare workers. Because of the potential for multiplicity testing, the method of controlling type I error is explained in the sample size section (11.1).

3.1.1 Primary objective

1. To determine if BCG vaccination (Intervention) compared with placebo (Comparator) reduces the incidence of COVID-19 disease (Outcome) measured over the 6 months following randomisation (Time) in healthcare workers exposed to SARS-CoV-2 (Participants).
2. To determine if BCG vaccination (Intervention) compared with placebo (Comparator) reduces the incidence of severe COVID-19 disease (with COVID 19 related death, hospitalisation, or non-hospitalised severe disease (defined as Non-ambulant[1] for ≥ 3 consecutive days OR Unable to work[2] for ≥ 3 consecutive days) (Outcome) measured over the 6 months following randomisation (Time) in healthcare workers exposed to SARS-CoV-2 (Participants).

[1] “pretty much confined to bed (meaning finding it very difficult to do any normal daily activities”

[2]“I do not feel physically well enough to go to work”

3.1.2 Secondary objectives

3. To determine if BCG vaccination (Intervention) compared with placebo (Comparator) reduces the incidence of COVID-19 disease (Outcome) measured over the 12 months following randomisation (Time) in healthcare workers exposed to SARS-CoV-2 (Participants).

Pittet LF, et al. BMJ Open 2023; 11:e052101. doi: 10.1136/bmjopen-2021-052101

4. To determine if BCG vaccination (Intervention) compared with placebo (Comparator) reduces the incidence of severe COVID-19 disease (non-hospitalised severe disease, hospitalisation or death) (Outcome) measured over the 12 months following randomisation (Time) in healthcare workers exposed to SARS-CoV-2 (Participants).
5. To determine if BCG vaccination (Intervention) compared with placebo (Comparator) prolongs the time to first SARS-CoV-2-proven respiratory illness (Outcome) measured over 6 and 12 months following randomisation (Time) in healthcare exposed to SARS-CoV-2 (Participants).
6. To determine if BCG vaccination (Intervention) compared with placebo (Comparator) reduces the severity of COVID-19 disease (Outcome) measured over 6 and 12 months following randomisation (Time) in healthcare workers exposed to SARS-CoV-2 (Participants).
7. To determine if BCG vaccination (Intervention) compared with placebo (Comparator) reduces the rate and severity of illness (fever or at least one sign or symptom of respiratory disease) measured over 6 and 12 months following randomisation (Time) in healthcare workers (Participants).
8. To determine if BCG vaccination (Intervention) compared with placebo (Comparator) reduces absenteeism (days off work) in healthcare workers (Participants).
9. To evaluate the safety of BCG vaccination in adult healthcare workers.

3.1.3 Planned exploratory analyses

10. To determine in a subgroup of adults with recurrent cold sores whether BCG vaccination compared with placebo reduces herpes simplex recurrences (such as cold sores).
11. To determine the BCG vaccination induced changes in the immune system that are associated with protection of adult healthcare workers from non-tuberculous infectious diseases including COVID-19.
12. To determine and compare changes in the immune system induced by vaccination of adult healthcare workers.
13. To identify factors (e.g. age, sex, chronic conditions such as diabetes and cardiovascular disease, smoking, asthma, prior BCG vaccination, genetics, other vaccinations including COVID-19-specific vaccines, latent TB, immunological/molecular factors) that influence adult immune responses, infection and COVID-19 risk.

3.2 Outcomes

OBJECTIVE	OUTCOME & OUTCOME MEASURE
Primary	
1. To determine if BCG vaccination (Intervention) compared with placebo (Comparator) reduces the incidence of COVID-19 disease (Outcome) measured over the 6 months following randomisation (Time) in healthcare workers exposed to SARS-CoV-2 (Participants).	Number of participants with COVID-19 disease defined as<br>Case definition<br>"- positive SARS-CoV-2 test (PCR, antigen or serology), plus<br>- fever (using self-reported questionnaire), OR<br>- at least one sign or symptom of respiratory disease including cough, sore throat, shortness of breath, respiratory distress/failure (using self-reported questionnaire)"<br>over the 6 months following randomisation
2. To determine if BCG vaccination (Intervention) compared with placebo (Comparator) reduces the incidence of severe COVID-19 disease (with COVID related hospitalisation, death, or non-hospitalised severe disease) (Outcome) measured over the 6 months following randomisation (Time) in healthcare workers exposed to SARS-CoV-2 (Participants).	Number or participants with severe COVID-19 disease with COVID related hospitalisation, death, or with non-hospitalised severe disease<br>Case definition<br>Non-ambulant[1] or ≥ 3 consecutive days OR Unable to work[2] for ≥ 3 consecutive days, or death (Outcome) measured over the 6 months following randomisation (Time) in healthcare workers exposed to SARS-CoV-2 (Participants).<br>[1] "pretty much confined to bed (meaning finding it very difficult to do any normal daily activities"<br>[2] "I do not feel physically well enough to go to work"<br>(excludes stay at home exclusively for quarantine/workplace restrictions)
Secondary	
3. To determine if BCG vaccination (Intervention) compared with placebo (Comparator) reduces the incidence of COVID-19 disease (Outcome) measured over the 12 months following randomisation (Time) in healthcare workers exposed to SARS-CoV-2 (Participants).	Number of participants with COVID-19 disease as defined above over the 12 months following randomisation
4. To determine if BCG vaccination (Intervention) compared with placebo (Comparator) reduces the incidence of severe COVID-19 disease (COVID related hospitalisation, death, or non-hospitalised severe disease) (Outcome) measured over 6 and 12 months following randomisation (Time) in healthcare workers exposed to SARS-CoV-2 (Participants).	Number of participants with severe COVID-19 disease as defined above over the 12 months following randomisation
5. To determine if BCG vaccination (Intervention) compared with placebo (Comparator) prolongs the time to first SARS-CoV-2-proven respiratory illness (Outcome) measured over 6 and 12 months following randomisation (Time) in healthcare workers exposed to SARS-CoV-2 (Participants).	Time to first symptom of COVID-19 in a participant who subsequently meets the case definition over the 12 months following randomisation.

6. To determine if BCG vaccination (Intervention) compared with placebo (Comparator) reduces the severity of COVID-19 disease (Outcome) measured over 6 and 12 months following randomisation (Time) in healthcare workers exposed to SARS-CoV-2 (Participants).	All the following measures will be assessed, over the 12 months following randomisation<br><br>Number of participants with COVID-19 disease as defined above<br><br>Number of episodes of COVID-19 disease as defined above<br><br>Number of participants with asymptomatic SARS-CoV-2 infection defined as<br>- Evidence of SARS-CoV-2 infection (by PCR or seroconversion)<br>- Absence of respiratory illness (using self-reported questionnaire)<br>- No evidence of exposure prior to randomisation (inclusion serology negative)<br><br>Number of days unable to work (using self-reported questionnaire) due to COVID-19 disease as defined above (excludes quarantine/workplace restrictions)<br><br>Number of days confined to bed (using self-reported questionnaire) due to COVID-19 disease as defined above<br><br>Number of days with symptoms in any episode of illness that meets the above the case definition for COVID-19 disease<br><br>Number of pneumonia cases (abnormal chest X-ray) (using self-reported questionnaire and/or medical/hospital records) associated with a positive SARS-CoV-2 test<br><br>Need for oxygen therapy (using self-reported questionnaire and/or medical/hospital records) associated with a positive SARS-CoV-2 test<br><br>Number of admission to critical care and duration of stay (using self-reported questionnaire and/or medical/hospital records) associated with a positive SARS-CoV-2 test<br><br>Need of mechanical ventilation and duration (using self-reported questionnaire and/or medical/hospital records) and a positive SARS-CoV-2 test<br><br>Duration of hospitalisation due to COVID-19 (using self-reported questionnaire and/or medical/hospital records)<br><br>Number of deaths (from death registry) associated with a positive SARS-CoV-2 test<br><br>Data will be collected in self-reported participant questionaries, medical/hospital records and/or government registries
7. To determine if BCG vaccination (Intervention) compared with placebo (Comparator) reduces the rate and severity of illness (fever or at least one sign or symptom of respiratory disease) measured over the 12 months following randomisation (Time) in healthcare workers (Participants).	All the following measures will be assessed, over the 12 months following randomisation<br><br>For the following outcomes, fever or respiratory illness will be defined as:<br>- fever (using self-reported questionnaire), or<br>- at least one sign or symptom of respiratory disease including cough, sore throat, shortness of breath, respiratory distress/failure, runny/blocked nose (using self-reported questionnaire)

	Number of participants with fever or respiratory illness, as defined above<br><br>Number of episodes of fever or respiratory illness, as defined above<br><br>Number of days unable to work (using self-reported questionnaire) due to fever or respiratory illness, as defined above (excludes quarantine/workplace restrictions)<br><br>Number of days confined to bed (using self-reported questionnaire) due to fever or respiratory illness, as defined above<br><br>Number of days with symptoms in any episode of illness that meets the above the case definition fever or respiratory illness<br><br>Number of pneumonia cases (abnormal chest X-ray) (using self-reported questionnaire and/or medical/hospital records)<br><br>Need for oxygen therapy (using self-reported questionnaire and/or medical/hospital records)<br><br>Number of admission to critical care (using self-reported questionnaire and/or medical/hospital records)<br><br>Need of mechanical ventilation (using self-reported questionnaire and/or medical/hospital records)<br><br>Number of deaths (from death registry)<br><br>Duration of hospitalisation due to fever or respiratory illness (using self-reported questionnaire and/or medical/hospital records)<br><br>This data will be collected in self-reported participant questionnaires, medical/hospital records and/or government registries
8. To determine if BCG vaccination (Intervention) compared with placebo (Comparator) reduces absenteeism (days off work) in healthcare workers (Participants).	Number of days of unplanned absenteeism for any reason (using self-reported questionnaire) over the 12 months following randomisation
9. To evaluate the safety of BCG vaccination in healthcare workers.	Type and severity of adverse events of interest over the 3 months following randomisation will be collected and graded using toxicity grading scale.<br><br>Serious Adverse Events, over the 3 months following randomisation
Exploratory analyses	
10. To determine in a subgroup of participants with recurrent cold sores whether BCG vaccination compared with placebo reduces herpes simplex recurrence (such as cold sores).	Number of participants with herpes simplex recurrence (self-reported e) over the 12 months following randomisation<br><br>Number of episodes of herpes simplex recurrence (self-reported) over the 12 months following randomisation<br><br>Time: to first of herpes simplex recurrence (self-reported) over the 12 months following randomisation

11. To determine the impact of BCG vaccination on the immune system that are associated with protection of adult healthcare workers from non-tuberculous infectious diseases including COVID-19.<br><br>12. To determine and compare changes in the immune system induced by vaccination of adult healthcare workers.	The immune system will be assessed by several methods including:<br>- Cytokine levels in supernatants from whole blood stimulated with off-target pathogens (including BCG, *Staphylococcus aureus*, *Escherichia coli*) and Toll-like receptor (TLR) agonists, measured by multiplex<br>- Cytokine production, activation and differentiation of immune cells (measured by flow cytometry)<br>- Epigenetic modifications (e.g. histone methylation/acetylation and CpG methylation) measured by ChIP-Seq and/or microarray<br>- Anti-vaccine and anti-pathogen (including SARS-CoV2) antibody levels measured by ELISA, multiplex or VirScan<br>- RNA expression measured by qRT-PCR or RNA-Seq
13. To identify factors (e.g. age, sex, chronic conditions such as diabetes and cardiovascular disease, smoking, asthma, prior BCG vaccination, genetics, other vaccinations including COVID-19-specific vaccines, latent TB, immunological/molecular factors) that influence adult immune responses, infection and COVID-19 responses.	Association of demographic factors, exposure, genetic factors (e.g. single nucleotide polymorphisms) and immune factors (e.g. cell numbers, circulating cytokines, anti-vaccine/anti-pathogen antibodies) with the function of the immune system as described above and COVID-19 prevalence or severity as defined above

4 TRIAL DESIGN

4.1 Overall design

This is a phase III, two group, multicentre, randomised placebo controlled trial in up to 7244 frontline healthcare workers to determine if BCG vaccine reduces prevalence and the severity of COVID-19 disease during the 2020 SARS-CoV-2 pandemic. As part of this study we plan to combine the data from this study in a pre-planned meta-analysis with data from the 2834 participants recruited in the first stage of this study which followed the same protocol but where participants were randomised between BCG and no BCG at the time of receiving a flu vaccination, for a total sample size of 10078. Although we recognise that the first stage of this study was addressing a slightly different research question, we feel that it is important to combine data from the initial stage of study as they both provide estimates of the efficacy of the BCG vaccination, which is critical to provide adequate power to determine the efficacy of the BCG vaccination.

In Europe, healthcare workers from the Netherlands, the UK, Spain and possibly other countries will be recruited across multiple sites.

In Australia, participating sites are hospitals within Victoria, Western Australia, South Australia and New South Wales. Australian sites involved in this study include the RCH VIC, Monash Health VIC, Epworth Healthcare VIC, Perth Children's Hospital WA, Fiona Stanley Hospital WA , Sir Charles Gairdner Hospital WA, Royal Adelaide Hospital SA, Women's and Children's Hospital Adelaide SA, The Children's Hospital at Westmead NSW, Westmead Hospital NSW, Prince of Wales Hospital NSW, St Vincent's Hospital Sydney NSW and Sydney Children's Hospital, Randwick NSW.

In Brazil, there will be a participating sites in Mato Grosso do Sul State and Rio de Janerio. In Mato Grosso do Sol the principal site will the Faculty of Medicine of the Federal University of Mato Gross do Dul (UFMS) with additional locations: Regional Hospital of Mato Grosso do

Sul, Hospital CASSEMS, Hospital Santa Casa and Municipal Health Units. In Rio de Janeiro the principal site will the Centro de Referência Prof Hélio Fraga (CRPFH) da Escola Nacional de Saúde Pública Sérgio Arouca (ENSP), FIOCRUZ and Municipal Health Office of Rio de Janeiro. Other sites in Rio de Janeiro and Mato Grosso do Sul will be identified.

Recruitment may be held at participating sites or centrally identified locations with appropriate safety and privacy infrastructure.

Participants will be randomised to receive BCG or placebo.

During the initial stage of the study in Australia, randomisation and immunisation coincided with the annual staff influenza immunisation roll out at each hospital. During the first stage of the study, influenza vaccine occurred at the same time as randomisation and BCG vaccination or no BCG for 2834 health care workers. During the second stage in locations where the annual influenza vaccine is available, participants are asked to confirm they have received the influenza vaccination a minimum of 72 hours prior to randomisation.

The control group will receive a placebo injection of 0.9% NaCl. Most people vaccinated with the BCG vaccine develop a papule/blister at the injection site around two-weeks after vaccination. Due to this, even using a placebo, it is not possible to completely blind participants to their treatment group allocation. The outcomes (incidence of COVID-19 disease or admission to hospital for COVID-19 disease) are objective measures, it is however still plausible that participant's suspicion of their group allocation might bias the study results. This risk will be mitigated by using a placebo where an element of doubt over treatment allocation may persist even in the absence of scar formation. Members of the research team doing follow-up, data cleaning and analysis will be blinded to the group allocation (by the hiding of this variable and all other variables related to BCG from the dataset) until the formal detailed statistical analysis plan is confirmed and signed by all investigators and all data cleaning/preparation is complete.

Randomisation will be stratified for all factors that might influence the effectiveness of the intervention. For more details see section 6.

Follow-up for all participants will last 1-year. For each episode of fever with a respiratory symptom during the follow-up period, all participants complete a survey in a smartphone app, electronic message or by phone, and may have a home visit by members of the research team for sample collection (e.g. if the government ceases or limits COVID-19 testing; respiratory swab preferred, however blood sample will be taken if no swab testing kits are available). If necessary (e.g. insufficient testing capacity or personal protective equipment) participants may be asked to self-collect throat/nose swabs for later collection by study staff, or self-test a finger-prick blood sample and send a photo of the results to the study team.

4.2 Justification for dose

The dose and route of BCG administration are the standard accepted dosage for BCG vaccine when used to prevent TB. There is no justification to vary from this.

4.3 Trial population

4.3.1 Eligibility criteria

Participants will be assigned to a randomised trial treatment only if they meet all of the inclusion criteria and none of the exclusion criteria.

As soon as COVID 19 specific vaccine becomes available, for sites that are still recruiting participants into the BRACE trial, the site's study team will have to inform the participant before they provide consent, that there will be a delay in receiving their COVID-19-specific-vaccine by either (1) at least 7 days following BCG/placebo injection OR (2) in accordance with their relevant vaccine national guidelines whichever is the longest.

4.3.2 Inclusion criteria

- Over 18 years of age
- Healthcare worker
  - This is defined as anyone who works in a healthcare setting or has face to face contact with patients.
- Provide a signed and dated informed consent form
- Australian sites only: If annual influenza vaccination is available, receiving the flu vaccine is an eligibility requirement. The flu vaccine will be required a minimum of 3 days in advance of randomisation in the BRACE trial.
- Pre-randomisation blood collected

4.3.3 Exclusion criteria

- Has any BCG vaccine contraindication
  - Fever or generalised skin infection (where feasible, randomisation can be delayed until cleared)
  - Weakened resistance toward infections due to a disease in/of the immune system
  - Receiving medical treatment that affects the immune response or other immunosuppressive therapy in the last year.
    - These therapies include systemic corticosteroids (≥20 mg for ≥2 weeks), non-biological immunosuppressant (also known as 'DMARDS'), biological agents (such as monoclonal antibodies against tumour necrosis factor (TNF)-alpha).
  - People with congenital cellular immunodeficiencies, including specific deficiencies of the interferon-gamma pathway
  - People with malignancies involving bone marrow or lymphoid systems

- People with any serious underlying illness (such as malignancy)
  - NB: People with cardiovascular disease, hypertension, diabetes, and/or chronic respiratory disease are eligible if not immunocompromised, and if they meet other eligibility criteria
- Known or suspected HIV infection,[11] even if they are asymptomatic or have normal immune function.
  - This is because of the risk of disseminated BCG infection[12,13]
- People with active skin disease such as eczema, dermatitis or psoriasis at or near the site of vaccination
  - A different adjacent site on the upper arm can be chosen if necessary
- Pregnant
  - Although there is no evidence that BCG vaccination is harmful during pregnancy, it is a contra-indication to BCG vaccination. Therefore, we will exclude women who think they could be pregnant or are planning to become pregnant within the next month.
  - UK specific: Although there is no evidence that BCG vaccination is harmful during pregnancy, it is a contra-indication to BCG vaccination. Therefore, we will exclude women of childbearing potential (WOCBP) who think they could be pregnant. See section 8.2 for definition of WOCBP and Appendix 3 for UK specific pregnancy test requirements.
  - Spain specific: If the patient is female, and of childbearing potential, she must have a negative pregnancy test (provided by Sponsor) at the time of inclusion and practice a reliable method of birth control for 30 days after receiving the BCG vaccination. See Appendix 6 for Spain specific requirements
- Another live vaccine administered in the month prior to randomisation
- Require another live vaccine to be administered within the month following BCG randomisation
  - If the other live vaccine can be given on the same day, this exclusion criteria does not apply
- Known anaphylactic reaction to any of the ingredients present in the BCG vaccine
- Previous active TB disease

- Currently receiving long term (more than 1 month) treatment with isoniazid, rifampicin or quinolone as these antibiotics have activity against *Mycobacterium bovis*

- Previous adverse reaction to BCG vaccine (significant local reaction (abscess) or suppurative lymphadenitis)
- BCG vaccine given within the last year
- Have previously had a SARS-CoV-2 positive test result (positive PCR on a respiratory sample or a positive SARS-CoV-2 diagnostic antigen test approved by the local jurisdiction's public health policy)
- Already part of this trial, recruited at a different site/hospital.
- Participation in another COVID-19 prevention trial
- Have previously received a COVID-19-specific vaccine

4.4 Lifestyle considerations

Not applicable

4.5 Screen failures

Screen failures are defined as participants who consent to participate in the trial but who are found, during the screening procedures, to be ineligible to continue into the trial. They therefore do not receive the intervention / are not randomised.

4.6 Recruitment and Consent

Potential participants will receive information (via email, healthcare facilities notice board and/or website/social media etc) about the trial. This will include a short blurb about the study and a link to a website where they can read further information contact details for further questions. Potential participants will be able to evaluate their eligibility online via the REDCap public link and having met the eligibility criteria access the site specific participant information and consent form (PICF) prior to attending clinic.

Interested healthcare workers will be given the opportunity to talk with a member of the research team by phone or video conferencing if they have any questions (social distancing practices will still be applied wherever possible). The process will vary slightly between locations due to contextual adaptations, however, it will be built around the same core essentials:

- Providing accurate information regarding the trial through a combination of publicly available information and additional detailed explanation by trained study staff
- Eligibility screening for all participants. If participants are ineligible no identifying information will be collected
- Informed consent secured from all participants through signed (electronic or hard copy) PICFs. Consent will be voluntary and free from coercion.
- Study staff will confirm eligibility and consent with prospective participants.

The webpage text, PICFs (electronic or hard copy) and eligibility questionnaire will have prior approval of HREC before use.

In Australia, influenza vaccination 72 hours prior to randomisation is an inclusion criteria as outlined in 4.3.2. For sites outside Australia, the research team will provide recommendation

to all participants not to have the influenza vaccine within the 72 hours prior or post randomisation.

For those who are eligible and provide informed consent, they will be asked to provide their contact details (including date of birth, heathcare card number (or equivalent), name and other identifying details) and a baseline questionnaire on participant characteristic (demographics and environmental information) into either REDCap database or hard-copy forms based on national privacy regulations.

Participants will be told when completing eligibility check (before consenting) that pregnancy, or planning to become pregnant within the next month is a contraindication to getting BCG. We will ask that if they are unsure to do a home pregnancy test, and on the day of randomisation we will have pregnancy tests available that they can use to take away self-test at the site before randomisation. In the UK and Spain completing a pregnancy tests will be eligibility requirement as outlined in Appendix 3 and 6. This will be done in a subtle way to limit the likelihood that other staff will be aware that they have requested a pregnancy test. We have structured it this way to allow people to test in the privacy of their homes rather than have a conversation with the researchers.

Because only 10,078 participants are to be recruited over multiple sites, it is possible that more staff will be interested in participating than can be included in the trial. Given there will likely be interested participants who complete e-consent (where relevant) but are not randomised (become sick, become ineligible, changed their mind) we will continue recruitment until we reach the required number of participants randomised (10,078 participants). Randomisation will cease on the day that 10,078 participants are randomised. On the consent form and other pre-information, interested participants will be informed that due to the limited numbers who can be included in the trial, despite consenting, we cannot guarantee they will be randomised.

Given the importance of finding an intervention that can be used early in future pandemics (before a disease-specific vaccine is available), we expect there will be significant interest from researchers to try and understand how BCG works to boost the immune system. To this end, we will include an optional consent for participants to indicate whether they are interested in being approached for other projects.

No identifying information will be provided to the hospital or recruiting sites regarding any staff who have consented to be part of the trial.

4.7 Pre-randomisation blood sample

To remain eligible for randomisation in BRACE a pre-randomisation bloods sample must be provided. This blood sample will be taken at enrolment but can be taken up to 24 hours prior to randomisation. This sample cannot be taken after administration of the intervention or placebo.

4.8 Re-consent

As required, participants will be contacted through REDCap and sent appropriate and relevant information for re-consent. Re-consent materials will contain contact details for the study team so that participants can ask questions. Participants will be asked if they agree to the changes by signing the re-consent in either electronic or hard copy format depending on

country specific ethics requirements. All participant information for re-consent will be approved by HREC prior to use.

5 INTERVENTION

5.1 Treatment arms

Intervention group: BCG vaccine
Comparator group: 0.9% Saline

5.2 Trial Intervention(s)

5.2.1 Description of trial investigational products

5.2.1.1 BCG vaccine SSI

**Active substance and excipients**	Freeze-dried powder:<br>Live attenuated bacteria of the type *Mycobacterium bovis* BCG (Bacillus Calmette-Guerin), Danish strain 1331<br>0.1 ml vaccine contains between 2 to 8 x 10^5 colony forming units.<br><br>Powder Excipient: Sodium glutamate<br><br>Solvent for resuspension:<br>magnesium sulphate heptahydrate, dipotassium phosphate, citric acid monohydrate, l-asparagine monohydrate, ferric ammonium citrate, glycerol 85%, and water for injections.
**Trade or Generic name**	BCG Vaccine SSI
**Dosage form**	Powder for Injection with solvent for resuspension
**Route of administration**	Intradermal

5.2.1.2 Placebo to match BCG vaccine SSI

**Active substance and excipients**	Sodium Chloride 0.9%.
**Trade or Generic name**	Sodium Chloride Injection BP or USP
**Dosage form**	Ampoule (10 mL)
**Route of administration**	Intradermal

Pittet LF, *et al. BMJ Open* 2023; 11:e052101. doi: 10.1136/bmjopen-2021-052101

5.2.2 Dosage

A single dose of BCG vaccine SSI or matched placebo will be given to all participants who are randomised. The adult dose is 0.1 mL (of BCG vaccine SSI or 0.9% NaCl) injected intradermally over the distal insertion of the deltoid muscle onto the humerus (approximately one third down the upper arm).

5.2.3 Dose modification

There are no allowable dose modifications

5.2.4 Storage and dispensing of BCG vaccine SSI

- Store between 2ºC - 8ºC
- Store in the original package in order to protect from light
- Do not freeze
- Do not use the vaccine after the expiry date which is stated on the carton as "EXP" and refers to the last day of the month listed
- Any unused vaccine at the end of the study, meaning vaccines unused after the last dosing of the last participant will be disposed of according to local regulations

Placebo – sodium chloride 0.9%

- Store less than 25ºC
- Do not use after the expiry date which is stated on the carton and ampoule as "EXP" and refers to the last day of the month listed
- Any unused sodium chloride 0.9% at the end of the study, unused ampoules after the last dosing of the last participant will be disposed of according to local regulations

5.2.5 Preparation

BCG Vaccine SSI

BCG Vaccine SSI consists of a powder and solvent for suspension for injection (2-8 x 10^5 CFU/0.1 mL dose).

Prior to reconstitution, the storage temperature of the BCG will be checked to ensure it the appropriate temperature has been maintained during storage, and transport (if applicable) unless the storage and transport has occurred in validated containers or conditions where the temperature is stable and the data readily available.

The rubber stopper must not be wiped with any antiseptic or detergent. In the eventuality of alcohol being used to swab the rubber stopper, it must be allowed to evaporate before the stopper is penetrated with the syringe needle. The BCG is re-suspended using the solvent provided according to the product directions then carefully inverted a few times to produce uniform resuspension of the lyophilised BCG. Study staff must not shake the vial. The study staff member who re-suspends the BCG will label the vial with the date, time of reconstitution and their initials.

To ensure a uniform suspension, and therefore dose, the vial will be gently swirled before drawing up each dose. When drawn up into the syringe the reconstituted vaccine should appear homogeneous, slightly opaque and colourless.

Each vial of BCG contains up to 10 adult doses. Study staff must NEVER administer the whole vial. Each vial can be kept for up to 4 hours after resuspension. During this time the vial is kept between 2-8°C. Each vial is discarded after 4 hours, or- when the vial is empty, whichever occurs first.

Sodium Chloride 0.9% placebo

During each recruitment session sodium chloride 0.9% will be decanted using aseptic technique into an empty sterile amber glass vial or prepared in 0.1 mL dosing syringes as per local vaccination practices. The study staff member who prepares the sodium chloride for injection will record the date, time of the preparation and their initials.

The prepared sodium chloride for placebo can be kept for up to 24 hours. During this time the placebo is kept between 2-25°C. All prepared syringes or vials unused at the end of a vaccination session will be discarded.

5.2.6 Administration of trial drug

The vaccine or placebo will only be administered by clinician members of the study team trained in the intradermal vaccination technique.

The vaccinator will follow the vaccination SOP and relevant site safety requirements.

Administration of the BCG vaccine or placebo will take place in locations set-up by the study team prioritising participant safety for example ensuring appropriate facilities for management of any potential adverse event are available (e.g anaphylactic reaction, extremely rare). There will be space to allow for privacy for the participant if required (e.g. upper left/right arm not accessible due to clothing).

As per standard practice, participants will be required to remain at the site for 20 minutes after vaccination, in case an allergic reaction should occur, wearing a sticker “I have received the BCG vaccine at [time of vaccination]” for both BCG and placebo recipients.

The time and date of resuspension of the vaccine vial, or placebo preparation, batch identifier, immunisation date/time, any issues with immunisation will be entered in the participants’ study record in REDCap Vaccinators database.

Route/method of administration

The injection site should be clean and dry using non-alcohol based antiseptic. Alcohol antiseptics should not be used prior to administration. If alcohol is used to swab the skin, it must be allowed to evaporate before the vaccine or placebo is injected. The vaccine or placebo must be given strictly intradermally, approximately one third down the upper arm corresponding to the area of the distal insertion of the deltoid muscle, as follows:

- The skin is stretched between thumb and forefinger
- The needle should be almost parallel with the skin surface and slowly inserted (bevel upwards), approximately 2 mm into the superficial layers of the dermis. The needle should be visible through the epidermis during insertion

- The vaccine or placebo should be given slowly

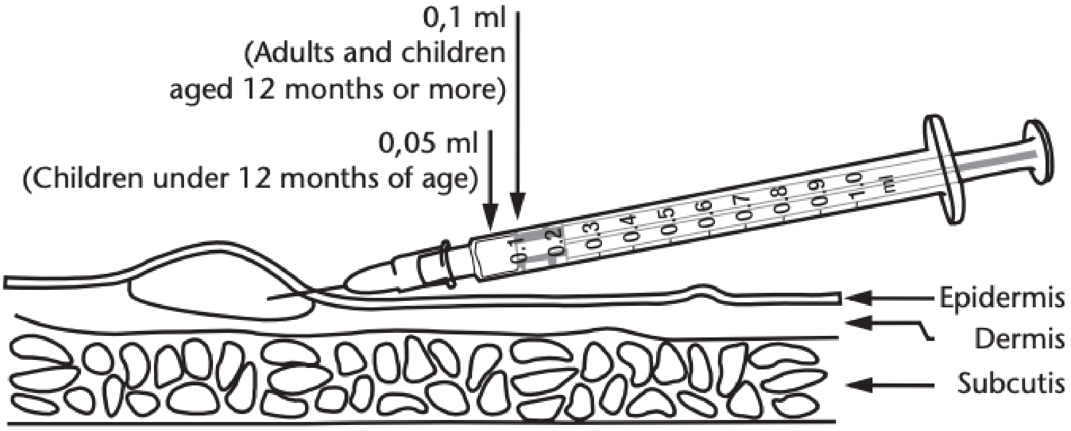

- The mixed vaccine should be administered with a syringe of 1 ml graduated into hundredths of millilitre (1/100) fitted with a short bevel syringe needle (Preference to use 25G or 26G, accepted up to 30G).
- You should feel considerable resistance as you give the injection. If there is no resistance, the needle may be in the subcutaneous tissues.
- If the injection is not intradermal, withdraw the needle and repeat at a new site.
- A raised, blanched papule/bleb of about 7 mm diameter (looks like orange peel) at the needle point is a sign of correct injection
- The injection site is best left uncovered to facilitate healing
- Jet injectors or multiple puncture devices should not be used to administer the vaccine.
- A photo of the bleb (with measuring tape or 10 cent coin used as scale (or equivalent in local currency)) should be uploaded in REDcap form.

Over/under dosage or incorrect administration

Overdose increases the risk of suppurative lymphadenitis and may lead to excessive scar formation. Gross over dosage increases the risk of undesirable BCG complications. Deep injections increase the risk of lymphadenitis and abscess formation.

The clinician members of the research team who administers BCG or placebo as part of this trial will be required to document whether the vaccination was given 'perfectly' with appropriate bleb. Any variations will be documented, and standard procedures followed regarding the need for re-administration, notification to RPI (or delegate).

Complications

All BCG-related complications will be referred to the SPI for advice regarding management. In the very unlikely event a participant has a systemic infection of *Mycobacterium bovis* or persistent local infection following vaccination the SPI will provide advice to the local treating team regarding management, including antibiotic treatment choice. Any serious adverse event or adverse event of interest occurring during the administration of the IP or the 20 minutes post administration will be documented appropriately according to the safety monitoring and reporting section of this protocol.

5.2.7 Product accountability

A pharmacy in each region will act as the study central pharmacy and co-ordinate the storage, distribution and maintain accountability records of the BCG vaccine and placebo supply in that region as appropriate. The RCH pharmacy will act as the study central pharmacy for Australia. The UMC Utrecht pharmacy will be the study central pharmacy in mainland Europe. A UK based pharmacy will act as the central study pharmacy should any UK sites be included in the trial. The LAC/UFMS will act as the central pharmacy in Mato Grosso do Sul, Brazil and a central pharmacy is Rio De Janerio will be identified. Trial accountability of IP including documentation of storage, dispensation and destruction (if required) will be maintained in the pharmacy files at each region/site as appropriate. A pharmacy summary/manual will outline the specific processes for each region in line with local processes and regulations.

Any reason for departure from the expected dispensing regimen will be recorded. At the end of the trial, there will be final reconciliation of trial drug received, dispensed, used and returned. Any discrepancies will be investigated, resolved and documented by the study team.

5.2.8 Excluded medications and treatments

BCG vaccination may be given on the same day of any inactivated or live vaccines. If not given on the same day a period of not less than 4 weeks must pass before giving another live vaccine (although there is no real data supporting this precaution). There must be an interval of at least 3 months before a vaccination in the same arm can take place. Inactivated vaccine (such as the diphtheria-tetanus-pertussis vaccine) can be given in the other arm at any time before, during, or after BCG vaccination if needed.

Participants should not take part in any other COVID-19 preventative intervention clinical trials during the 6 month follow-up period.

5.2.9 Discontinuation from trial intervention

The trial intervention is a once-off vaccination. Due to this there is no possibility to 'discontinue the trial intervention'. If a participant changes their mind between randomisation and vaccination, deciding that they do not want to have the vaccination (but are happy to continue in the study for the follow-up period) they will be included in the analysis as intention to treat.

6 RANDOMISATION AND BLINDING

Once consent has been obtained, and following baseline assessment, eligible participants will be recruited and randomised on the day of the enrolment via Redcap. Randomisation will be to intervention or placebo group with an allocation ratio of 1:1, using a web-based randomisation procedure. The randomisation schedule and web-based service will be provided by an independent statistician from the Clinical Epidemiology and Biostatistics Unit (CEBU) at the Murdoch Children's Research Institute. Randomisation will be in randomly permuted blocks of variable length (2, 4, or 6). Randomisation will be stratified by stage of the study (prior to or post the addition of the placebo vaccination), study site, by age (<40 years; 40 to 59 years; >=60 years) and by presence of comorbidity (any of diabetes, chronic respiratory disease, cardiac condition, hypertension). Stratification by age is necessary for data analysis because

older ages are associated with a greater likelihood of developing severe COVID-19. Likewise, presence of comorbidity is associated with a greater risk of developing severe COVID-19. Each study site will have their own randomisation list stratified by study stage (where relevant), age and presence of comorbidity.

6.1 Concealment mechanism

The control group will receive a placebo of 0.9% NaCl. Most people vaccinated with the BCG vaccine develop a papule/blister at the injection site around two-weeks after vaccination. Due to this, even using a placebo, it is not completely possible to blind participants to their treatment group allocation. The outcomes (incidence of COVID-19 disease or admission to hospital for COVID-19 disease) are objective measures, it is however still plausible that participant's suspicion of their group allocation might bias the study results. This risk will be mitigated by using a placebo where an element of doubt over treatment allocation may persist even in the absence of scar formation. Members of the study team, except immunisers, will be blinded to the group allocation (by the removal of this variable and all other variables related to BCG from the dataset) until the formal detailed statistical analysis plan is confirmed and signed by all investigators and all data cleaning/preparation is complete.

Pittet LF, *et al. BMJ Open* 2023; 11:e052101. doi: 10.1136/bmjopen-2021-052101

7 TRIAL VISITS AND PROCEDURES

7.1 TRIAL TIMELINE

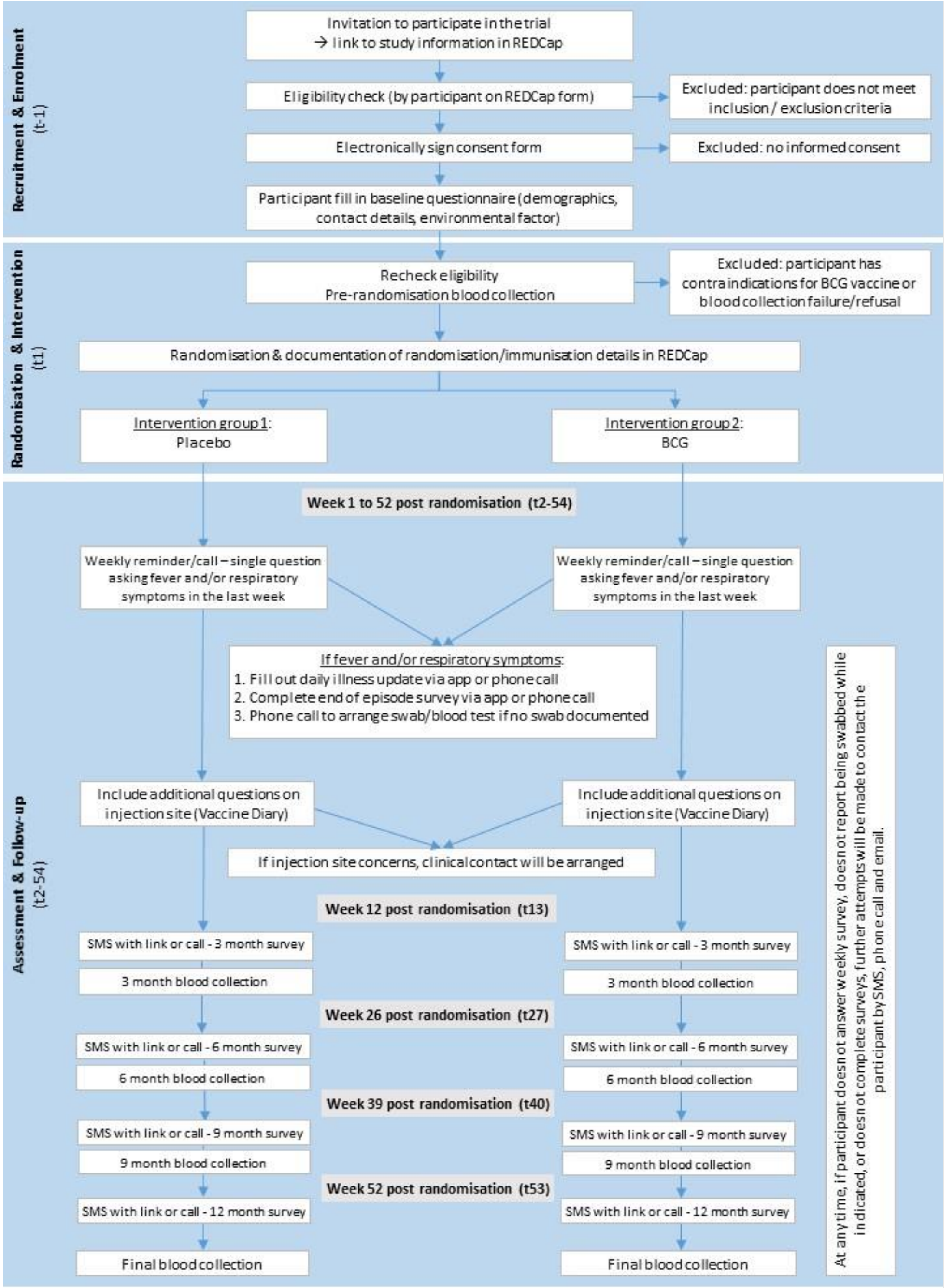

7.2 Schedule of assessments

	TRIAL PERIOD									
	Pre-study	Inclusion & randomisation	Post-randomisation							
TIME POINT	t_{-1}	t_1	t_{2-12}	t_{13}	t_{14-26}	t_{27}	t_{28-39}	t_{40}	t_{41-52}	t_{53}
**RECRUITMENT:**										
Eligibility screen	X									
Informed consent	X									
Contact details	X									
Allocation to intervention		X								
**INTERVENTIONS:**										
BCG vaccine		X (BCG group)								
Saline injection		X (Placebo group)								
**ASSESSMENTS:**										
Baseline questionnaire	X	X								
Weekly survey			X	X	X	X	X	X	X	X
Instruction for swab testing (if indicated by weekly survey)			(X)	(X)	(X)	(X)	(X)	(X)	(X)	(X)
3-month survey				X						
6-month survey						X				
9-month survey								X		
12-month survey										X
*Clinical advice on injection site **			X	X						
*Blood collection***		X		X		X		X#		X#
*Baseline SARS-CoV-2 Test ****		X								

T=week (e.g. t1=first week). A 42day window period is accepted for the periodic survey and the blood collection timepoints)
* In indicated Infectious Diseases clinician, or state-based organisation, as appropriate
** Optional consent for additional biological sample including blood sample when illness reported
*** Brazil only as outlined in Appendix 4
# Sub-set of participants

7.3 Description of procedures

The procedures related to recruitment, consent, eligibility confirmation, randomisation and intervention are described in sections 4 and 5 of this protocol. The procedure for blood collection is described in the relevant SOP. Capture of applicable adverse events is described in section 8.

In Brazil only, a baseline respiratory swab will be collected as outlined in appendix 4.

After randomisation there are two key aspects of the 1-year follow-up period; questionnaires and sample collections for SARS-CoV-2 identification (respiratory swabs or blood samples). Participants will be asked to complete a questionnaire use the smartphone application (app) designed for the trial, electronic messages or via phone calls to report symptoms, access SARS-CoV-2 testing through the public health system and if needed self-collect a respiratory swab each time they have a febrile illness or a respiratory symptom,. Where app is utilised, participants will be trained on how to use the app on day of enrolment.

Questionnaires

Baseline

- Comorbidities: diabetes, cardiovascular disease, chronic respiratory disease, hypertension
- Risk factors: smoking, body mass index (calculated with weight and height)
- BCG/TB history: Prior BCG vaccination, ever positive TST
- Influenza immunisation: date of last influenza vaccine (if within influenza season)
- Exposure (presence of COVID-19 cases at workplace, average days working in hospital or other healthcare settings as appropriate)
- Other: Recurrent herpes infection (such as cold sores)

Regular (generally weekly) questionnaires on smartphone app, via phone call or via electronic messages

- Any symptoms of COVID-19: fever or at least one sign or symptom of respiratory disease such as sore throat, cough, shortness of breath, respiratory distress/failure (y/n)

For each episode of illness (via smartphone app, via phone call or via electronic messages)

- Which symptoms of COVID-19: fever, cough, shortness of breath and/or difficulty breathing, runny/blocked nose, sore throat, fatigue, muscle and/or joint ache, headache, nausea, vomiting and/or diarrhoea, loss of taste and smell
- Has a COVID-19 swab been taken? (if so what was the result)
- Date of/days since onset and cessation of symptoms
- Days absent from work (total number and number due to illness)
- ED presentations
- Hospital admission (oxygen, ICU admission, mechanical ventilation)
- Known test results
- If a swab has been taken for clinical purposes, who ordered it
- Impact on daily activities
- Days in bed
- Chest x-ray results

For local reaction to injection: the Vaccine Diary (daily diary for the two weeks following randomisation)

- Questionnaire collecting common reactions to the injection, including photograph of injection site

Periodic questionnaires (once every 3 months)

- Exposure (presence of COVID-19 cases at workplace, average days working in hospital or other healthcare settings as appropriate)
- Cold Sore recurrence
- Request for participants to confirm the main episodes of illness experienced in the prior 3 months.

- Screening, exposure or treatment of TB
- Detail on other vaccinations
- Hospitalisation (any)
- Information regarding participation in any other COVID-19 preventative intervention clinical trials
- Injection site evolution and side effects (photo of injection site)
- Treatment that could influence COVID-19 outcome

Additional questions for 3rd month questionnaire only:

- Record any non-serious adverse event of interest, including Injection site evolution and side effects (photo of injection site), with onset between randomisation and 3 months post randomisation
- If relevant: Influenza vaccine side effects
- Record any serious adverse event with onset between randomisation and 3 months post randomisation

Swabs

- Where a participant has had a swab sample assessed outside the indication of the study (e.g. with non-respiratory symptoms or asymptomatic) results will be collected via self-report in the 3 monthly questionnaires. All test results will also be obtain, where possible from centralised SARS-CoV-2 testing government database.

Where a participant has symptoms of febrile or respiratory illness (cough, sore throat, shortness of breath) and a swab sample for SARS-CoV-2 is not collected through standard pathways (for example due to swab shortage, or government decision to restrict screening to high-risk patients), a sample collection study visit may be done. A respiratory swab/s may be collected from the participant at home and linked with the relevant public health testing and reporting systems. If respiratory swab/s are done by participant self-collection (e.g. nasal/throat swabs) they will receive full instructions on how to take the samples, when to take them and how to correctly store them until a member of the research team collects them.

Blood samples

- At randomisation, a blood sample will be taken for later assessment of seroconversion (production of specific anti-SARS-CoV-2 antibodies). This will identify participants who had SARS-CoV-2 exposure and immunity prior to commencement of the study.
  - The baseline blood samples will be analysed in batch months after randomisation, so there will be no clinically actionable results. We will provide individualised results to participants via email after completion of the trial. This email will be sent to the applicable HRECs to review before being sent out to any participant.
  - In Brazil, IGRA testing will be completed on pre-randomisation blood samples as outlined in appendix 4.
- At 3 months and 6 months (+ 42 days) post randomisation, the study team will coordinate to collect study blood samples from participants and in a sub-set of

participants at 9 and 12 months (+ 42 days) post randomisation. This will identify participants who had an immune response to SARS-CoV-2 (surrogate marker of infection) during the study. This is needed to determine asymptomatic SARS-CoV-2 infections.
- Blood samples will also be taken for assessment of the immune system. This is will be used to meet the planned exploratory analyses related to vaccine induced changes in the immune system. These blood samples will be taken at the same time as blood collection for serum or plasma samples (i.e. at randomisation and post randomisation)
- In the eventuality that it is unfeasible to collect swab samples to confirm SARS-CoV-2 infection at the time of febrile or respiratory illness episodes (or conduct a validated antigen test), seroconversion may be used to associate episodes of febrile or respiratory illness with SARS-CoV-2 infection. Therefore, for episodes of febrile or respiratory illness where a swab sample cannot be taken, 1 month after the onset of symptoms (expected peak post-infection antibody production), participants may be asked to come to the hospital to provide a blood sample. If rapid point-of care testing is available, these tests may be distributed to participants to self-test. Should these alternative methods of testing become required, an amendment will be submitted to HRECs to outline the process and submit any information for participants. This testing will not be conducted without further consultation with and approval from the HRECs, including providing the HRECs with details of the test and its efficacy.

For blood sample collection for serum/plasma plus analysis of the immune system, a venous blood sample (up to 10ml or up to 35ml depending on the study site) will be taken by a trained member of the study team and labelled with participant ID, date/time collected, study timepoint, year of birth (no identifying information). Samples will be transported to the site's designated laboratory for the trial. Samples will be processed for serum/plasma separation and analysis of the immune system and stored at -80˚C or in liquid nitrogen for later assessment.

A self-collected dried blood spot may be requested from participants instead of a venous blood sample collection. These may be stored in a locked cabinet prior to elution and storage at -80˚C. If blood samples are done by participant self-collection of dried blood spot, participants will receive full instructions on how to take the samples, when to take them and how to correctly store them until they are returned to the study site.

<u>Data Retrieval</u>
Data retrieval and linkage is further described in Section 9 of this Protocol.

The present study expects that it will acquire some research data from existing administrative and service data sources. In Victoria, for example, this would include obtaining details from the Victorian Department of Health and Human Services (VDHHS) who collects information about presentations to hospitals and emergency departments for medical care in Victoria. Similar processes will be followed in other Australian states. In mainland Europe and Brazil, participants will be required to consent to provide access to their medical records by study staff. In the UK self –reports from participants may be supplemented by tracking of participants using their NHS number or other relevant unique identifiers (provided by

participant), drawing on Hospital Episode Statistics and Office of National Statistics data to track health service use (admissions) and deaths.

7.4 Notes on specific trial visits

7.4.1 Unscheduled visit

If participants have any concerns related to side effects or the injection site evolution or scaring, they can call or email the study team for advice and if necessary, they will be seen by a clinician member of the study team or delegate. Reassurance, appropriate management or referral for medical care will be done according to best practice. Documentation of adverse event will be done as indicated in section 8.

7.5 Procedure discontinuation, participant withdrawals and losses to follow up

7.5.1 Discontinuation of blood collection - participant remains in trial for follow up

The Participants that decline further blood collection may still continue in all other aspects of the study.

7.5.2 Withdrawal of consent - participant withdraws from all trial participation

Participants are free to withdraw from the trial at any time upon their request. Withdrawing from the trial will not affect their access to standard treatment or their employment as their participation will not be shared with their employer.

For the safety of all participants withdrawing from the trial, reasonable efforts should be made to undertake protocol-specified safety evaluations.

A dedicated Case Report Form (CRF) page will be used to capture the date of participant withdrawal of consent, and the reason if offered.

7.5.3 Losses to follow-up

Due to the study taking place in healthcare workers during a pandemic, we expect that there may be periods that participants will ignore the smartphone app prompts, phone calls or electronic messages. This includes the eventuality that a participant has been admitted to hospital. The weekly smartphone app prompts will only ask whether the participant has had a fever or respiratory symptom since the last time they answered in the app (date provided). Alternatively where appropriate phone follow-up will be used (ie. Brazil). We deem this very unlikely to annoy participants excessively as they can ignore the notification or call if they are too busy (or withdraw). This will give the project the best chance of having a complete dataset to analyse as they can answer ‘Yes’ when they get the opportunity and fill in the associated questionnaire. Therefore, we will continue to send out weekly notifications or calls for the entire study regardless of whether they respond.

In Australia and Europe, if a participant does not answer 2 regular smartphone app prompts (2 consecutive weeks), further attempts will be made to contact them by electronic messages (maximal 3 attempts), phone call (maximal 3 attempts) and email (maximal 3 attempts). If there is still no response, and the participant is not found to have died on medical records, we will try to contact them later (when the workload is expected to have decreased).

Supplemental material BMJ Publishing Group Limited (BMJ) disclaims all liability and responsibility arising from any reliance placed on this supplemental material which has been supplied by the author(s) BMJ Open

In Brazil, if a participant does not answer 3 follow-ups phone contacts (phone call or electronic messages), a home visit may be carried out by study staff. If there is still no response, and the participant is not found to have died on medical records, we will try to contact them later (when the workload is expected to have decreased).

Where secondary contact provided, the study team will follow-up if unable to contact participants.

7.5.4 Replacements

Participants who have been randomised may NOT be replaced.

7.5.5 Trial Completion

A participant is considered to have completed the trial if he or she has completed all processes of the trial including the last visit or the last scheduled procedure shown in the Schedule of Assessments.

The end of the trial is defined as completion of the last visit or procedure shown in the Schedule of Assessments in the trial at all sites. At the end of the trial, the Sponsor-Investigator will ensure that all HRECs as well as all regulatory and funding bodies have been notified, if required.

This trial may be temporarily suspended or prematurely terminated if there is sufficient reasonable cause. If the trial is prematurely terminated or suspended, the Sponsor and Investigators will promptly inform trial participants, HRECs, the funding (where applicable) and regulatory bodies, providing the reason(s) for the termination or suspension.

Circumstances that may warrant termination or suspension include, but are not limited to:

- Determination of an unexpected, significant, or unacceptable risk to participants that meets the definition of a Significant Safety Issue (for the definition refer to Section 8.1).
- Insufficient compliance to protocol requirements
- Data that are not sufficiently complete and/or evaluable
- Demonstration of efficacy that would warrant stopping
- Determination that the primary endpoint has been met
- Determination of futility

In the case of concerns about safety, protocol compliance or data quality, the trial may resume once the concerns have been addressed to the satisfaction of the sponsor, HRECs, funding and/or regulatory bodies.

7.5.6 Continuation of therapy

As the treatment is ‘once-off’ there is no provision for continuation of therapy.

Pittet LF, *et al. BMJ Open* 2023; 11:e052101. doi: 10.1136/bmjopen-2021-052101

8 SAFETY MONITORING AND REPORTING

8.1 Definitions

Adverse Event (AE):

An AE is any untoward medical occurrence in a participant administered an investigational product and does not necessarily have a causal relationship with the study treatment. For this study, only certain adverse events are recorded, specifically serious adverse events as defined below, and non-serious adverse events of interest specified in section 8.2

Serious Adverse Event (SAE) :

Any serious adverse event (SAE) is an untoward medical occurrence that:

- Results in death; or
- Is life-threatening; or
- Requires hospitalisation or prolongation of existing hospitalisation;
  - Hospitalisation is to be considered an SAE only in the event of an overnight admission. Any elective hospitalisation does not constitute an SAE
- Results in persistent or significant disability/incapacity; or
- Is a congenital anomaly/birth defect

Note: Life-threatening refers to an event in which the participant was at risk of death at the time of the event. It does not refer to an event that hypothetically might have caused death if it were more severe.

Medical and scientific judgement should be exercised in deciding whether an adverse event should be classified as serious in other situations. **Important medical events** that are not immediately life-threatening or do not result in death or hospitalisation but may jeopardise the participant or may require intervention to prevent one of the other outcomes listed in this definition should also be considered serious.

For this study, all SAE will be collected for the period from randomisation to 3 months post randomisation.

Suspected Unexpected Serious Adverse Reaction (SUSAR):

A SUSAR is an AE that meets all of the following criteria:

- The AE is serious (as defined above; an SAE); and
- The SAE is suspected adverse reaction to the investigational product, meaning it is judged by either the reporting investigator or the sponsor as having a reasonable possibility of a causal relationship to a study vaccine (possibly, probably or definitely related), and
- The SAE is also unexpected: An unexpected serious adverse reaction is one for which the nature or severity of the reaction is not consistent with reference safety information (Which is comprised of the BCG vaccine Product Information and the *WHO information sheet: Observed rate of vaccine reactions Bacille Calmette Guerin Vaccine April 2012*).

Note that an event is instead considered 'expected' if it is listed in the Reference Safety Information and therefore cannot meet the definition of SUSAR.

Significant Safety Issue:
A significant safety issue is an issue that could adversely affect the safety of participants or materially impact on the continued ethical acceptability or conduct of the trial.
Comment: A significant safety issue is a new safety issue or validated signal considered by the Sponsor in relation to the study vaccines that requires urgent attention of stakeholders. This may be because of the seriousness and potential impact on the benefit-risk balance of the study vaccines, which could prompt regulatory action and/or changes to the overall conduct of the clinical trial, including the monitoring of safety and/or the administration of the study vaccines.

Urgent Safety Measure (USM):
A measure required to be taken in order to eliminate an immediate hazard to a participant's health or safety. Note: This is a type of significant safety issue that can be instigated by either the investigator or sponsor and can be implemented before seeking approval from HRECs or institutions.

8.2 Capturing and eliciting adverse event information

For the period of randomisation to 3 months post randomisation only, the following are non-serious adverse events of interest for this study:

- At injection site:
  - Reaction (pain, tenderness, redness, swelling) of grade 3 (severe) or 4 (potentially life threatening)
  - Abscess
  - Large ulcer (>1.5 cm diameter)
  - Keloid scar
  - Unusual local reaction
- Lymphadenopathy (in region of injection site)
- BCG osteitis/osteomyelitis
- Disseminated BCG infection (BCG-osis)
- Allergic reaction due to IP
- Fainting episode, seizures and convulsions following IP administration (recorded on the day of IP administration only)

Only these non-serious AE and all SAE, occurring between randomisation and 3 months post randomisation, will be recorded for this study. If applicable, for the remainder of the follow-up period, sites may additionally document participants' AE as required to meet reporting requirements of the applicable HREC/s and/or regulatory authority.

8.2.1 SAE capture

SAE are captured on the day of IP administration, as recorded by the site personnel. Information on any SAE since randomisation will be solicited from participants at the 3-month questionnaire. SAE may also be captured via participant notification, in the period

between randomisation and the 3-month questionnaire, such as through spontaneous contact by the participant via call or email, data entered in the Vaccine Diary or the study smartphone app (or equivalent, e.g. weekly phone calls). In cases where a participant does not respond to multiple attempts at contact, over several weeks, the participant's secondary contact will be contacted to confirm their status and record fatal SAE if applicable.

For this study, all SAE will be collected for the period from randomisation to 3 months post randomisation.

8.2.2 Non-Serious AE Capture

Non-serious AE of interest are captured:

- On the day of IP administration, recorded by the site personnel
- Within the Vaccine diary (which triggers an alert to the site personnel to contact the participant)
- Through the 3-month questionnaire (questions on injection site evolution)
- Through spontaneous contact (e.g. phone call, electronic message or email) from the participant to the site team.

8.3 Documentation of AEs

For the purposes of this study the investigator or delegate is responsible for recording the applicable Adverse Events, regardless of their relationship to study vaccines.

The documentation of each applicable AE on the REDCap CRF will include:

- A description of the AE
- The onset date, duration, date of resolution
- Severity
- Seriousness (SAE or not)
- Any action taken (e.g. treatment, follow-up tests)
- The outcome (recovery, death, continuing)
- The likelihood of the relationship of the AE to the trial treatment

All AEs will be followed to resolution or stabilisation, where possible.

8.4 Assessing the relatedness (causality) of a participant's AE

All non-serious AE of interest and SAE must have their relationship to the trial intervention assessed by the SPI (or delegate) who evaluates the AE based on temporal relationship and their clinical judgment. The degree of certainty about causality will be graded using the categories below.

The relationship of the event to the trial intervention will be assessed as follows:

Code	Causal Relationship	Description
1	Unrelated	The AE is clearly NOT related to the intervention
2	Unlikely	The AE is doubtfully related to the intervention
3	Possible	The AE may be related to the intervention
4	Probable	The AE is likely related to the intervention
5	Definite	The AE is clearly related to the intervention

8.5 Assessing the severity of a participant's AE

The SPI (or delegate) will be responsible for assessing the severity of an AE. The determination of severity for all AE should be made by the investigator based upon medical judgment and the severity categories of Grade 1 to 5 as defined in the first table below, with the following exceptions: injection site pain, redness, tenderness and swelling/induration are assigned severity grades using the specific toxicity grade specified in the second table below.

Grade	Severity	Description
Grade 1	Mild	Asymptomatic or mild symptoms; clinical or diagnostic observations only; intervention not indicated
Grade 2	Moderate	Moderate; minimal, local or non-invasive intervention indicated; limiting age appropriate instrumental activities of daily living (ADL)
Grade 3	Severe	Severe or medically significant but not immediately life-threatening; hospitalisation or prolongation of hospitalisation indicated; disabling; limiting self-care ADL
Grade 4	Life Threatening	Life-threatening consequences; urgent intervention indicated
Grade 5	Fatal	Death related to AE

Pittet LF, *et al. BMJ Open* 2023; 11:e052101. doi: 10.1136/bmjopen-2021-052101

Toxicity grading scale

Local reaction to vaccination are monitored using Vaccine diary completed by the participant up to 14 days after vaccination. A toxicity grading scale is used to categorise the reports (Food and Drug Administration 2007):

**Local reaction**	**Grade 0** None	**Grade 1** Mild	**Grade 2** Moderate	**Grade 3** Severe	**Grade 4** Potentially life threatening
**Pain**	None	Does not interfere with activity	Repeated use of nonnarcotic pain reliever > 24 hours or interferes with activity	Any use of narcotic pain reliever or prevents daily activity	Emergency room visit or hospitalization
**Redness**	None	2.5 - 5 cm	5.1 - 10 cm	>10 cm	Necrosis or exfoliative dermatitis
**Tenderness**	None	Mild discomfort to touch	Discomfort with movement	Significant discomfort at rest	Emergency room visit or hospitalization
**Swelling / induration**	None	2.5 - 5 cm and does not interfere with activity	5.1 - 10 cm or interferes with activity	>10 cm or prevents daily activity	Necrosis
**Itch**	None	Itching localised to injection site that is relieved spontaneously or in <48 hours of treatment	Itching beyond the injection site that is not generalised OR Itching localised to injection site requiring ≥48 hours of treatment	Generalised itching causing inability to perform usual social & functional activities	Not applicable

Food and Drug Administration. (2007). "Guidance for Industry: toxicity grading scale for healthy adult and adolescent volunteers enrolled in preventive vaccine clinical"
Retrieved 08.04.2020, from https://www.fda.gov/downloads/BiologicsBloodVaccines/GuidanceComplianceRegulatoryInformation/Guidances/Vaccines/ucm091977.pdf.

8.6 Reporting of safety events

Site Principal Investigator Reporting Procedures:

The SPI (or delegate) is responsible for expedited reporting (within 24 hours of becoming aware of the event) to the Sponsor the following:

- USMs
- All SAEs (including SUSAR)

SAE reports should be submitted using the RedCap SAE form, or by alternative means specified in the Safety Reporting Plan.

At MCRI, the CPI (or delegate) will determine whether or not each SAE meets the definition of SUSAR and will notify all RPI in a timely manner.

The RPI and SPI will be notified of USM and other significant safety issues in a timely manner following MCRI first knowledge of the event/s.

In each country, USM, other significant safety issues, SUSARs and other SAE, will be reported to the applicable regulatory authorities and HRECs in accordance with the requirements.

Further details of event reporting responsibilities and processes are documented in the Safety Reporting Plan.
For safety reporting requirements specific to Brazil, refer to Appendix 4.

9 DATA AND INFORMATION MANAGEMENT

9.1 Overview

The Site Principal Investigator is responsible for storing essential trial documents relevant to data management and maintaining a site-specific record of the location(s) of the site's data management-related Essential Documents.

The Site Principal Investigator is responsible for maintaining adequate and accurate files of any relevant source documents that include observations or other data relating to participants at their site. Source data will be attributable, legible (including any changes or corrections), contemporaneous, original, accurate, complete, consistent, enduring and available. Changes to source data (hardcopy and electronic) must be traceable, must not obscure the original entry, and must be explained where this is necessary.

The Site Principal Investigator will also maintain accurate case report forms (CRFs) (i.e. the data collection forms)where applicable and be responsible for ensuring that the collected and reported data is accurate, legible, complete, entered in a timely manner and enduring. To maintain the integrity of the data, any changes to data (hardcopy and electronic) must be traceable, must not obscure the original entry, and must be explained where this is necessary.

Any person delegated to collect data, perform data entry or sign for data completeness will be recorded on the delegation log and will be trained to perform these trial-related duties and functions.

9.2 Data management

9.2.1 Data generation (source data)

In this study, the following types of data will be collected:

- personal identifying information (names, dates of birth, contact details; NHS number in UK, Medicare number in Australia, SUS CARD and CPF in Brazil)
- sensitive information including health data (medical history, participant eligibility, adverse reactions and other notes as appropriate)
- participant completed electronic questionnaires
- de-identified data from laboratory assays

Source document plan

Much of the data for this trial will be collected electronically directly from participants. There will be a limited number of source documents for this study recorded data from automated instruments, laboratory reports and the signed information and consent forms (in REDCap or hard copy). Each site participating in the trial will maintain a site-specific Source Document Plan that will document the source, i.e. original recording, for each data discrete item/ category of items collected for the study. This Source Document Plan, signed and dated by

the Site Principal Investigator, will be prepared prior to recruitment of the first participant and will be filed in the site's Investigator Site File.

9.2.2 Data capture methods and data use, storage, access and disclosure during the trial

Data collection methods

Data for this trial will be collected and entered using electronic database REDCap and a smartphone application developed for this trial. REDCap is a secure, web-based application for building and managing online surveys and databases. The trial smartphone application stores participant information directly in the REDCap database. In line with local privacy regulations, identifying or personal data may be maintained in complementary site level information management systems as required.

Use of the data

The data will be used for the analyses specified in the protocol and Statistical Analysis Plan.

Following the completion and analysis of the trial, the data will be retained long-term following the mandatory archive period for use in future research projects.

Storage and access

Hard copy data will be stored by collaborators in a locked cabinet in a secure location, accessible to the research team only.

Electronic data maintained on REDCap database will be securely stored in MCRI's ’network file servers, which are backed up nightly. Electronic or hard copy files containing private or confidential data will be stored only in locations accessible only by appropriate designated members of the research team.

REDCap is hosted on MCRI infrastructure and is subject to the same security and backup regimen as other systems (e.g. the network file servers). Data is backed up nightly to a local backup server, with a monthly backup taken to tape and stored offsite. REDCap maintains an audit trail of data create/update/delete events that is accessible to project users who are granted permission to view it. Access to REDCap will be provided via an MCRI user account or (for external collaborators) via a REDCap user account created by the MCRI system administrator. The permissions granted to each user within each REDCap project will be controlled by, and will be the responsibility of, the study team delegated this task by the Principal Investigator. REDCap has functionality that makes adding and removing users and managing user permissions straightforward. All data transmissions between users and the REDCap server are encrypted. The instructions for data entry to REDCap must be read and the training log signed prior to personnel commencing data entry on REDCap.

Authorised representatives of the sponsoring institution as well as representatives from the HREC, Research Governance Office and regulatory agencies may inspect all documents and records required to be maintained by the CPI for the participants in this trial. The trial site will permit access to such records.

Disclosure

The trial protocol, documentation, data and all other information generated will be held in

strict confidence. No information concerning the study or the data will be released to any unauthorised third party, without prior written approval of MCRI. Clinical information will not be released without written permission of the participant, except as necessary for monitoring by the HREC, Research Governance Office or regulatory agencies.

9.2.3 Data confidentiality

Data confidentiality

Participant confidentiality is strictly held in trust by the CPI, participating investigators, research staff, and the MCRI and their agents. This confidentiality is extended to cover testing of biological samples in addition to the clinical information relating to participating participants.

To preserve confidentiality and reduce the risk of identification during collection, analysis and storage of data and information, the following will be undertaken:

(1) The number of private/confidential variables collected for each individual has been minimised. The data collected will be limited to that required to address the primary and secondary objectives

(2) Participant data and samples will be identified through use of a unique participant study number assigned to the study participant ("re-identifiable").

The CPI is responsible for the storage in REDCap of a master-file of identifiable data with the participant ID; access is managed by restricting user permissions to members of the research team and authorised persons.

(3) Separation of the roles responsible for management of identifiers and those responsible for analysing content. The data will be analysed by members of the research team who will be provided with anonymised data identified only by the unique participant study ID.

9.2.4 Quality assurance

A REDCap data dictionary with range checks will be used to minimise data entry errors, such as out-of-range values. Data quality control checks (e.g. checking for invalid characters, invalid dates, data that is not consistent with data in other data fields) and data cleaning will be done by trained members of the research team on a regular basis. Any discrepancies will be reported to the CPI or delegate and addressed in a timely manner.

Quality control checks will be run by the data team, on a regular basis, who will highlight any queries to the CPI, RPI and SPI.

9.2.5 Archiving - Data and document retention

Upon completion of the study, data will be stored securely on MCRI server (restricted access) and/or locked in secure cabinet in MCRI laboratories (for hardcopy data) for at least 15 years after study completion, in accordance with the requirements of the Therapeutic Goods Administration and Health Privacy Principles and any other relevant regulatory authorities.

Prof Nigel Curtis (CPI) will be the custodian during the archive period, and members of the research team will have access to the stored data. If the CPI becomes unable to perform this

Pittet LF, et al. BMJ Open 2023; 11:e052101. doi: 10.1136/bmjopen-2021-052101

task all responsibilities of the custodian will fall to the sponsor (MCRI). At the end of the archival period, long-term retention of the data may occur.

Records should not be destroyed without the written consent of the Sponsor. The Sponsor will inform Site Principal Investigators when these documents no longer need to be retained.

9.2.6 Data sharing

Data sharing

De-identified data will be deposited on a recognised clinical trials data sharing repository and transferred to the Bill and Melinda Gates foundation.

Under the data sharing agreement with the Bill and Melinda Gates foundation the BRACE project will:

1. Register with and upload documents to clinicaltrials.gov
    - Study Protocol
    - Statistical Analysis Plan
    - Case Report Form Template(s)
    - Data Dictionary
    - Informed Consent Agreement Template
2. Share with the Gates Medical Research Institute the following documents;
    - Randomization Plan
    - Data Management Plan
    - Edit Check Specifications
    - Case Report Form Template (s) and Completion Guidelines
    - Data Transfer Agreements
    - All data generated by investigators funded by the Bill and Melinda Gates Foundation. This data will be de-identified.
3. All documents listed above will be available after being posted on a clinical trials data sharing repository and deidentified patient data related to the outcomes listed in the protocol will also be transferred to this repository.

Participant consent to the data sharing requirements is a mandatory requirement in updated Master PICF v8. Participants consented under earlier versions (prior to v7) of the PICF will be advised about the updated data sharing requirements and given an opportunity to opt-out via email of the data sharing arrangement. Data will not be shared, where participants have specifically requested their data not be shared.

Access to deidentified patient data in the data sharing repository and the Bill and Melinda Gates Foundation will be limited to ethically approved research and subject to the governance procedures of the data repository and the Bill and Melinda Gates Foundation

respectively. The governance procedures ensure the data are accessed for scientifically sound research.

After database lock, the following may be made available long-term for use by future researchers from a recognised research institution whose proposed use of the data has been ethically reviewed and approved by an independent committee and who accept MCRI's conditions, under a collaborator agreement, for accessing:

- Individual participant data that underlie the results reported in our articles after de-identification (text, tables, figures and appendices)
- Study protocol, Statistical Analysis Plan, PICF

9.2.7 Long-term custodianship (after archive period finished)

Prof Nigel Curtis will be the long-term custodian following the archive period. If he is unable to perform this task the responsibility of custodianship will fall to the sponsor (MCRI).

9.2.8 Data retrieval and linkage

The present study expects that it will acquire some research data from existing administrative and service data sources. In some instances, participant consent may allow retrieval of datasets without the need for linkage keys, as has usually been the case of other MCRI studies.

For datasets in Australia the study will work with organisations such as, the Centre for Victorian Data Linkage, the Australian Institute of Health and Welfare and the Population Health Research Network that supports data linkage and integration services within and between jurisdictions. For private sources such as pathologists, the study will establish appropriate initiatives.

Brazil, this data can be retrieved, if necessary, from the national government information systems, such as E-SUS, SIVEP-GRIPE, GAL and electronic medical records from SESAU / CG / MS (Municipal Health Secretariat of Campo Grande / MS, through unique identifiers of the participant (registration in the Individual Taxpayer Register - CPF and SUS CARD).

We anticipate data linkage and access will occur after the study recruitment period is complete, but the exact timing is yet to be determined. Working with both the capabilities of the data linkage services and through consultation with research studies that have extensive data linkage experience, this study will establish IT systems and SOPs to support data linkages processes that are efficient and minimise the risk of disclosure. These processes will use data linkage keys to separate the personally identifiable information needed for data linkage from the administrative and clinical data being sourced.

9.2.9 Sample management: Additional data management considerations

Data and information for biospecimens will be managed as above with the additional considerations.
Data collection: de-identified sample data may also be stored in OpenSpecimen (restricted access stored on secured servers at each study laboratory site), other site-specific electronic laboratory information management systems (LIMS, restricted access) or hard copy . Where biospecimen data is stored in OpenSpecimen or site-speficic LIMS, data will be transferred to

the MCRI servers on a regular basis. Where biospecimen data is collected on hardcopy, data will be transcribed to REDCap on a regular basis.

9.2.10 Sample management: Specimen collection & storage.

Biospecimens will be processed, stored and data will be recorded at laboratory study sites. Samples will be identified using barcoded tubes or with the unique participant study ID, and year of birth. No identifying information will be stored on biospecimen labels. Biosamples will be stored securely at laboratory study sites in temperature-controlled freezers and liquid nitrogen tanks as appropriate for the sample type. Access to biosamples will be restricted to the study team. The samples will be used for the analyses specified in the protocol. Samples will be shipped from study sites to MCRI for long term storage. For tests that require equipment or technical expertise not available in Melbourne, select specimens may be sent to collaborating laboratories outside of Melbourne (interstate and/or overseas) for further testing. These samples may be shipped from MCRI or directly from study sites if they have not yet been shipped to MCRI. Shipment of samples to MCRI or collaborating laboratories doing testing will be done by International Air Transport Association (IATA) accredited staff with temperature control (e.g. ice pack, dry ice) as appropriate for the sample type.

The biosamples will be retained long-term according to the banking management detailed below. As per data, Prof Nigel Curtis (CPI) will be the custodian of the biosamples during the archive period.

9.2.11 Sample management: Specimen & Biobanking

All samples that are not used immediately for the laboratory assessments described in previous sections, may be cryopreserved for an indefinite period of time to enhance the possible benefit from this study, by providing a sample biobank that may be used for research related to immunology or infectious diseases, in the future. The biobank will be at MCRI laboratories (Infectious Diseases Group) in Melbourne, (please see Appendix 1 for Biobank Registration Form). The biobank will be registered with the Melbourne Children's Bioresource Centre (MCBC). Written informed permission (extended consent) for banking of specimens and future use for study objectives without further consent will be obtained from the participant. These samples may be used for additional research studies related to immunology or infectious diseases. For tests that require equipment or technical expertise not available in Melbourne, select specimens may be sent to collaborating laboratories outside of Melbourne (interstate and/or overseas) for further testing.

Databank is defined as: "A systematic collection of data, whether individually identifiable, re-identifiable or non-identifiable" (NHMRC National Statement on Ethical Conduct in Human Research)

Biobank is defined as: ".... collections of human biological materials (biospecimens) linked to relevant personal and health information (which may include health records, family history, lifestyle and genetic information) and held specifically for use in health and medical research." (NHMRC Biobanks Information Paper 2010)

Pittet LF, et al. BMJ Open 2023; 11:e052101. doi: 10.1136/bmjopen-2021-052101

10 TRIAL OVERSIGHT

10.1 Governance structure

10.1.1 Trial Steering Committee (TSC)

The trial steering committee will be made up of representatives from the key stakeholders and the chief principal investigator along with independent content expert (s).

10.1.2 Independent Data and Safety Monitoring Board (DSMB)

An independent Data and Safety Monitoring Board (DSMB) will be convened three times during the study: at 3 and 9 months post initial recruitment, and once there have been 100 severe case of COVID-19 disease.

The DSMB at 3 and 9 months will monitor safety (including number of deaths and number of ICU admissions), data completeness, and the general study conduct.
A third DSMB is planned once there have been 100 cases of severe COVID-19 disease. This interim analysis will primarily be on a comparison of the number of cases of severe COVID-19 disease (primary outcome (2)) between the BGG group (irrespective of whether the participants received flu vaccine at randomisation) and the control group (irrespective of whether the participants received flu vaccine or placebo at randomisation), although data will also be presented on COVID-19 disease (primary outcome (1)) and also separately for those recruited prior to and post the introduction of the placebo to provide the DSMB with a complete picture. The DSMB will be given a stopping rule, but since the pandemic is rapidly evolving, the global situation should be considered with the context of any apparent differences. More information of this efficacy interim analysis is explained in section 11.4 of this current Protocol.

All the details of the DSMB analyses will be outlined in the DSMB charter.

The DSMB will be composed of individuals with the appropriate expertise, including at least three independent clinicians and/or biostatisticians who, collectively, have experience in the management of biostatistics and the conduct and monitoring of randomised controlled trials. Members of the DSMB will be independent of trial conduct. The DSMB will review data from each intervention group of the trial in a semi-blinded fashion. The DSMB will provide its input to the CPI.

10.1.3 Independent Safety Monitor

During the start of the recruitment period (until August 2020) an independent safety monitor will review a report of SAE and specified non-serious adverse events of interest on a weekly basis and report any concerns to the Sponsor-Investigator. For the remainder of the recruitment period, the monitor will review such reports monthly. This role will cease once recruitment is complete.

10.1.4 Quality Control and Quality Assurance

Both the Chief Principal Investigator and Site Investigators have responsibilities in relation to quality management.

The Chief Principal Investigator will ensure the development of procedures that identify, evaluate and control risk for all aspects of the study, e.g. study design, source data management, training, eligibility, informed consent and adverse event reporting. The Chief Principal Investigator will ensure the implementation of quality control (QC) procedures, which will include the data entry system and data QC checks. Any missing data or data anomalies will be communicated to the site(s) for clarification/resolution.

The Site Principal Investigator l will be responsible to ensure the verification that the clinical trial is conducted and data are generated and biological specimens are collected, documented (recorded), and reported in compliance with the protocol, good clinical practice and applicable regulatory requirements. In some regions a subcontracted monitor may be engaged by MCRI as needed.

In the event of non-compliance that significantly affects human participant protection or reliability of results, the Chief Principal Investigator (or delegate) and/or Site Principal Investigator (or delegate) will perform a root cause analysis and corrective and preventative action plan (CAPA).

In addition, each clinical site will perform internal quality management of study conduct, data and biological specimen collection, documentation and completion. An individualised quality management plan will be developed to describe a site's quality management.

11 STATISTICAL METHODS

11.1 Sample Size Estimation

7244 healthcare workers will be enrolled in the trial outlined in this protocol, although data from this trial will be combined with the data from the 2834 participants enrolled into the first stage of this study which followed an identical protocol but where participants were randomised between BCG and no BCG which was given concurrently with the flu vaccination, resulting in a total sample size of 10078 participants.

Participants will be randomly allocated in a 1:1 ratio to BCG vaccine group (n=3622, plus 1417 from the first stage who received flu vaccine at time of randomisation), and to control (n=3622, plus 1417 from the first stage who received flu vaccine at time of randomisation and no 0.9% NaCl placebo). This sample size was calculated based on the two primary outcomes of (1) number of participants with COVID-19 disease and (2) number of participant with severe COVID-19 disease. Since the study aims to assess two primary outcomes, an adjustment for multiplicity will be applied to maintain a global Type I error rate of 5% by splitting of this alpha.

For the primary outcome (1), the number of participants with COVID-19 disease: it is conservatively estimated that a proportion of 55% of subjects will be infected by COVID-19 disease in the placebo group; applying a 1:1 ratio for randomisation, a total sample size of n=2016 (1008 group) will provide 95% power with 2-tailed 0.005 significance level (10% of

the global significance level) for the Pearson chi-square test (with continuity correction) to detect an absolute difference of 10% between an incidence of COVID-19 disease of 45% in the BCG vaccine group and 55% in the placebo group.

For the primary outcome (2), the number of participants with severe COVID-19 disease at 6 months, we powered the study to identify a risk ratio of 0.67 in the BCG compared with the placebo group for severe COVID-19 disease at 6 months (which is much more realistic than a risk ratio of 0.5 as per the original sample size). Assuming that 4% of subjects will be infected by severe COVID-19 disease by 6 months in the control group, a total sample size of n= 6076 (3038 per group) will provide 80% power with 2-tailed 0.04 significance level (80% of the global significance level) for the Pearson chi-square test to detect a risk ratio of 0.667, equivalent to an absolute difference of 1.3%. Note this calculation was conducted using an alpha of 0.04 to allow the remaining 0.01 to be spent on primary outcome (1) (alpha=0.005) and the interim analysis (alpha=0.005, see section 11.4 for details of the interim analysis. Allowing for a 16% loss to follow up, it is planned that the study will recruit 7244 healthcare works.

In the pre-planned meta-analysis, we will have a sample size of 10,078 participants (7244+2834), or 8062 participants allowing for an overall 20% loss to follow up. For the combined analysis it is expected that the drop-out will be slightly higher (20% instead of 16%) because it also includes participants recruited prior to the introduction of the placebo, ie not placebo controlled. Again assuming that 4% of subjects will be infected by severe COVID-19 disease by 6 months in the control group, a total sample size of n=8062 (4031 per group) will provide 90% power with 2-tailed 0.04 significance level for the Pearson chi-square test to detect a risk ratio of 0.667, equivalent to an absolute difference of 1.3%. This will be a secondary analysis of the final study report.

11.2 Population to be analysed

The primary analysis of all outcome data will be an intention-to-treat (ITT) analysis including all randomised participants, regardless of whether they received trial drug.

11.2.1 Handling of missing data

For the primary analysis the imputation of missing data will only be considered if 10-20% of the primary outcome is missing and will be undertaken using multiple imputation (MI) models. Multiple imputation analysis will be performed on the ITT population. The frequency and patterns of missing data will be examined. Multiple imputation models will be conducted separately in the two treatment groups using chained equations applied to all outcomes, including baseline measures, as auxiliary variables. Fifty imputed datasets will be generated including all randomised subjects.

11.3 Methods of analysis

Data analysis for the study will be performed by CEBU at MCRI. Ms Francesca Orsini has been appointed for the trial.

Statistical analysis will follow standard methods for randomised trials and the primary analysis will be by intention to treat (ITT), including all randomised participants.

Categorical variables will be presented as the number and proportion in each category. Continuous variables will be presented as means and standard deviations (SDs), or medians and interquartile ranges for skewed data, and the range.

PRIMARY ANALYSIS. Comparison between the BCG and placebo groups in the proportions of participants with COVID-19 disease (primary outcome 1), as well as in the proportions of participants with severe COVID-19 (primary outcome 2), will be presented as the absolute risk difference (RD) as well as the risk ratio (RR) at 6 months and their 95% confidence interval (CI), obtained using a generalised linear model, with adjustment for the strata (defined by site, age and presence of comorbidity) used in the randomisation. The same analysis will be repeated on the same outcomes at 12 months. As secondary analyses the same models will be run to include also the following covariates: gender, number and type of comorbidities, whether already vaccinated for BCG in the past, and any other factor that may show imbalance between the groups at baseline.

A secondary analysis will be performed as above on the total 10078 participants, comparing all the participants who were randomised to BCG (irrespective of whether they received flu vaccine at randomisation) and those randomised to control (irrespective of whether they received flu vaccine or placebo at randomisation). Analysis will be as described above, but also adjusted for being in the initial stage of the study. As part of this analysis we will conduct an exploratory analysis of whether the treatment effect varies between the two stages of the study (prior to and post the introduction of the placebo) by including an interaction between treatment and study stage. Results will be interpreted with caution given that the study is underpowered for this comparison.

SECONDARY OUTOMES. According to the nature of the secondary outcomes to be analysed (binary, continuous or categorical) the appropriate generalised linear model (GLM) will be used to estimate the effect of the BCG vaccine on the outcome of interest compared to the control group. All analyses will be adjusted for the stratification factors used in the randomisation (site, age and presence of comorbidity). As secondary analyses the same models will be run to include the following covariates: sex, number and type of comorbidities, whether already vaccinated for BCG in the past, and any other factor that may show imbalance between the groups at baseline.

Survival analysis techniques will be adopted to analyse time to event data.

A secondary analysis will be conducted on the total 10078 participants using the same methodology but also adjusted for being in the initial stage of the study. We will conduct an exploratory analysis of whether the treatment effect varies between the two stages of the study by including an interaction between treatment and study stage.

Sub-group analyses will be undertaken on outcomes of those who:

- Had previous BCG vaccine before enrolling into the trial
- Had a positive serology to SARS-CoV-2 when enrolling into the trial

The full details for each variable will be included in the Statistical Analysis Plan (SAP).

11.4 Interim Analyses

As part of the interim monitoring there will be a single formal interim analysis of the efficacy data. This interim analysis will primarily be on a comparison of the number of cases of severe COVID-19 disease (primary outcome (2)) between the BGG group (irrespective of whether the participants received flu vaccine at randomisation) and the control group (irrespective of whether the participants received flu vaccine or placebo at randomisation), although data will also be presented on COVID-19 disease (primary outcome (1)) and also separately for those recruited prior to and post the introduction of the placebo to provide the DSMB with a complete picture. The timing of the interim analysis will be event driven, and will be conducted using a time-to-event analysis, censoring participants who have not had the event at the time of their last follow-up. This data will be used to provide Kaplan-Meier estimates of the survival curve in the BCG and control groups, which will be used to estimate the proportion with severe COVID-19 disease at 6 months. These proportions will be used to compare the two groups.

We have allocated alpha=0.005 to the interim analysis using the conservative approach of splitting the alpha allocated to primary outcome (2) between the interim and final analysis. Under the original sample size calculation, with 1668 per group and an incidence of 4% in severe COVID-19 disease at 6 months in the control group and 2% in the intervention group, this would equate to 67 + 33 = 100 cases in total. We therefore plan to conduct a formal interim analysis of severe COVID-19 disease once there have been 100 cases of severe COVID-19 disease. This will include all randomised participants irrespective of whether they received flu vaccine at randomisation if randomised to BCG and irrespective of whether they received flu vaccine or placebo at randomisation if randomised to control.

This interim analysis of the severe COVID-19 disease will be performed on the all of the participants randomised up to the interim analysis time point, comparing all the participants who were randomised to BCG (irrespective of whether they received flu vaccine at randomisation) and those randomised to control (irrespective of whether they received flu vaccine or placebo at randomisation). The DSMB will also be given information on which participants belong to the first stage of the study.

With 100 events, we will have 72% power to detect a risk ratio of 0.5 in the incidence of severe COVID-19 disease at 6 months at the interim analysis based on a two-sided test of alpha=0.005, which is a reasonable power for this interim analysis. The stopping rule for the interim analysis will therefore be p<0.005.

Given the dynamic nature of research in this field, the DSMB will be advised that this rule be used as a guideline rather than a formal rule, and should be interpreted in the context of external information and information on the efficacy of BCG vaccination on the incidence of COVID-19 disease (primary outcome (1)) which will also be presented in the DSMB report using the same methodology.

Pittet LF, et al. BMJ Open 2023; 11:e052101. doi: 10.1136/bmjopen-2021-052101

12 ETHICS AND DISSEMINATION

12.1 Research Ethics Approval & Local Governance Authorisation

This protocol and the informed consent document and any subsequent amendments will be reviewed and approved by the applicable human research ethics committee (HREC) prior to commencing the research at each site. A letter of protocol approval by HREC will be obtained prior to the commencement of the trial, as well as approval for other trial documents requiring HREC review.

12.2 Amendments to the protocol

This trial will be conducted in compliance with the current version of the protocol. Any change to the protocol document or Informed Consent Form that affects the scientific intent, trial design, participant safety, or may affect a participants willingness to continue participation in the trial is considered an amendment, and therefore will be written and filed as an amendment to this protocol and/or informed consent form. All such amendments will be submitted to the HREC, for approval prior to being implemented.

12.3 Protocol Deviations and Serious Breaches

All protocol deviations will be recorded in the participant record (source document) and on the CRF and must be reported to the CPI or delegate, who will assess for seriousness.

Those deviations deemed to affect to a significant degree rights of a trial participant or the reliability and robustness of the data generated in the clinical trial will be reported as serious breaches. Reporting will be done in a timely manner (the CPI or delegate to review and submit to the approving HRECs within 7 days, or as required).

Where non-compliance significantly affects human participant protection or reliability of results, a root cause analysis will be undertaken and a corrective and preventative action plan prepared.

Where protocol deviations or serious breaches identify protocol-related issues, the protocol will be reviewed and, where indicated, amended.

13 CONFIDENTIALITY

Participant confidentiality is strictly held in trust by the participating investigators, research staff, and the sponsoring institution and their agents. This confidentiality is extended to cover testing of biological samples in addition to the clinical information relating to participating participants.

The trial data and all other information generated will be held in strict confidence. No information concerning the trial or the data will be released to any unauthorised third party, without prior written approval of the sponsoring institution. Authorised representatives of the sponsoring institution may inspect all documents and records required to be maintained by the Investigator. The clinical trial sites will permit access to such records.

All laboratory specimens, evaluation forms, reports and other records that leave the site will be identified only by the Participant Identification Number (SID) to maintain participant confidentiality.

Clinical information will not be released without written permission of the participant, except as necessary for monitoring by HREC or regulatory agencies.

14 PARTICIPANT REIMBURSEMENT

In Australia and Europe, participants will not be reimbursed for their involvement.

As outlined in appendix 4 in Brazil in line with federal legislation, expenses resulting from participation in the study, such as transportation to the place where the vaccination will be carried out will be reimbursed. The amount will not be considered substantial and reimbursement system will be designed to reduce risk of reimbursement being considered compensation or inducement to participant in the trial.

15 FINANCIAL DISCLOSURE AND CONFLICTS OF INTEREST

This is an investigator-initiated study, and the funders will have no role in the study design, data collection and analysis, decision to publish, or preparation of the manuscript.. MCRI holds no commercial interest in the manufacture and trade of BCG.

16 DISSEMINATION AND TRANSLATION PLAN

The results of the trial will be reported to the participants after analysis is complete. The results of this trial will be submitted to peer reviewed journals, presented at conferences and may form part of student theses.

The Chief Principal Investigator holds primary responsibility for publication of the results of the trial.

17 REFERENCES

1. Novakovic B, Messina N, Curtis N. Chapter 6 - The Heterologous Effects of Bacillus Calmette-Guérin (BCG) Vaccine and Trained Innate Immunity. In: Faustman DL, editor. The Value of BCG and TNF in Autoimmunity (Second Edition). Second edition. ed: Academic Press; 2018. p. 71-90.
2. World Health Organisation. Situation report - 197: World Health Organisation; 2020. Report No: 197.
3. Anderson RM, Heesterbeek H, Klinkenberg D, Hollingsworthd T. How will country-based mitigation measures influence the course of the COVID-19 epidemic? The Lancet. 2020.
4. SARS (Severe Acute Respiratory Syndrome). World Health Organisation, 2020. [https://www.who.int/ith/diseases/sars/en/, accessed 11/03/2020]
5. Middle East respiratory syndrome coronavirus (MERS-CoV). World Health Organisation, 2020. [https://www.who.int/en/news-room/fact-sheets/detail/middle-east-respiratory-syndrome-coronavirus-(mers-cov), accessed 11/03/2020]
6. World Health Organisation. Report of the WHO-China Joint Mission on Coronavirus Disease 2019 (COVID-19) World Health Organisation; 2020.
7. Elguero E, Simondon KB, Vaugelade J, Marra A, Simondon F. Non-specific effects of vaccination on child survival? A prospective study in Senegal. Tropical medicine & international health : TM & IH. 2005;**10**:956-60.

8. Higgins JP, Soares-Weiser K, Lopez-Lopez JA, et al. Association of BCG, DTP, and measles containing vaccines with childhood mortality: systematic review. BMJ. 2016;**355**:i5170.
9. Kristensen I, Aaby P, Jensen H. Routine vaccinations and child survival: follow up study in Guinea-Bissau, West Africa. BMJ (Clinical research ed). 2000;**321**:1435-8.
10. Nankabirwa V, Tumwine JK, Mugaba PM, Tylleskar T, Sommerfelt H. Child survival and BCG vaccination: a community based prospective cohort study in Uganda. BMC public health. 2015;**15**:175.
11. Vaugelade J, Pinchinat S, Guiella G, Elguero E, Simondon F. Non-specific effects of vaccination on child survival: prospective cohort study in Burkina Faso. BMJ (Clinical research ed). 2004;**329**:1309.
12. Hollm-Delgado MG, Stuart EA, Black RE. Acute lower respiratory infection among Bacille Calmette-Guerin (BCG)-vaccinated children. Pediatrics. 2014;**133**:e73-81.
13. de Castro MJ, Pardo-Seco J, Martinon-Torres F. Nonspecific (Heterologous) Protection of Neonatal BCG Vaccination Against Hospitalization Due to Respiratory Infection and Sepsis. Clin Infect Dis. 2015;**60**:1611-9.
14. Arts RJW, Moorlag S, Novakovic B, et al. BCG Vaccination Protects against Experimental Viral Infection in Humans through the Induction of Cytokines Associated with Trained Immunity. Cell Host Microbe. 2018;**23**:89-100 e5.
15. Nemes E, Geldenhuys H, Rozot V, et al. Prevention of M. tuberculosis Infection with H4:IC31 Vaccine or BCG Revaccination. N Engl J Med. 2018;**379**:138-49.
16. Wardhana, Datau EA, Sultana A, Mandang VV, Jim E. The efficacy of Bacillus Calmette-Guerin vaccinations for the prevention of acute upper respiratory tract infection in the elderly. Acta Med Indones. 2011;**43**:185-90.
17. Moorlag S, Arts RJW, van Crevel R, Netea MG. Non-specific effects of BCG vaccine on viral infections. Clin Microbiol Infect. 2019;**25**:1473-8.
18. Freyne B, Marchant A, Curtis N. BCG-associated heterologous immunity, a historical perspective: intervention studies in animal models of infectious diseases. Trans R Soc Trop Med Hyg. 2015;**109**:287.
19. Kleinnijenhuis J, Quintin J, Preijers F, et al. Bacille Calmette-Guerin induces NOD2-dependent nonspecific protection from reinfection via epigenetic reprogramming of monocytes. Proc Natl Acad Sci U S A. 2012;**109**:17537-42.
20. Kleinnijenhuis J, Quintin J, Preijers F, et al. BCG-induced trained immunity in NK cells: Role for non-specific protection to infection. Clin Immunol. 2014;**155**:213-9.
21. Kleinnijenhuis J, Quintin J, Preijers F, et al. Long-lasting effects of BCG vaccination on both heterologous Th1/Th17 responses and innate trained immunity. J Innate Immun. 2014;**6**:152-8.
22. Messina NL, Zimmermann P, Curtis N. The impact of vaccines on heterologous adaptive immunity. Clin Microbiol Infect. 2019;**25**:1484-93.
23. Zimmermann P, Donath S, Perrett KP, et al. The influence of neonatal Bacille Calmette-Guerin (BCG) immunisation on heterologous vaccine responses in infants. Vaccine. 2019;**37**:3735-44.
24. Zykov MP, Subbotina TI. Modulation of humoral immune response to influenza vaccines by BCG. Acta Virol. 1985;**29**:403-9.
25. Australian Technical Advisory Group on Immunisation (ATAGI). Australian Immunisation Handbook. Canberra: Australian Government Department of Health,; 2018.
26. Australian Government Department of Health. The BCG vaccine: information and recommendations for use in Australia March 2013.
27. Lotte A, Wasz-Hockert O, Poisson N, Dumitrescu N, Verron M, Couvet E. BCG complications. Estimates of the risks among vaccinated subjects and statistical analysis of their main characteristics. Adv Tuberc Res. 1984;**21**:107-93.

28. Bothamley GH, Cooper E, Shingadia D, Mellanby A. Tuberculin testing before BCG vaccination. BMJ (Clinical research ed). 2003;**327**:243-4.
29. Hendry AJ, Dey A, Beard FH, Khandaker G, Hill R, Macartney KK. Adverse events following immunisation with bacille Calmette-Guerin vaccination: baseline data to inform monitoring in Australia following introduction of new unregistered BCG vaccine. Communicable diseases intelligence quarterly report. 2016;**40**:E470-e4.
30. BCG Vaccine SSI data sheet 14 March 2018. Ltd SN, [https://www.medsafe.govt.nz/profs/datasheet/b/BCGCSLinj.pdf, accessed 24 Dec 2019]
31. Pilgrim S. Administration of the Bacillus Calmette Guerin (BCG) Vaccination in neonates. 2019.
32. Vaccine schedules in all countries of the European Union. European Centre for Disease Prevention and Control, 2020. [https://vaccine-schedule.ecdc.europa.eu/, accessed Apr 2020]
33. de Vries G, Riesmeijer R. National Tuberculosis Control Plan 2016-2020. Bilthoven BA, National Institute for Public Health and the Environment, 2016. [Available at https://www.rivm.nl/documenten/national-tb-control-plan-0, accessed March 2020]
34. World Health Organization. Bulletin of the World Health Organization 1995: World Health Organization; 1995 1995.
35. Fine P, Carneiro I, Milstien J, Clements JC. Issues relating to the use of BCG in immunization programmes: a discussion document. Geneva: Department of Vaccines and Biologicals, World Health Organization; 1999.
36. Paul Fine IC, Julie Milstien, C. John Clements. Issues relating to the use of BCG in immunization programmes: a discussion document. Geneva: Department of Vaccines and Biologicals, World Health Organization; 1999.
37. Immunisation schedules in the WHO European Region: World Health Organization; 1995.
38. Coulter C. Tuberculosis testing. Aust Fam Physician. 2012;**41**:489-92.
39. Randomised controlled trial of single BCG, repeated BCG, or combined BCG and killed Mycobacterium leprae vaccine for prevention of leprosy and tuberculosis in Malawi. Karonga Prevention Trial Group. Lancet. 1996;**348**:17-24.
40. Roth AE, Benn CS, Ravn H, et al. Effect of revaccination with BCG in early childhood on mortality: randomised trial in Guinea-Bissau. BMJ (Clinical research ed). 2010;**340**:c671.
41. Rakshit S, Ahmed A, Adiga V, et al. BCG revaccination boosts adaptive polyfunctional Th1/Th17 and innate effectors in IGRA+ and IGRA- Indian adults. JCI Insight. 2019;**4**.
42. Hatherill M, Geldenhuys H, Pienaar B, et al. Safety and reactogenicity of BCG revaccination with isoniazid pretreatment in TST positive adults. Vaccine. 2014;**32**:3982-8.
43. Rodrigues LC, Pereira SM, Cunha SS, et al. Effect of BCG revaccination on incidence of tuberculosis in school-aged children in Brazil: the BCG-REVAC cluster-randomised trial. Lancet. 2005;**366**:1290-5.
44. Cunha AJ, Sant'Anna CC, Mannarino R, Labanca TC, Ferreira S, March MF. Adverse effects of BCG revaccination: a report on 13 cases from Rio de Janeiro, Brazil. Int J Tuberc Lung Dis. 2002;**6**:1110-3.
45. Faustman DL. Type 1 Diabetes Reversal Trials at Massachusetts General Hospital. In: Hospital MG, editor. Massachusetts General Hospital: Massachusetts General Hospital; 2018.

17.1 Appendix 1: Specimens for biobanking - completed biobank registration form

**Document version & date**	Version 1.1 24th Aug 2020
**Name of the bank**	BCG vaccine to prevent severe COVID-19 disease in healthcare workers (BRACE)
**Custodian of the bank**	Name: Prof Nigel Curtis
**Purpose of the bank**	To store data and samples collected in the ':BCG vaccination to Reduce the impact of COVID-19 in healthcare workers (BRACE)' trial so they can be used in future research related to infectious diseases and immunity.
**Sample/data type(s) and where these will be accessed from and over what time period**	Data will be collected from<br>Questionnaires, Medicare records and test results obtained as part of the research project 'BCG vaccination to Reduce the impact of COVID-19 in healthcare workers (BRACE)', by members of the research team.<br>Blood and/or swab samples will be obtained via this research project also, and will be stored for an indefinite period of time.<br>The samples/data may be sent overseas for future research related to infectious diseases, immunology, or vaccines.<br>Data stored includes:<br>- Demographics (e.g. age, gender, date)<br>- Environment (e.g. household members, exposure to SARS-CoV-2 positive people, role in the hospital, TB exposure, previous vaccinations)<br>- Study outcome related data (e.g. SARS-CoV-2 test results, BCG and flu vaccine reactions, illnesses during study period, data generated from the laboratory analysis of samples collected)<br>Sample types stored:<br>- Swabs<br>- Plasma<br>- Serum<br>- Peripheral blood samples<br>- Granulocytes and whole blood<br>- Nucleic acid<br>After data ceases to be collected directly from participants, data may be obtained/generated via access to their medical records, government data sets or as samples are analysed and the data is added back into the data/biobank.
**Sample/data identifiability**	Clinical data in 'BCG vaccination to Reduce the impact of COVID-19 in healthcare workers (BRACE)' will be collected and stored

	in a REDCap database; a secure password-encrypted online database, or similar electronic database hosted by MCRI.<br>Data will be stored in re-identifiable format with the key held by the custodian or delegate. The REDCap database or comparable database will be hosted on the secure Murdoch Children's Research Institute (MCRI) server and backed up regularly by MCRI Information Technology.<br>Only members of the research team involved in data collection or data management will have access to the project's REDCap database or similar electronic database.<br>Samples will be stored (frozen) in re-identifiable format by using study ID number or tube barcodes.<br>All data associated with sample storage location and tracking will be stored in a separate REDCap database or similar electronic database. Access to this database is limited to members of the research team working in data/sample management or sample processing.<br>Laboratory generated data, any data collected outside of REDCap and data exported from REDCap or similar electronic database, will be stored in re-identifiable format by study ID. The data will be stored on the MCRI server in restricted folders on the Infectious Diseases group drive, as per MCRI policy.<br>Samples/data stored in re-identifiable format can be linked by the custodian or delegate to participants' identifiable information if it is ethically appropriate and required.
**Criteria for Bank participants**	Consenting to the project includes allowing the participants' data and samples to be used as defined in the protocol.<br>In addition there is an optional consent in the PICF for the storage of participants' biospecimens and participants' re-identifiable data for use in future research related to infectious diseases and immunity.<br>Inclusion criteria for Bank participants<br>- Recruited participant in the research project 'BCG vaccination to Reduce the impact of COVID-19 in healthcare workers (BRACE))'<br>- Provided informed consent for their data and samples to be stored for future ethically approved research (extended consent) related to infectious diseases and immunity.
**Access process for obtaining the samples/data**	Researchers must discuss their research plan with a member of the research team of the project 'BCG vaccination to Reduce the impact of COVID-19 in healthcare workers: (BRACE))'. The following will be taking into consideration:

	- Scientifically justifiable hypothesis and aims<br>- Study design is appropriate to achieve study aims<br>- Inclusion/exclusion criteria for participants appropriate to answer question<br>- If the research proposal is deemed to have merit, the researcher will complete a REDCap (or similar electronic database) access form detailing the proposed design, participants, data +/- samples that they would like access to.<br>This will be reviewed by the custodian (or delegate) of the data who will need to take into account the following, before approval is granted:<br>- Does the research plan involve research in the area of immunology or infectious diseases? If not, it is outside the scope of the data/biobank. To use the data one of the following will be required:<br>o a new project approved by the RCH HREC and participants contacted for their consent<br>o a new project approved by the RCH HREC and a waiver of consent granted<br>- Is the planned analysis feasible with the data/samples available in the data/biobank?<br>- Are there competing interests for the sample/data type in question?<br>- Is another researcher already analysing the data in a similar way and would collaboration on the existing project be more appropriate?<br>The access form for access to the data/biobank will be kept on the REDCap database or similar electronic database.
Sample and data input	Members of the research team working in data/sample management will input the data and samples to the data/biobank.
Location of the Bank	Samples will be stored in the MCRI freezer farm or in the Infectious Disease Group's freezers, and may be distributed to other collaborating laboratories where they may also be stored.<br>Data will be stored in a REDCap online database or similar electronic database, hosted on the secure Murdoch Children's Research Institute (MCRI) server, as well as in restricted electronic folders on the MCRI Infection and Immunity group drive.

**Confidentiality/security of samples/data**	Members of the research team of the project 'BCG vaccination to Reduce the impact of COVID-19 in healthcare workers (BRACE)' involved in data/sample collection or management will have open access to the bank data/samples.<br>No identifying data will be provided to researchers using data/samples from the biobank. To re-identify data/samples, the custodian (or delegate) will have access to the key, but will not pass this information onto researchers unless approved by ethics, or as required by law.<br>Data stored on REDCap database or similar electronic database will be password protected, and hosted on the secure MCRI server. This is backed up regularly by MCRI Information Technology.<br>The Bank will be secure against unauthorised access and passwords will be changed at regular intervals (as per MCRI policy).<br>The custodian (or delegate) will ensure removal of access to data once a project is finished or a researcher leaves the project.
**Destruction of samples/data**	Destruction of samples/data will occur upon participant request. This will be managed by the custodian (or delegate).
**Modifications to Bank Protocol**	If a change of purpose/data type/type of samples is to be considered, the custodian (or delegate) is required to submit to the HREC for approval and either contact the participants to obtain consent, or a waiver must have been granted.

17.2 Appendix 2. Collection of stool samples from a subset of BRACE participants

Background and Rationale:

For reasons that are poorly understood, B and T cell responses to vaccination (including BCG vaccination) are highly variable between individuals and between different populations. While many host factors, such as genetics, can influence inter-individual variation in these responses, increasing evidence shows that the gut microbiota, a large and diverse group of microorganisms that colonise gastrointestinal tract (GIT), plays a key role in shaping immune responses to vaccination (reviewed Lynn & Pulendran, 2017). For instance, in human infants, the relative abundance of several bacterial species in the stool microbiota has been associated with vaccine-specific IgG and T cell proliferation responses (Huda et al., 2014). Similarly, the composition of the stool microbiota in infants from rural Ghana was correlated with responses to the oral rotavirus vaccine (Harris *et al.,* 2017). Interestingly, germ-free mice have also been found to have impaired antibody responses to immunization with the model antigen ovalbumin (Lamousé-Smith *et al.,* 2011) and to the non-adjuvanted influenza vaccine (Oh *et al.,* 2014). Moreover, one of the principal investigators involved in this trial has recently found that, in mice, dysregulation of the microbiota leads to significantly impaired B and T cells responses to five different adjuvanted and live vaccines (including BCG) that are routinely administered to infants worldwide (Lynn *et al.,* 2018). Restoring the commensal microbiota rescued impaired responses (Lynn *et al.,* 2018). These data strongly suggest that the composition of the gut microbiota plays an important role in specific immune responses to vaccination. Whether the gut microbiota also influences non-specific effects of vaccines is currently unknown.

Primary objective of exploratory sub-study:

In a subset of BRACE trial participants consenting for an optional stool sample collection at baseline, determine whether the composition or metagenome-encoded function of the stool microbiota is correlated with either specific or non-specific immune responses to the BCG vaccine.

Secondary objectives of exploratory sub-study:

- Assess whether the composition of the stool microbiota is associated with any of the other primary, secondary, or exploratory outcomes described in the study protocol.
- Characterise the composition of the stool microbiota in participants in the trial and investigate whether the composition of the microbiota is altered at 3 or 12 months later.
- Assess whether immunisation with BCG leads to an altered microbiota at 3 or 12 months compared to participants receiving the placebo.

Outcomes:
Microbiota composition, including identities and the relative abundance of the bacteria present and their encoded microbial genes.

Population:
BRACE trial participants consenting for an optional stool sample collection at baseline, 3 months and 12 months.

Study Duration:
As per the BRACE trial protocol – 2 years.

Participant Duration:
12 months from randomisation.

Sub-study Locations:
Optional inclusion for Australian sites.

Sub-study Principal Investigator:
Prof. David J. Lynn BA MSc PhD
EMBL Australia Group Leader, Precision Medicine Theme, South Australian Health & Medical Research Institute, Adelaide, SA 5001.
Professor, College of Medicine & Public Health, Flinders University, Bedford Park, South Australia.
Email: *david.lynn@sahmri.com*

Potential risks and benefits:
Known potential risks:
This sub-study involves minimal risk to participants. Appropriate collection containers will be provided to participants to facilitate stool sample collection, storage and transport. A small stool sample will be collected by the participants at home. The tube contains a reagent that stabilises DNA at room temperature for up to 14 days. Participants will return the sample via a pre-paid addressed envelope. There will be no financial cost to the participant.

Known potential benefits:
This sub-study is exploratory in nature and is not expected to provide any direct additional benefit to the participants. The findings of the study, however, may be of significant value for understanding the role of the microbiota in influencing responses to vaccination.

Sub-study design:
Consent:
An additional option has been added to the BRACE online consent form to allow participants to optionally consent for a stool sample collection at baseline, 3 months and 12 months. The BRACE participant information and consent form (PICF) has been also modified to explain to participants the process for collecting stool samples and why they are being collected. If a participant declines to consent for stool sample collection this will not affect their participation in the BRACE trial (assuming all other inclusion and exclusion criteria are met).
Sample collection process:
Participants consenting for a stool sample collection will be provided with a collection pack at existing study visits at baseline, 3 months, and 12 months. The provided pack will contain: Instruction sheet, gloves, pathology stool pot, stool specimen collector tube and spoon set, protective plastic carrying tube, specimen bag, labels for identification of samples and pre-paid addressed envelope (for return postage). Participants will take the collection pack home with them and follow the following instructions to collect and return the stool sample.

Collection instructions:

1. Wash hands thorough and apply gloves.

2. Collect stool sample into the pathology stool pot within 1-3 days of study appointment.
   Note: Method of collecting the stool sample must prevent stool from falling into toilet water to avoid sample contamination.

3. Unscrew the stool specimen collector tube cap and use the spoon to scoop two spoonsful of stool (approximately 2 gram or 2mL in volume) from the sample.

4. Place the sample in the stool specimen collector tube.

5. Tighten the cap and shake to mix the contents thoroughly (invert 10 times) to create a suspension. Note: Some stool material may be difficult to re-suspend. As long as the material is suspended, the sample is stabilized. Foaming/ frothing during shaking is normal.

6. Dispose of gloves, unused stool material and the pathology stool pot and wash hands thoroughly.

7. Place stool specimen collector tube into the protective plastic carrying tube.

8. Place carrying tube into specimen carrier bag.

9. Place the sealed specimen bag containing the sample into the provided postage-paid reply envelop and post within 7 days of sample collection.

10. Samples will be returned to the nearest BRACE site laboratory for storage at -80C.

What we will do with the sample:

Briefly, samples will be collected at home by the study participants into Zymo fecal collection tubes which contain a reagent to stabilise DNA at ambient temperature. Samples were returned by mail within 2 weeks and stored at -80°C until processed. DNA will be extracted from pelleted samples using the appropriate DNA Isolation kit. We will perform 16S rRNA sequencing and/or metagenomic sequencing to profile the composition of the microbiota in the sample and the metagenome encoded by the microbiota. qPCR will be utilised to quantify bacterial load and quantify specific bacterial populations. We will then determine whether the composition or metagenome-encoded function of the stool microbiota is correlated with either specific or non-specific immune responses to the BCG vaccine. We will also assess whether the composition of the stool microbiota is associated with any of the other primary, secondary, or exploratory outcomes described in the study protocol. Furthermore, we will characterise the composition of the stool microbiota in participants in the trial and investigate whether the composition of the microbiota is altered at 3 or 12 months later. We will assess whether immunisation with BCG leads to an altered microbiota at 3 or 12 months compared to participants receiving the placebo.

References:

Lynn, D.J. and B. Pulendran, *The potential of the microbiota to influence vaccine responses.* J Leukoc Biol, 2017. **103**(2): p. 225-23

Huda, M.N., et al., *Stool microbiota and vaccine responses of infants.* Pediatrics, 2014. **134**: p. e362-72.

Harris, V.C., et al., *The infant gut microbiome correlates significantly with rotavirus vaccine response in rural Ghana.* J Infect Dis, 2017. **215**(1): p. 34-41.

Lynn, M.A., et al., *Early-Life Antibiotic-Driven Dysbiosis Leads to Dysregulated Vaccine Immune Responses in Mice.* Cell Host Microbe, 2018. **23**(5): p. 653-660 e5.

Oh, J.Z., *et al., TLR5- Mediated Sensing of Gut Microbiota Is Necessary for Antibody Responses to Seasonal Influenza Vaccination*. Immunity, 2014. ***41***: 478-492.

17.3 Appendix 3 UK Specific Requirements

In the UK, the Competent Authority (MHRA) required the following two UK specific requirements:

1. In the UK, a negative pregnancy test is required for all WOCBP to confirm eligibility for the trial.

2. In the UK, the responsibility to break the treatment code in emergency situations resides solely with the UK Principle Investigator and will not be delayed by requiring other study staff in Australia such as the Chief Investigator or medical monitor to be involved in the decision to un-blind. The study code will only be broken for valid medical or safety reasons e.g. in the case of a severe adverse event where it is necessary for a treating physician (Requester) to know which intervention the participant has received, in order to manage the participant's condition appropriately.

The Requester contacts the local Principal investigator (PI), or delegate, to discuss the pros and cons of breaking the code. If the consensus is to break the code, the Requester contacts the holder of the code break list. In the UK, this has been delegated to the UK based Data Manager who will provide the Requester with the information on allocated group on direction from the PI. On receipt of the allocation details the Requester deals with the participant's medical emergency as appropriate. Should this code-breaking protocol be activated, the Chief Investigator will be alerted at the earliest opportunity, and within 2 working days at the latest.

<u>Woman of Child Bearing Potential:</u>
For the purpose of this document, a woman is considered of childbearing potential (WOCBP), i.e. fertile, following menarche and until becoming post-menopausal unless permanently sterile. Permanent sterilisation methods include hysterectomy, bilateral salpingectomy and bilateral oophorectomy. A postmenopausal state is defined as no menses for 12 months without an alternative medical cause. A high follicle stimulating hormone (FSH) level in the postmenopausal range may be used to confirm a post-menopausal state in women not using hormonal contraception or hormonal replacement therapy. However in the absence of 12 months of amenorrhea, a single FSH measurement is insufficient.

Pittet LF, *et al. BMJ Open* 2023; 11:e052101. doi: 10.1136/bmjopen-2021-052101

17.4 Appendix 4 Brazil Specific Requirements

SARS-CoV-2 Screening test

Due to pubic interest in determining the extent of asymptomatic SARS-CoV-2 infection in healthcare workers in Brazil, the Brazilian investigators will use the BRACE participants to estimate this prevalence rate. Therefore after enrolment a baseline respiratory swab will be collected by the study nurse. The swab samples will be analysed by PCR for detection of SARS-CoV-2 and participants advised when results are confirmed. Participants who return a positive SARS-CoV-2 result on the baseline swab will remain in the trial. In Mato Grosso do Sul, the samples will be analysed in batch months after randomisation, so there will be no clinically actionable results. Results will be shared with participants approximately 3 months after randomisation, for participant who return a positive SARS-CoV-2 result, the site will be required to report the participant's positive SARS-CoV-2 results to the applicable health agencies. They will be told that they will not be informed of their result before then. In Rio de Janeiro, due to high transmission rates, samples will be tested immediately and reported to participants. PCR tests will be conducted by the study lab team and the results reported to health agencies by a system called e-SUS VS, which constitutes a database of several diseases, including COVID-19, which is mandatory.

IGRA

At randomisation, blood for IGRA will be taken for later assessment of seroconversion (production of specific anti-SARS-CoV-2 antibodies and IGRA TB). Therefore the initial blood sample in Brazil will be 35ml. This will identify participants who had TB exposure prior to commencement of the study. IGRA results will not exclude participants at consent & randomisation stage. Results will be shared with participants approximately 3 months after randomisation. A study doctor will follow-up with participants with positive IGRA to offer further assessment and treatment through government service provision.

Participant reimbursement

In Brazil, Resolution No. 466 of December 12, 2012 outlines the guidelines and regulatory standards for research involving humans in Brazil. This resolution outlines the requirement to provide reimbursement to participants and their companions, when necessary, such as transportation. In line with this requirement, participants in Brazil will receive reimbursement for relevant transportation costs for participation in the BRACE trial.

Safety Reporting

In Brazil, the RPI/s and SPI/s must comply with the safety reporting requirements of CEP/CONEP (defined in Circular Letter number 13). The HREC/s must be notified of all SAEs through the Brazil Platform (Notification), after the end of the event. The following timelines will be met for this study:

1. 30 days in case of fatal SAE occurring in a participant of the site in the jurisdiction of the HREC
2. 7 days in case of an SAE with a causal relationship with the investigational product, in a participant of the site in the jurisdiction of the HREC (Casual relationship means that the SAE is judged by either the reporting investigator or the sponsor as having a reasonable possibility of a causal relationship to a study vaccine).
3. 6 months for other SAE.

The RPI (or delegate) will notify SUSAR in Brazil to all investigators in their region, as appropriate. The RPI (or delegate) will report significant safety issues (including USM) to SPI in their region, the regulatory authority and applicable HREC/s in accordance with the requirements. The RPI (or delegate) will provide periodic reports of SAE (from Brazil trial sites) to the applicable regulatory authorities and/or HREC/s, as appropriate.

17.5 Appendix 5 The Netherlands Specific Requirements

The following changes will apply for the performance of the protocol in the Netherlands:

Statement of Compliance

This clinical trial will be conducted in compliance with all stipulations of this protocol, the conditions of the ethics committee approval, the NHMRC National Statement on ethical Conduct in Human Research (2007 and all updates), the Integrated Addendum to ICH E6 (R1): Guideline for Good Clinical Practice E6 (R2), dated 9 November 2016 annotated with TGA comments and the NHMRC guidance Safety monitoring and reporting in clinical trials involving therapeutic goods (EH59, 2016), and General Data Protection Regulation (GDPR) , as well as local laws and regulations, such as the Wet medisch-wetenschappelijk onderzoek met mensen (WMO).

2. Recruitment and consent

In Europe, due to ethics regulations, an electronic PICF will not be used and no information on eligibility nor any contact information will be collected prior to the informed consent process being finalized. When participants are interested in the study, they can verify their eligibility with the criteria listed on the website. Then, they will be shown a list of participating centers and advised to contact one of the centers directly to make an appointment for the first study visit. At this visit, the informed consent procedure will be completed in a face to face setting where the the PICF will be read and signed by both participant and investigator. Exact date of birth will not be collected in the eCRF for the study due to GDPR constraints; instead, year of birth (or 01-01-yyyy) will be used in the eCRF. Medicare card number is not applicable in Europe.

3. Data capture methods and data use, storage, access and disclosure during the trial

Archiving will be in compliance with NFU requirements: study data, source documents and the Study File will be kept for 25 years.
EU protocol addendum page_V2.0_20200629

4. COVID-19 testing will be performed via the national testing policy and therefore, the General Practitioner will be notified of the results by the organisation that performs the testing: GGD or the hospital that performs the test.

5. Sharing of contact information

In order to send out the 3, 6, 9, and 12 month questionnaires, the participant's email address will be collected in the RedCAP database. No other identifying information will be stored in the database for EU participants.

6. BCG vaccination is not expected to cause an exacerbation of the immune response with adverse consequences, because of 3 main arguments: • By activating anti-viral mechanisms, BCG decreases virus load and systemic inflammation (Arts et al, Cell Host Microbe 2018). Influenza pathophysiology is the same so if BCG had adverse effects, this would have been known for a long time. • Information is available on individuals vaccinated with BCG last year and no COVID19 complications were observed in this group.

17.6 Appendix 6 Spain Specific Requirements

The following changes will apply for the performance of the protocol in Spain:

1. Statement of Compliance

This clinical trial will be conducted in compliance with all stipulations of this protocol, the conditions of the ethics committee approval, the Integrated Addendum to ICH E6 (R1): Guideline for Good Clinical Practice E6 (R2), dated 9 November 2016 and General Data Protection Regulation (GDPR) , as well as local laws and regulations

2. Inclusion criteria

According to recommendations of the competent authority AEMPS (Agencia Española del Medicamento y Productos Sanitarios) If the patient is female, and of childbearing potential, she must have a negative pregnancy test (provided by Sponsor) at the time of inclusion and practice a reliable method of birth control for 30 days after receiving the BCG vaccination. - Woman of Childbearing Potential is defined as a premenopausal female who is capable of becoming pregnant.

3. Recruitment and consent

In Europe, due to ethics regulations, an electronic PICF will not be used and no information on eligibility nor any contact information will be collected prior to the informed consent process being finalized. When participants are interested in the study, they can verify their eligibility with the criteria listed on the website. Then, they will be shown a list of participating centers and advised to contact one of the centers directly to make an appointment for the first study visit. At this visit, the informed consent procedure will be completed in a face to face setting where the PICF will be read and signed by both participant and investigator.
Exact date of birth will not be collected in the eCRF for the study due to GDPR constraints; instead, year of birth (or 01-01-yyyy) will be used in the eCRF. Medicare card number is not applicable in Europe.

4. Data capture methods and data use, storage, access and disclosure during the trial

Archiving will be in compliance with NFU requirements: study data, source documents and the Study File will be kept for 25 years.

5. Sharing of contact information

In order to send out the 3, 6, 9, and 12 month questionnaires, the participant's email address will be collected in the RedCAP database. No other identifying information will be stored in the database for EU participants.

17.7 Appendix 7 Optional Biological sample collection during episodes of illness

Assessment of immune responses during episodes of illness will provide crucial insights into the mechanisms by which BCG may protect against COVID-19. BCG is proposed to protect against unrelated infections by boosting the innate immune response[1] which can directly protect against infections and also shape the adaptive immune response[2-3]. Biological samples collected after infection provide meaningful insight into the long-lasting effects of the infection and immune memory. However, they do not provide information about the early immune response to infection that can promote early clearance, may impact disease severity and may define the long-lasting memory response. It is this part of the immune response where BCG vaccination may play a crucial role in protection against COVID-19 as well as non-COVID-19 respiratory infections.

Objectives of exploratory sub-study

The additional collection of biological samples from BRACE participants during episodes of febrile or respiratory illness will contribute to the planned subgroup exploratory analyses of BRACE:

11. To determine the impact of BCG vaccination on the immune system that are associated with protection of adult healthcare workers from non-tuberculous infectious diseases including COVID-19.
13. To identify factors (e.g. age, sex, chronic conditions such as diabetes and cardiovascular disease, smoking, asthma, prior BCG vaccination, genetics, influenza vaccination, immunological/molecular factors) that influence adult immune responses and COVID-19 responses.

It will also contribute to the following additional exploratory objectives:
In a sub-set of BRACE trial participants who consent for an optional biological sample to be collected during episodes of fever or respiratory illness:

- To characterise the immune response to SARS-CoV-2 infection
- To compare immune responses during an episode of respiratory illness (COVID-19 or non-COVID-19 illness) in BCG-vaccinated and non-BCG vaccinated participants

Outcomes:
Immune system characterisation and molecular markers of disease in episodes or COVID-19 or non-COVID-19 respiratory or febrile illness from BCG-vaccinated and non-vaccinated participants.

Population: A sub-group of the BRACE trial participants who consent to an optional biological sample to be collected during episodes of fever or respiratory illness.

Study Duration:
As per the BRACE trial protocol – 2 years.

Participant Duration:
12 months from randomisation.

Sub-study Locations:
Optional inclusion for Australian sites.

Sub-study Principal Investigator:
Dr Nicole Messina
Senior Research Officer, Infectious Diseases Group, Murdoch Children's Research Institute, The Royal Children's Hospital, 50 Flemington Road Parkville, 3052 Victoria, Australia

Honorary fellow, Department of Paediatrics at Melbourne Children's Melbourne Medical School, Faculty of Medicine, Dentistry and Health Sciences, The University of Melbourne Email: nicole.messina@mcri.edu.au

Potential risks and benefits:

Known potential risks:

This sub-study involves minimal risk to participants. Having a blood test can sometimes cause some pain from the needle or be uncomfortable. Occasionally a small amount of bruising can occur on the skin where the blood was taken. Trained members of the study team will collect the blood samples from participants. Having a respiratory swab can sometimes be uncomfortable. Trained members of the study team will collect the respiratory swabs from participants. Self-testing swab kits may be provided as required, with clear instructions to participants on safe self-swabbing technique.

Known potential benefits:

This sub-study is exploratory in nature and is not expected to provide any direct additional benefit to the participants. The findings of the study, however, may be of significant value for understanding the immune response to COVID-19, the off-target effects of BCG vaccination on responses to COVID-19 and other respiratory infections and determinants of disease severity.

Sub-study design:

Consent:

An additional option has been added to the BRACE online consent form to allow participants to optionally consent additional biological sample collection during an episode of illness. The BRACE participant information and consent form (PICF) has been also modified to explain to participants the process for collecting these additional blood samples and why they are being collected. If a participant declines to consent for additional biological sample collection during an episode of illness this will not affect their participation in the BRACE trial (assuming all other inclusion and exclusion criteria are met).

Sample collection process:

Participants consenting for additional biological sample collection during an episode of illness may be contracted by the study team during any episode of respiratory or febrile illness that occurs during their involvement in the BRACE trial (i.e. up to 12 months from randomisation). Sample collection would occur during and up to one month after resolution of an episode of illness with fever or respiratory symptoms. The collection of samples will be done at a study site (e.g. if they are inpatients or obtaining SARS-CoV-2 testing at a study site) or at the participant's home, depending on the location of the participant.

Samples to be collected are:

-a blood sample

and/or

- saliva/respiratory swab/s

All samples will be collected, processed and stored in accordance with the BRACE trial protocol section 7.3. We will aim to take these samples at the same time as any other clinical or research samples where possible to minimise the number of sample collections for each participant, minimise contact of research staff with infectious patients and to reduce the need for research staff to use vital personal protective equipment (PPE).

What we will do with the sample:

Samples will be processed for analysis of the immune system as detailed in BRACE trial protocol section 3.2. Where indicated, saliva/respiratory swab/s collected will be linked with the relevant public health testing and reporting systems as BRACE trial protocol section 7.3. In addition, samples will be included in the BRACE biobank if participants have also consented for their samples being placed in the BRACE biobank.

References

1. Novakovic B, Messina N, Curtis N. Chapter 6 - The Heterologous Effects of Bacillus Calmette-Guérin (BCG) Vaccine and Trained Innate Immunity. In: Faustman DL, editor. The Value of BCG and TNF in Autoimmunity (Second Edition). Second edition. ed: Academic Press; 2018. p. 71-90.
2. Arts RJW, Moorlag S, Novakovic B, et al. BCG Vaccination Protects against Experimental Viral Infection in Humans through the Induction of Cytokines Associated with Trained Immunity. Cell Host Microbe. 2018;**23**:89-100 e5.
3. Kleinnijenhuis J, Quintin J, Preijers F, et al. Long-lasting effects of BCG vaccination on both heterologous Th1/Th17 responses and innate trained immunity. J Innate Immun. 2014;6:152-8.

17.8 Appendix 8 Optional Sub-study: collection of blood samples to measure immune responses to COVID-19 specific vaccines.

Sub study locations:
Australia
Brazil

Overview:
COVID-19-specific vaccines are becoming increasingly available and healthcare workers, being at high risk of SARS-CoV-2 exposure, are prioritised for receipt of these vaccines. BCG vaccination alters immune responses to subsequence vaccinations[1,2] and therefore it is plausible that it may boost the immune response to COVID-19-specific vaccines. As healthcare workers, participants in the BRACE trial will be prioritised for receipt of COVID-19-specific vaccines in most regions and as a result will likely receive these vaccines during their involvement of the BRACE trial.

The type of COVID-19-specific vaccine given to BRACE trial participants will vary between sites and it is likely that more than one type of vaccine will be used in a given region. The number of doses given (one or two) and the recommended interval between the two doses are likely to vary as well but are likely to be consistent within a given region.

The BRACE trial exploratory outcomes already include assessment of the effects of vaccines on the immune system (including the effects of BCG-vaccination on immune response to COVID-19-specific vaccines).

To ensure we obtain samples at the optimal times before and after COVID-19-specific vaccination, in a subset of participants, we propose collecting blood samples at up to three additional time-points:

- **(Visit 1, site specific)** prior to receipt of the first dose of a COVID-19-specific vaccine;
- **(Visit 2, site specific)** after the first dose of a COVID-19-specific vaccine.
- **(Visit 3)** 28 days after the second dose of a COVID-19-specific vaccine

These additional blood samples enable us to:

- screen for prior SARS-CoV-2 exposure (accounted for at analysis), and provide a baseline measure of the immune system prior to receipt of COVID-19-specific vaccines
- measure the immune response (e.g. antibodies) to the first and second dose of COVID-19-specific vaccines, and other changes in the immune system induced by the COVID-19-specific vaccine
- compare the vaccine responses to COVID-19-specific vaccines between the BCG and the control group to each COVID-19 specific vaccine
- compare our findings to other studies on COVID-19-specific vaccines[3]

Determining if BCG vaccination can improve the immune response to COVID-19-specific vaccines have important implications for the potential of BCG vaccination to increase efficacy of COVID-19-specific vaccines and may also impact our interpretation of the outcomes of the BRACE trial. This is particularly important for the COVID-19-specific vaccines that have a lower efficacy.

Objectives of exploratory sub-study

The additional collection of blood samples from BRACE trial participants immediately prior to, and after each COVID-19-specific vaccination will contribute to the existing planned subgroup exploratory analyses of BRACE:

1. *To determine the impact of BCG vaccination on the immune system that are associated with protection of adult healthcare workers from non-tuberculous infectious diseases including COVID-19.*
2. *To determine and compare changes in the immune system induced by vaccination of adult healthcare workers.*

Population: A sub-group of the BRACE trial participants who receive COVID-19-specific vaccines in regions taking part in the sub-study.

Outcomes: Immune system characterisation and molecular markers of immunity (including seroconversion to SARS-CoV-2) in response to COVID-19-specific vaccines in BCG-vaccinated and non-BCG-vaccinated participants.

Study Duration: As per the BRACE trial protocol – 2 years.

Participant Duration: Up to 4 months from sub-study inclusion

Sub-study Principal Investigator: Prof Nigel Curtis

Potential risks and benefits

Known potential risks

This sub-study involves minimal risk to participants. Having a blood test can sometimes cause some pain from the needle or be uncomfortable. Occasionally a small amount of bruising can occur on the skin where the blood was taken. Trained members of the study team will collect the blood samples from participants.

The amount of blood collected is too small to have any impact on the participants' health.
This sub-study will not impact the setting up of COVID-19-specific vaccination clinic at the participating sites. It is not expected to have any negative interactions between the BCG and the COVID-19-specific vaccine.

Known potential benefits

This sub-study is exploratory in nature and is not expected to provide any direct additional benefit to the participants. The findings of the study, however, may be of significant value for understanding the immune response to COVID-19-specific vaccines and the off-target effects of BCG vaccination on responses to COVID-19-specific vaccines.

Sub-study design

Eligibility:

Inclusion Criteria

- Participant in the BRACE trial who has previously consented to be contacted for future ethically approved projects.
- Participant recruited to the BRACE trial at a site taking part in this sub-study.

Exclusion Criteria

- A previous positive SARS-CoV-2 test at any time.
- Expected inability to provide a blood sample in the indicated time window after: the first dose (visit 2) and/or the second dose (visit 3) of a COVID-19-specific vaccine.
- [site specific]: Inability to provide a blood sample in the indicated time window prior the first dose (visit 1) of a COVID-19-specific vaccine.

Recruitment
Potential BRACE participants will be informed of this sub-study and invited to participate as per their recruitment sites' existing communication approach. BRACE participants will evaluate their eligibility for the sub-study and will have access to the site-specific participant information and consent form (PICF) prior to enrolment in the sub-study.

Consent
An additional participant information and consent form (PICF) will be provided to participants to allow them to optionally consent to this sub-study. If a participant declines to consent for this sub-study it will not affect their participation in the BRACE trial.

Data collection
Participants interested in this sub-study will be contacted by the study team to arrange blood collection if:

- The BRACE trial study site from which they were recruited begins COVID-19-specific vaccinations of staff

Or

- if the participants inform the BRACE trial team that they will receive a COVID-19 specific vaccine.

At these additional sub-study visits, participants will be asked about:

- prior positive COVID-19 tests,
- any other vaccines received since randomisation in BRACE (type, dose, route, date)
- expected date of vaccination with COVID-19-specific vaccine and which vaccine
- episodes of febrile or respiratory illness since last visit (if not already collected as part of the BRACE trial)
- (after vaccination only) adverse reaction to the COVID-19-specific vaccine

After the expected COVID-19-specific vaccine administration date, participants will be contacted as per their recruitment sites' existing communication approach, to confirm which vaccine they have received, where and when they received it, as well as when is the second dose planned.

Sample collection process
Sample collection will occur:

- **(Visit 1, site specific)** On the day of (or in the 5 to 14 days preceding) the first dose of a COVID-19-specific vaccine

  [site specific] *Note that for a participant who has already received their first dose of a COVID-19 specific vaccine, the participant's blood sample for the first timepoint will not need to be collected. However, blood samples for the remaining time points below will need to be collected.*
  It is planned to collect blood samples on the same day of vaccination, however we will accept

bloods that are taken up to 5 days before the first dose of COVID-19 specific vaccine in all regions, or even up to 14 days before the first dose of COVID-19 specific vaccine in regions where the COVID-19 prevalence is low, are acceptable.

- **(Visit 2, site specific)** 1 to 28 days (±2) days after the first dose of a COVID-19-specific vaccine

  Note that where the second dose of the COVID-19 specific vaccine is given within 28 days in a given region, this sample will be taken at an earlier time point. Efforts will be made to standardise the interval between the first dose of COVID-19-specific vaccine and the blood sample for each type of COVID-19-specific vaccine within each given region, eg within 14 (±2) days after the first dose of COVID-19-specific vaccine if the two doses of COVID-19-specific vaccine are given 2 weeks apart, or within 21 (±2) days after the first dose of COVID-19-specific vaccine if the two doses of COVID-19-specific vaccine are given 3 weeks apart.
  In specific sites, an earlier time-point (<7 days) will enable the exploration of the initial gene expression responses to vaccination.

- **(Visit 3)** 28 (±2) days after the second dose of a COVID-19-specific vaccine

  Note that efforts will be made to standardise the interval between the COVID-19-specific vaccine doses and the blood collection for both blood collections, for each type of COVID-19-specific vaccine and within a given region

Blood samples will be collected, processed and stored in accordance with the BRACE trial protocol section 7.3 with the exception that up to 40 mL of blood will be taken at each time point. Also, if this blood collection is done at the same time as a BRACE trial 3-monthly blood collection, an additional 10 mL of blood may be required for a total of 50 mL. The collection of blood samples will be done at a study site or at the participant's home, depending on the region. We will aim to collect these samples at the same time as the existing BRACE Trial 3-monthly blood samples where possible, to minimise the number of sample collections for each participant.

What we will do with the sample:
Samples will be processed for analysis of the immune system as detailed in BRACE trial protocol section 3.2. The immune system will be assessed by several methods, including:

a) measurement of antibodies to SARS-CoV-2 (to assess prior exposure/infection with SARS-COV-2) and their neutralisation ability
b) measurement of antibodies to COVID-19 specific vaccines (to determine seroconversion and antibody titres) and their neutralisation ability
c) characterisation of immune cell subpopulations
d) measurement of immune cell activation and differentiation
e) measurement of immune cell function (e.g. cytokine production and cell division) following *in vitro* stimulation with SARS-CoV-2, COVID-19-specific vaccines, or their components)

Sample size estimation:
As COVID-19-specific vaccines are novel, immune responses following vaccination have yet to be extensively characterised and there is currently no agreed correlate of protection. As such, formal sample size calculations are not possible. However, based on our previous experience assessing immune responses to other vaccines we estimate that for each region in which this sub-study will take place (e.g. Australia and Brazil) a sample size of 150 participants per randomisation group and

per COVID-19-specific vaccine type (aiming to have 100 participants with blood samples for all three timepoints) will be sufficient to detect a meaningful effect of BCG vaccination on the vaccine responses to COVID-19-specific vaccines. With the expectation that within a region the majority of participants will receive one of two vaccines we will recruit up to a total of 1200 participants: 150 participants x 2 randomisation groups (BCG or No BCG vaccination) x 2 regions (Australia and Brazil) with 2x COVID-19-specific vaccine types.

References

1. Messina NL, Zimmermann P, Curtis N. The impact of vaccines on heterologous adaptive immunity. *Clin Microbiol Infect* 2019; **25**(12): 1484-93.
2. Arts RJW, Moorlag S, Novakovic B, et al. BCG Vaccination Protects against Experimental Viral Infection in Humans through the Induction of Cytokines Associated with Trained Immunity. *Cell Host Microbe* 2018; **23**(1): 89-100 e5.
3. Ramasamy MN, Minassian AM, Ewer KJ, et al. Safety and immunogenicity of ChAdOx1 nCoV-19 vaccine administered in a prime-boost regimen in young and old adults (COV002): a single-blind, randomised, controlled, phase 2/3 trial. *Lancet* 2021; **396**(10267): 1979-93.

Standard Operating Procedures: BRACE Global - Code break procedures for BRACE

**Title:**	BRACE Global SOP - Code break procedures for BRACE
**Version:**	2.0
**Date:**	07 Oct 2020
**Institution Name**	Murdoch Children's Research Institute

**Author**<br>The author is signing to confirm the technical content of this study and the content.		
**Author Name and position**	**Signature**	**Date**
Francesca Orsini<br>Assistant Lead Biostatistician	Francesca Orsini<br>Francesca Orsini (Nov 16, 2020 16:45 GMT+11)	Nov 16, 2020
**Reviewer:**<br>The reviewers are signing to agree with the technical content of the document and that this document is ready for implementation.		
**Reviewer Name and position**	**Signature**	**Date**
Dr Laure Pittet<br>Data & Quality Lead	Laure Pittet<br>Laure Pittet (Nov 16, 2020 17:05 GMT+11)	Nov 16, 2020
**Approver:**<br>The approver is signing to confirm that the document has been reviewed and is approved for implementation.		
**Approval Name and position**	**Signature**	**Date**
Prof Nigel Curtis<br>Coordinating Principal Investigator	Nigel Curtis<br>Nigel Curtis (Nov 16, 2020 18:06 GMT+11)	Nov 16, 2020

C09 BRACE Global SOP Code break procedures for BRACE V2.0_7th Oct 2020

Background

Code break procedures have been established to ensure the safety of the participants involved in the trial, and at the same time to prevent the occurrence of unnecessary or unintentional un-blinding, in order to protect the integrity and validity of the data collected.

Procedure

1. The study codes are held in a secure area in REDCap with restricted access, called the un-blind database. The only individuals who can access it are: the un-blind data manager (Luke Stevens) and the immunisators. The data manager Luke Stevens has designed the un-blind database and is the only person able to access the codes at any time. Limited access to the codes is provided to the immunisator to whom permission has been delegated. This permission is documented on the delegation log, and is restricted by study site and in time. An immunisator is only allowed to look at the allocation group of a given participant just before administrating the intervention, on the day of randomisation.
2. The Chief Principal Investigator (Nigel Curtis) may only break the study code under the following circumstances; in an emergency or at the end of the study.

3. Breaking the blind in an emergency

   3.1. The study code should only be broken for valid medical or safety reasons e.g. in the case of a severe adverse event where it is necessary for an investigator or treating physician (Requester) to know which intervention the participant has received, in order to manage the participant's condition appropriately.
   3.2. The Requester contacts the local Principal investigator (PI), or delegate, to discuss the pro and cons of breaking the code. Together they contact the Chief PI, or delegate, to explain the reason for requiring a breaking of the code and make a recommendation, based on their discussion, on whether or not the code should be broken.
   3.3. The Chief PI, or delegate, decides on breaking the code or not. If there is a strong disagreement, another member of the Trial executive team (Andrew Davidson) can be contacted.
   3.4. If the consensus is to break the code, the Requester contacts the holder of the code break list (Luke Stevens). Luke Stevens should be contacted by email (luke.stevens@mcri.edu.au)
   3.5. Luke Stevens (or delegate) provides the Requester with the information on allocated group.
   3.6. On receipt of the allocation details the Requester deals with the participant's medical emergency as appropriate.
   3.7. If the Requester is not the site PI, the Requester must inform the site PI of the code break and the reasons for the actions taken as soon as possible.
   3.8. The site PI or delegate documents the breaking of the code and the reasons for doing so on the REDCap AE SAE form and in the site trial master file.
   3.9. A REDCap Protocol deviation form needs to be completed, indicating of the nature of the medical condition, and why it required the code to be broken.
   3.10. If the participant withdraws from the trial, this needs to be mentioned in the REDCap Participant status form via the selection of "Serious concurrent medical condition", indication of the nature of the medical condition, and why it required the code to be broken.
   3.11. All correspondences are archived in the trial master file.
   3.12. The site PI notifies the Trial Coordinator in writing as soon as possible following the code break detailing the necessity of the code break.
   3.13. The site PI notifies the Research Ethics Committee of the protocol deviation if required, and copies the letter to the Trial Coordinator

C09 BRACE Global SOP Code break procedures for BRACE V2.0_7th Oct 2020

3.14. MCRI notifies the code break to the RCH Research Ethics Committee in their annual report, and to the Data Safety Monitoring Committee at the next meeting.

4. Breaking the blind at 6-month time point for analysis purposes (primary outcomes)

4.1. The un-blinding of participants cannot occur until all participants have completed 6 months of follow-up post randomisation. In particular, un-blinding will occur after the database has been locked i.e. all data entered, validated and no further changes are expected. Furthermore, the person performing the statistical analysis will remain blinded until after the analysis has been completed.

4.2. The Chief PI contacts the Trial Coordinator to confirm that data collection is complete, provides the date of the last participant's 6 months exposure and requests permission for the un-blinding of the study.

4.3. The Trial Coordinator confirms that the study may be un-blinded by email to the Chief PI, copying in Luke Stevens (or delegate).

4.4. Luke Stevens (or delegate) provides treatment allocation details to the Chief PI as requested, who will then share it with the trial statistician for finalising the planned analysis.

4.5. No one else in the team will be notified of the individual treatment allocation, with the scope of maintain the blind within most members of the trial team, particularly the data managers involved in the data checking and cleaning for the follow-up phase of the trial.

5. Breaking the blind at the end of the study (12-month time point)

5.1. The Chief PI determines the appropriate method for informing individually all participants of their allocation group.

Document History		
**Previous Version**	**Author**	**Reason for change**
1.0	Joyce Chan<br>Veronica Abruzzo	SOP have been updated to:<br><br>Clarify that requestor to break the code need to be the site PI<br><br>Explain that the site PI/delegate needs to document the code breaking on REDCap AE SE form and site TMF.<br><br>Inform site PI/delegate needs to complete a protocol deviation from and if participant withdraw from the trial this needs to be documented in REDCap participant status form.<br><br>Include information that MCRI (sponsor) will notify RCH Ethics Committee and Data Safety Monitoring Committee

C09 BRACE Global SOP_Code breaking procedures_v2.0_07Oct2020 (CL)

Final Audit Report 2020-11-16

Created:	2020-11-10
By:	Thilanka Morawakage (thilanka.morawakage@mcri.edu.au)
Status:	Signed
Transaction ID:	CBJCHBCAABAA-hf86o8lp4ZllTR1xeLRkr5thOxt64o2

"C09 BRACE Global SOP_Code breaking procedures_v2.0_07Oct2020 (CL)" History

- Document created by Thilanka Morawakage (thilanka.morawakage@mcri.edu.au)
  2020-11-10 - 1:20:26 AM GMT- IP address: 101.181.38.128
- Document emailed to Francesca Orsini (francesca.orsini@mcri.edu.au) for signature
  2020-11-10 - 1:22:03 AM GMT
- Email viewed by Francesca Orsini (francesca.orsini@mcri.edu.au)
  2020-11-16 - 3:27:43 AM GMT- IP address: 45.141.142.8
- Document e-signed by Francesca Orsini (francesca.orsini@mcri.edu.au)
  Signature Date: 2020-11-16 - 5:45:49 AM GMT - Time Source: server- IP address: 203.16.41.5
- Document emailed to Laure Pittet (laure.pittet@mcri.edu.au) for signature
  2020-11-16 - 5:45:51 AM GMT
- Email viewed by Laure Pittet (laure.pittet@mcri.edu.au)
  2020-11-16 - 6:05:20 AM GMT- IP address: 45.141.142.60
- Document e-signed by Laure Pittet (laure.pittet@mcri.edu.au)
  Signature Date: 2020-11-16 - 6:05:41 AM GMT - Time Source: server- IP address: 203.16.41.5
- Document emailed to Nigel Curtis (nigel.curtis@rch.org.au) for signature
  2020-11-16 - 6:05:43 AM GMT
- Email viewed by Nigel Curtis (nigel.curtis@rch.org.au)
  2020-11-16 - 7:06:13 AM GMT- IP address: 144.136.217.78
- Document e-signed by Nigel Curtis (nigel.curtis@rch.org.au)
  Signature Date: 2020-11-16 - 7:06:44 AM GMT - Time Source: server- IP address: 144.136.217.78

Adobe Sign

Agreement completed.

2020-11-16 - 7:06:44 AM GMT

Adobe Sign

murdoch children's research institute

BRACE DATA AND SAFETY MONITORING COMMITTEE (DSMC) CHARTER

Protocol Title:	BCG vaccination to Reduce the impact of COVID-19 in healthcare workers following Coronavirus Exposure (BRACE) Trial.
Protocol No:	HREC/protocol no: 62586
Trial Registration No.	NCT04327206
Protocol Version & Date this Charter is based on:	Version 7.2, 8th May 2020
Chief Principal Investigator:	A/Prof Nigel Curtis<br>Nigel.Curtis@rch.org.au
Study Sponsor:	Murdoch Children's Research Institute (MCRI)

REVISION HISTORY

Version No.	Date	Summary of Changes
1.0	21 May 2020	Initial version

Pittet LF, et al. BMJ Open 2023; 11:e052101. doi: 10.1136/bmjopen-2021-052101

murdoch children's research institute

AGREEMENT

The members of the DSMC must sign the charter to indicate their approval of the content and agreement to adhere to the terms of this charter.

Name and Title	DSMC Role<br>*(e.g. chair, clinical expert, biostatistician, independent statistician, ex officio DSMC member)*	Signature	Date
Prof Colin Powell			
Prof Julie Simpson			
Prof Adam Finn			
Dr Kaushala Naiwala Pathirannehelage			

Pittet LF, *et al. BMJ Open* 2023; 11:e052101. doi: 10.1136/bmjopen-2021-052101

TABLE OF CONTENTS

REVISION HISTORY ... 1
1. INTRODUCTION ... 4
2. DSMC MEMBERSHIP ... 4
2.1 Exclusion of Conflicts of Interest ... 5
2.2 Resignation/Termination of DSMC Member and Replacement ... 5
3. RESPONSIBILITIES OF THE DSMC ... 5
3.1 Stewardship of the trial ... 5
3.2 Safety Monitoring ... 6
3.3 Monitoring of Efficacy Data - Interim Analyses ... 7
4. FREQUENCY AND FORMAT OF MEETINGS ... 7
4.1 Initial Meeting ... 8
4.2 First Review Meeting ... 8
4.3 Subsequent Review Meetings ... 8
4.4 Ad Hoc Meetings ... 10
5. CONDUCT OF MEETINGS ... 10
5.1 Open Session ... 10
5.2 Closed Session ... 10
5.3 Meeting Attendance and Quorum ... 10
5.4 Meeting Deliberations ... 11
5.5 DSMC Recommendations ... 11
5.6 Meeting Minutes ... 12
5.7 Trial Publications ... 12
6. STATISTICAL MONITORING AND REPORTS ... 12
6.1 Data Analysis and DSMC Reporting ... 12
6.1.1 Open and Closed Reports ... 12
7. CONFIDENTIALITY ... 14
8. COMMUNICATIONS ... 14
9. LIABILITY STATUS OF THE DSMC ... 14
10. KEY TERMS ... 15

Pittet LF, *et al. BMJ Open* 2023; 11:e052101. doi: 10.1136/bmjopen-2021-052101

1. INTRODUCTION

This charter is for the Data and Safety Monitoring Committee (DSMC) for the clinical trial entitled: BCG vaccination to reduce the impact of COVID-19 in healthcare workers (BRACE) Trial.

This charter defines the responsibilities of the DSMC, its membership, and the purpose and timing of its meetings. The Charter also provides the procedures for ensuring confidentiality and proper communication, the statistical monitoring procedures to be implemented by the DSMC, and an outline of the content of the reports that will be provided to the DSMC.

See Section 10 for a list of definitions of the terms and abbreviations used in this charter.

2. DSMC MEMBERSHIP

The DSMC consists of the following independent members who collectively have experience in the clinical area of interest, biostatistics and randomised clinical trials. A quorum will require at least 3 members.

The DSMC will consist of:
Voting members

DSMC Role	Name and Title	Affiliation / Institution	Email	Summary of expertise
DSMC Chair	Prof Colin Powell	Hon Prof of Child Health, General & Emergency Paediatrics, Cardiff University	powellc7@cardiff.ac.uk	Expertise in clinical trials, has previously chaired DSMC
DSMC Biostatistician	Prof Julie Simpson	Head of Biostatistics, Melbourne School of Population and Global Health, The University of Melbourne	julieas@unimelb.edu.au	Expertise in biostatistics
DSMC Clinical Expert	Prof Adam Finn	University of Bristol, NIHR Clinical Research Network, WHO	adam.finn@bristol.ac.uk	Expertise in infectious diseases, vaccination trials and vaccine off-target effects; previous experience in vaccine trial DSMBs

Non-voting members:

The Independent Statistician preparing confidential reports for the Closed Session will be Dr Kaushala Naiwala Pathirannehelage, Clinical Epidemiology and Biostatistics Unit, Murdoch Children's Research Institute.

Pittet LF, *et al. BMJ Open* 2023; 11:e052101. doi: 10.1136/bmjopen-2021-052101

murdoch children's research institute

Trial investigators will not be members of the DSMC.

2.1 Exclusion of Conflicts of Interest

The DSMC membership will be restricted to individuals free of any conflicts of interest. Even the appearance of conflict of interest among DSMC members must be avoided. Any DSMC member who has, or develops, a significant conflict of interest should resign from the DSMC.

The DSMC members must disclose conflicts of interest to fellow members. Declaration of a conflict of interest is an ongoing process; it will be completed at the time of joining the DSMC and prior to each DSMC meeting and will be recorded in the meeting minutes.

Conflicts of interest can include:

- Stock ownership in any commercial companies involved
- Stock transaction in any commercial company involved (if previously holding stock)
- Consulting arrangements with the sponsor
- Frequent speaking engagements on behalf of the intervention
- Career tied up in a product or technique assessed by the trial
- Hands-on participation in the trial
- Involvement in the running of the trial
- Emotional involvement in the trial
- Intellectual conflict (e.g. strong prior belief in the trial's experimental arm)
- Involvement in regulatory issues relevant to the trial procedures
- Investment (financial or intellectual) in competing products
- Involvement in the publication

The DSMC will function independently of all other individuals and bodies associated with the conduct of the trial.

2.2 Resignation/Termination of DSMC Member and Replacement

DSMC membership is for the duration of the clinical trial. If any members leave the DSMC during the course of the trial, the Chief Principal Investigator will promptly appoint their replacement with agreement from the remaining members of the DSMC. Further appointments may be made to the DSMC if members believe additional expertise is required. DSMC members can decide to terminate the membership of a DSMC member based on a simple vote in case of non-performance or other significant reasons as determined by a majority of the DSMC.

3. RESPONSIBILITIES OF THE DSMC

3.1 Stewardship of the trial

The DSMC is responsible for the stewardship of the trial over all participating sites or institutions. The stewardship includes review of participant recruitment, accrual, retention, and withdrawal. It further involves

oversight of participant management, adherence to protocol-specified regimens, and procedures for data management and quality control.

The DSMC will be responsible for safeguarding the interests of trial participants by assessing the safety of the interventions during the trial, and the general progress of the trial.

Specifically, the role of the DSMC will be to:

- Monitor and review participant safety in the trial (including evidence for treatment harm, e.g. toxicity data, safety events)
- Review participant recruitment, accrual, retention, trial withdrawal, serious breaches, and protocol deviations
- Monitor efficacy based on pre-planned interim data analyses (only applicable for one of the three planned interim analyses)

This responsibility will be exercised by providing recommendations about continuing, modifying or stopping the trial, including recommendation to publish safety data. To contribute to enhancing the integrity of the trial, the DSMC may also formulate recommendations relating to the selection/recruitment/retention of participants, participant management, and the procedures for data management and quality control.

The DSMC will be advisory to the Chief Principal Investigator and through him to the Trial Steering Committee.

The Chief Principal Investigator holds ultimate responsibility for decisions regarding the trial.

3.2 Safety Monitoring

The DSMC is responsible for safeguarding the interests of trial participants by assessing the safety of the interventions during the trial.
At least one DSMC member will be an expert in the potential safety outcomes of the trial. If important safety items are not considered in the reporting of safety data, the DSMC may request to change or add items to be included.

The study has three mechanisms for monitoring safety:

a) During recruitment of the study, the safety officer (who is the chair of the DSMC) will review all adverse events (including deaths and admissions to ICU) on a weekly basis in a semi blinded fashion (group A and group B) and report any concerns to the Chief Principal Investigator and/or to the DSMC members to act promptly if there are concerns. The safety officer has complete discretion with what to do with these safety data. The safety officer may choose to be unblinded if it helps any decision. This role will cease once recruitment is complete

b) A formal DSMC meeting at 3 months and 9 months.

c) An interim analysis after 100 cases of severe COVID-19 disease, whenever that occurs, but likely after recruitment ceases, and most probably after at least 3 months from the beginning of recruitment. This is primarily designed to identify efficacy but may also identify harm. Note that the definition of severe COVID-19 disease is as per defined in the protocol and thus includes being significantly unwell at home but not

Pittet LF, *et al. BMJ Open* 2023; 11:e052101. doi: 10.1136/bmjopen-2021-052101

murdoch children's research institute

hospitalised. If good evidence of efficacy is found early then this might have global health implications. If harm is found then this would also be important to know as it may impact on other BCG trials around the world.

3.3 Monitoring of Efficacy Data - Interim Analyses

The DSMC are also responsible for assessment of the efficacy of the interventions during the course of the trial i.e. interim analyses of efficacy endpoints. The current approved study protocol pre-specifies an interim analysis of the efficacy data once there have been 100 cases of severe COVID-19 disease.

This interim analysis will primarily be on a comparison of the number of cases of severe COVID-19 disease (primary outcome (2)) between the BGG group (irrespective of whether the participants received flu vaccine at randomisation) and the control group (irrespective of whether the participants received flu vaccine or placebo at randomisation), although data will also be presented on COVID-19 disease (primary outcome (1)) and also separately for those recruited prior to and post the introduction of the placebo to provide the DSMB with a complete picture. The timing of the interim analysis will be event driven, and will be conducted using a time-to-event analysis, censoring participants who have not had the event at the time of their last follow-up. This data will be used to provide Kaplan-Meier estimates of the survival curve in the BCG and control groups, which will be used to estimate the proportion with severe COVID-19 disease at 6 months. These proportions will be used to compare the two groups.
We have allocated alpha=0.005 to this interim analysis using the conservative approach of splitting the alpha allocated to primary outcome (2) between the interim and final analysis. Under the original sample size calculation, with 1668 per group and an incidence of 4% in severe COVID-19 disease at 6 months in the control group and 2% in the intervention group, this would equate to 67 + 33 = 100 cases in total. We therefore plan to conduct a formal interim analysis of severe COVID-19 disease once there have been 100 cases of severe COVID-19 disease. This interim analysis of the severe COVID-19 disease will be performed on the all the participants randomised up to the interim analysis time point, comparing all the participants who were randomised to BCG (irrespective of whether they received flu vaccine at randomisation) and those randomised to control (irrespective of whether they received flu vaccine or placebo at randomisation). As additional information, the DSMB will also be given information on which participants belong to the first phase of the study.

With 100 events, we will have 72% power to detect a risk ratio of 0.5 in the incidence of severe COVID-19 disease at 6 months at the interim analysis based on a two-sided test of alpha=0.005, which is a reasonable power for this interim analysis. **The stopping rule for the interim analysis will therefore be p<0.005**.

Given the dynamic nature of research in this field, the DSMB will be advised that this rule be used as a guideline rather than a formal rule, and should be interpreted in the context of external information and information on the efficacy of BCG vaccination on the incidence of COVID-19 disease (primary outcome (1)) which will also be presented in the DSMB report using the same methodology.

4. FREQUENCY AND FORMAT OF MEETINGS

A total of three DSMB meetings is planned, two of which at the fixed time point of 3 and 9 months after the commencement of recruitment, and one as soon as there have been 100 cases of severe COVID-19 disease.

Pittet LF, et al. BMJ Open 2023; 11:e052101. doi: 10.1136/bmjopen-2021-052101

murdoch children's research institute

4.1 Initial Meeting

The initial meeting of the DSMC will review the role and functioning of the DSMC, discuss the format and content of the DSMC reports and review scientific and ethical issues relating to the design and conduct of the trial.

4.2 First Review Meeting

<u>3 months DSMB meeting</u>

The first DSMC meeting will occur at 3 months post initial recruitment. At this meeting, the following will be reviewed:

- Data related to common reactions to BCG vaccination within the first 2 weeks post vaccination
- Data related to uncommon and rare side effects of BCG vaccination within the first 2 weeks post vaccination
- Data relating to trial conduct, including participants disposition and protocol deviations
- Data completeness
- Data related ICU to admission, and death

Note that at 3 months, recruitment may have ceased or it may be ongoing. It is unlikely that within 3 months we will have enough severe cases to trigger the interim efficacy analysis mentioned in section 3.3. However, at 3-months, the DSMC may be alarmed by a high rate of adverse events, ICU admissions or deaths in either group for any reasons. If this DSMC meeting occurs during while recruitment is still ongoing, the DSMC may suggest to stop recruitment. If meeting after recruitment has finished, the DSMC may instruct the investigators to unblind the study and publish the deaths and ICU admission rates as this may have an impact on other BCG studies if there is harm or starting treatment if there is benefit. The DSMB do not have a formal stopping rule for this decision at 3 months. They may request unblinded data to inform their decision.

4.3 Subsequent Review Meetings

<u>9 months DSMB meeting</u>

A subsequent fixed term DSMC meeting will occur at 9 months post initial recruitment. At this meeting, the following will be reviewed:

- Data related to common reactions to BCG vaccination within the first 2 weeks post vaccination
- Data related to uncommon and rare side effects of BCG vaccination within the first 2 weeks post vaccination
- Data related to adverse events and adverse reactions (non-serious and serious) within 3 months post vaccination
- Data relating to trial conduct, including participants disposition and protocol deviations
- Data completeness
- Data related ICU to admission, and death

Pittet LF, et al. BMJ Open 2023; 11:e052101. doi: 10.1136/bmjopen-2021-052101

murdoch children's research institute

Note that at 9 months, recruitment will have ceased. It is likely that within 9 months since the beginning of recruitment, there will be enough severe cases to trigger the interim efficacy analysis mentioned in section 3.3. If not, at 9-months, the DSMC may be alarmed by a high rate of adverse events, ICU admissions or deaths in either group for any reasons. In this case the DSMC may instruct the investigators to unblind the study and publish the deaths and ICU admission rates as this may have an impact on other BCG studies if there is harm or starting treatment if there is benefit. The DSMB do not have a formal stopping rule for this decision at 9 months. They may request unblinded data to inform their decision.

DSMB meeting at 100 cases of Severe COVID-19 disease

As anticipated in section 3.3 of this Charter, another DSMC meeting will occur once there have been 100 cases of severe COVID-19 disease. At this meeting, the following will be reviewed:

- Data related to common reactions to BCG vaccination within the first 2 weeks post vaccination (if recruitment is still ongoing at this time point)
- Data related to uncommon and rare side effects of BCG vaccination within the first 2 weeks post vaccination (if recruitment is still ongoing at this time point)
- Data related to adverse events and adverse reactions (non-serious and serious) within 3 months post vaccination
- Data relating to trial conduct, including participants disposition and protocol deviations
- Data completeness
- Data related to hospitalization, admission to ICU and death
- Data related to cases of severe COVID-19 disease – primary outcome (2) *
- Data related to cases of COVID-19 disease – primary outcome (1) [#]

* As stated in section 3.3 of this Charter, this DSMC will incorporate an interim analysis of efficacy. Specifically, it will primarily be on a comparison of the number of cases of severe COVID-19 disease (primary outcome (2)) between the BGG group (irrespective of whether the participants received flu vaccine at randomisation) and the control group (irrespective of whether the participants received flu vaccine or placebo at randomisation).
This analysis will be conducted using a time-to-event analysis, censoring participants who have not had the event at the time of their last follow-up. This data will be used to provide Kaplan-Meier estimates of the survival curve in the BCG and control groups, which will be used to estimate the proportion with severe COVID-19 disease at 6 months. These proportions will be used to compare the two groups.
This interim analysis of the severe COVID-19 disease will be performed on the all the participants randomised up to the interim analysis time point, comparing all the participants who were randomised to BCG (irrespective of whether they received flu vaccine at randomisation) and those randomised to control (irrespective of whether they received flu vaccine or placebo at randomisation). As additional information, the DSMB will also be given information on which participants belong to the first phase of the study.
With 100 events, we will have 72% power to detect a risk ratio of 0.5 in the incidence of severe COVID-19 disease at 6 months at the interim analysis based on a two-sided test of alpha=0.005, which is a reasonable power for this interim analysis. The stopping rule for the interim analysis will therefore be p<0.005.
Given the dynamic nature of research in this field, the DSMB will be advised that this rule be used as a guideline rather than a formal rule, and should be interpreted in the context of external information and information on the efficacy of BCG vaccination on the incidence of COVID-19 disease (primary outcome (1)) which will also be presented in the DSMB report using the same methodology.

Pittet LF, *et al. BMJ Open* 2023; 11:e052101. doi: 10.1136/bmjopen-2021-052101

[#] Data will also be presented on COVID-19 disease (primary outcome (1)) separately for those recruited prior to and post the introduction of the placebo to provide the DSMB with a complete picture.

4.4 Ad Hoc Meetings

Additional *ad hoc* meetings of the DSMC may be scheduled if requested by either the Chief Principal Investigator, the Trial Steering Committee or the DSMC.

5. CONDUCT OF MEETINGS

Meetings will consist of an open session and a closed session.

5.1 Open Session

Members of the BRACE Executive Committee, which includes the Chief Principal Investigator, will meet with the DSMC and the independent statistician who prepared the DSMC report at the commencement of each meeting. This "*Open Session"* provides the DSMC an opportunity to query the Executive Committee members about issues that have arisen during the review of the data. Once the DSMC members are satisfied that all their queries have been addressed, the Trial Steering Committee members will then leave the meeting to enable the confidential *Closed Session* of the DSMC to commence. The Chief Principal Investigator will remain available to return, if required, to assist with any questions.

5.2 Closed Session

The independent statistician who prepared the DSMC report will remain for the first part of the closed session in order to take the DSMC through the report and answer questions if required. The independent statistician will then leave the closed session. The remainder of the closed session will involve only DSMC members to allow discussion of confidential data from the clinical trial.

5.3 Meeting Attendance and Quorum

The minimum number of members in attendance for the DSMC to be quorate for decision-making is 3.

If the report is circulated before the meeting, DSMC members who will not be able to attend the meeting may pass comments to the DSMC Chair for consideration during the discussions.

If a member does not attend a meeting, it should be ensured that the member is available for the next meeting. If a member does not attend a second meeting, they should be asked if they wish to remain part of the DSMC. If a member des not attend a third meeting, they should be replaced.

murdoch children's research institute

5.4 Meeting Deliberations

The Chair will facilitate and summarise discussions and will encourage consensus. Following its review of the data, the DSMC will reach consensus on its list of recommendations. Consensus will be determined through formal voting.

5.5 DSMC Recommendations

The recommendations provided by the DSMC may include:

1. Continuing the trial unchanged
2. Continuing the trial with modifications; or
3. Terminating the trial.
4. Publishing safety data

The DSMC may also make recommendations about other aspects of the trial such as the recruitment of participants and the conduct of the trial. All recommendations will be sent to the Chief Principal Investigator promptly, within 2 weeks, and through the Chief Principal Investigator to the Trial Steering Committee. The Trial Steering Committee will advise on whether to continue or terminate the trial, and whether amendments to the protocol or changes in trial conduct are required based on the DSMC recommendations.

In the event that the DSMC recommends the early termination of the trial, the final decision to stop the trial early or modify the trial protocol will be made by the Chief Principal Investigator following advice from the Trial Steering Committee. If this situation arises at any time, the decision of the Chief Principal Investigator will be discussed with the DSMC immediately.

The DSMC will be advisory to the Chief Principal Investigator and through him/her to the Trial Steering Committee. The Chief Principal Investigator holds ultimate responsibility for decisions regarding the trial.

The Chief Principal Investigator will be responsible for promptly presenting the recommendations of the DSMC to the Trial Steering Committee for ready review. The Trial Steering Committee will advise on whether to continue or terminate the trial, and whether amendments to the protocol or changes in trial conduct are required based on the DSMC recommendations.

Response to the DSMC's recommendations:

- If the Chief Principal Investigator /TSC do not agree with the DSMC recommendations, a memo justifying the reasons for not complying with the recommendations of the DSMC will be promptly forwarded to the DSMC and to the Sponsor, within 2 weeks,
- If the DSMC is not satisfied with the Chief Principal Investigator /TSC response to their recommendations, the DSMC will promptly notify the Sponsor, within 2 weeks.

Note that in the event that the Chief Principal Investigator /TSC wishes to remove one or more DSMC members, a memo justifying the reasons for this will be promptly forwarded to the DSMC and to the HREC, i.e. within 2 weeks.

Pittet LF, *et al. BMJ Open* 2023; 11:e052101. doi: 10.1136/bmjopen-2021-052101

murdoch children's research institute

5.6 Meeting Minutes

The DSMC will have minutes taken for both the Open and Closed meetings. Meeting minutes should be signed by the DSMC Chair and distributed as soon as possible after the DSMC meeting. The Trial Steering Committee will provide staff to assist with minute-taking. The person taking minutes for the closed meeting will be independent of the trial team and will ensure the minutes of the closed meeting remain confidential until the completion of the trial.

5.7 Trial Publications

The DSMC may be sent copies of accepted papers for their information.

DSMC members should be named and their affiliations listed in the main report/publication. A brief summary of the timings and conclusions of the DSMC meetings should be included in the body of the main trial paper.

6. STATISTICAL MONITORING AND REPORTS

6.1 Data Analysis and DSMC Reporting

A statistician independent of the sponsor will perform the unblinded interim analysis.

6.1.1 Open and Closed Reports

The independent statistician will undertake the data analysis and the creation of the DSMC reports. The statistician preparing the information for the DSMC will prepare two reports, an "open" and a "closed" report (see sections 6.1.1 and 6.1.2 below). Both the open report and the confidential closed report will be sent to the DSMC members for review 7 days prior to the scheduled meeting.

The open report will also be circulated to the Trial Steering Committee which will meet shortly after the DSMC to discuss any recommendations made by the DSMC along with any other trial related issues.

6.1.1.1 Open Reports

Open reports will contain the following information:

- Trial number and title.
- Brief summary of the trial design and progress.
- Details of any protocol amendments since the previous report
- Status of accrual (actual vs target recruitment)
  - If accrual is slower than expected include a plan for increasing enrollment.
  - Report all sites by name, target recruitment and current recruitment
- Summary of patients disposition (including data completeness)
- Summary of baseline characteristics
- Summary of common reactions to vaccination within the first 2 weeks post vaccination (if recruitment is still ongoing at this time point)
- Summary of uncommon and rare side effects of vaccination within the first 2 weeks post vaccination (if recruitment is still ongoing at this time point)

murdoch children's research institute

- Summary of adverse events and adverse reactions (non-serious and serious) within 3 months post vaccination [not applicable to DSMC at 3 months]
- Summary of ICU admission and death
- Summary of hospitalization [only for DSMC at 100 severe cases of COVID-19 disease]
- Summary of cases of severe COVID-19 disease – primary outcome (2)
- Summary of cases of COVID-19 disease – primary outcome (1)
- Summary of protocol deviations
- Details of serious breaches

Data in this report will be presented across all participants with NO reference to treatment group.

6.1.1.2 Closed Reports

Closed reports will contain the following information:

- Trial number and title.
- Brief summary of the trial design and progress.
- Details of any protocol amendments since the previous report
- Status of accrual (actual vs target recruitment)
- If accrual is slower than expected include a plan for increasing enrollment.
- Report all sites by name, target recruitment and current recruitment
- Summary of patients disposition (including data completeness)
- Summary of baseline characteristics
- Summary of common reactions to vaccination within the first 2 weeks post vaccination (if recruitment is still ongoing at this time point)
- Summary of uncommon and rare side effects of vaccination within the first 2 weeks post vaccination (if recruitment is still ongoing at this time point)
- Summary of adverse events and adverse reactions (non-serious and serious) within 3 months post vaccination [not applicable to DSMC at 3 months]
- Summary of ICU admission and death
- Summary of hospitalization [only for DSMC at 100 severe cases of COVID-19 disease]
- Summary and statistical comparison of cases of severe COVID-19 disease by treatment group – primary outcome (2)
- Summary of cases of COVID-19 disease – primary outcome (1)
- Summary of protocol deviations
- Details of serious breaches

The format of these reports will be determined by the DSMC in consultation with the statistician preparing the report. Information will be presented by pseudo-labelled treatment group (e.g. "A" and "B"). In some circumstances, unintended unblinding may occur if certain reported parameter values are expected to be associated with the interventions, such as common reactions to vaccination. In such circumstances, the need for presenting data by treatment group should be carefully considered among members of the DSMC.

Of note, un-blinding can occur in all three scenarios:

- If requested by the DSMC at meetings 3 and 9 months into the study
- During the interim analysis after 100 severe cases are reached if there are signs of efficacy or harm "close" to the stopping rule.

Pittet LF, et al. BMJ Open 2023; 11:e052101. doi: 10.1136/bmjopen-2021-052101

murdoch children's research institute

- If requested by the independent safety monitor during the weekly reporting period in the recruitment phase

Note the safety monitor or the DSMC may request unblinding to inform a final decision as to whether or not to "stop" the study and disseminate results. The decisions as to whether or not to request unblinding are inevitably subjective and up to the safety monitor or DSMB. This decision would be driven by the magnitude of any increased harm between groups and the current external evidence for likely harm. For example, if there is increasing external evidence that BCG may be harmful then a smaller difference in magnitude may trigger the decision for unblinding.

The key to identify the treatment regimens may be supplied by the statistician if requested by the DSMC.

Additional information may be presented in subsequent reports if specifically requested by the DSMC.

7. CONFIDENTIALITY

All DSMC reports will remain confidential until the end of the trial. Details of DSMC discussions and draft reports will remain confidential until formally delivered to the Chief Principal Investigator and through him/her to the TSC.

After each meeting, the DSMC members should store the papers safety after each meeting so that they may check the next report against them. After the trial is reported the DSMC members should destroy all interim reports.

8. COMMUNICATIONS

At any time during the trial, regulatory authorities, the Human Research Ethics Committee, the Trial Steering Committee or any other body or individual involved with the conduct of the trial may seek the advice of the DSMC about any concern that they may have about the conduct, outcome or continuation of the trial. Any such requests should be forwarded in writing to the DSMC Chairperson at the address provided above.

If any suspected unexpected serious adverse events occur, which are thought to relate to the experimental treatment, the Chair of the DSMC will be notified within 72 hours. The Chair will then decide whether an additional meeting of the DSMC should be held.

9. LIABILITY STATUS OF THE DSMC

As the DSMC is an advisory body alone, its members are not liable for damage, harm, morbidity or mortality to recruited patients.

Pittet LF, *et al. BMJ Open* 2023; 11:e052101. doi: 10.1136/bmjopen-2021-052101

murdoch children's research institute

10. KEY TERMS

DSMC: A Data Safety Monitoring Committee is an independent data-monitoring group that may be established by those responsible for trial conduct to monitor the progress of a clinical trial with focus on potentially arising safety issues.

SERIOUS BREACH: A breach of Good Clinical Practice or the protocol that is likely to affect to a significant degree: the safety or rights of a trial participant, or the reliability and robustness of the data generated in the clinical trial. Note: this guidance's definition of serious breach differs from the definition in the Australian Code for the Responsible Conduct of Research and is about deviations from the requirements of Good Clinical Practice or the clinical trials protocol.

TSC: Trials may or may not have a Trial Steering Committee (TSC). The aim of this committee is to provide independent oversight for trials, including responsibility for the scientific integrity of the protocol and the assessment of study quality and conduct. The TSC usually includes the Chief Principal Investigator, some principal investigators from study sites and possibly other key members of the TMG. The TSC also often includes external members who are independent of the trial conduct and may have an independent chair. Such a committee is often only used for trials that are large, complex or potentially controversial, or where there is a need to include a range of key stakeholders in the oversight of the trial.

Pittet LF, *et al. BMJ Open* 2023; 11:e052101. doi: 10.1136/bmjopen-2021-052101

BRACE Steering Committee

Steering Committee	BRACE team members
Ann Ginsberg<br>Kanta Subbarao<br>Kim Mulholland<br>Nigel Curtis<br>Peter Richmond<br>Andrew Steer	Amber Sastry<br>Andrew Davidson<br>Emma Watts<br>Francesca Orsini<br>Joyce Chan<br>Katherine Lee<br>Laure Pittet<br>Nicole Messina<br>Tenaya Jamieson

Steering Committee Business

- **Conflicts of interest**
  - Ann – BRACE program officer for BMGF
  - Kim – member of Safety Monitoring Committee of Novavax trial
  - Kanta – planning to be involved in looking at fluvax responses in subgroup with one of the trial investigators
  - Peter – investigator on BRACE, holds another grant from BMGF
  - Nigel – BRACE chief investigator
- **Trial Steering Committee (TSC) scope**
  - To mentor the Trial Leadership Team, as required
  - To provide strategic advice about the direction of the trial
  - To provide impartial and informed advice to ensure the rigor of the trial
  - To provide a high-level consideration of budget, recruitment strategy, data quality, safety data, and resolve specific strategic issues
  - To ratify difficult decisions about data and sample access
  - To ratify decisions about authorship
- **Role of TSC vs DSMB**
  - DSMB reports to CPI (Nigel) according to current DSMB Charter
  - TSC to be copied into 3m/9m DSMB reports
  - TSC to be made aware of interim analysis details/full Protocol
    - When 100 severe COVID-19 cases are reached
    - Decision made in conjunction with biostatisticians from BMGF
- **TSC composition**
  - Content experts – Kanta, Kim, Nigel
  - Trials – Andrew D, Peter
  - Funding – Ann, Peter
  - Sponsor (MCRI) – Andrew S
- Clarified that TSC is an **advisory**, not decision-making, committee
- **Meeting schedule**
  - Every 3 months during recruitment period, given short duration of BRACE
- **Nominate a new independent Steering Committee Chair**
  - Current chair: Andrew Steer

        - MCRI has no vested interest in BCG vaccine, so no major concerns with having an MCRI staff member as TSC Chair
    - Discussed need for independent (non-MCRI) Chair – agreed no immediate need
- **Objective of Meeting**
    - To seek an independent and impartial review of the BRACE Trial plans

1.

murdoch children's research institute | BRACE trial
BCG vaccination to reduce the impact of COVID-19 in healthcare workers

1.1.1

BRACE TRIAL BRACELET COMMITTEE TERMS OF REFERENCE

OVERVIEW

The BRACE Trial BRACELET Committee manages the operational aspects of the trial and ensures it operates within the local and international required standards. The primary responsibility for the trial and its day-to-day management remains the responsibility of the Chief Principal investigator (CPI). The CPI on behalf of the BRACELET Committee raises issues to the PI Committee. The CPI also seeks advice and provides information to the Trial Steering Committee (TSC).

The role of the BRACELET meeting is to:

- Ensure that all sites adhere to conducting the trial in strict compliance with the protocol, SOPs, guidelines and applicable ethics and regulatory bodies.
- Focus discussion on progress and operational needs and actions required to meet study milestones (e.g. recruitment) and ongoing participant follow up, to maximise the likelihood of completion within the agreed time period and collection of high-quality data.
- Discuss funding status, budgets and opportunities.
- Discuss, where relevant, any new barriers or opportunities that arise which may have an impact on the successful completion of the trial.

The BRACELET Committee includes:

- a Chairperson
- CPI
- Senior management trial staff (MCRI)
- Statistician(s) (MCRI)

The BRACELET Committee should meet weekly, with frequency of meetings amended as agreed; Minutes of all meetings will be circulated to the BRACELET Committee and senior central trial data managers and kept on file. Post-trial, all documents should be archived in the Trial Master File with other essential documents.

murdoch children's research institute | BRACE trial
BCG vaccination to reduce the impact of COVID-19 in healthcare workers

BRACE TRIAL BRACELET COMMITTEE CHARTER

Protocol Title:	BCG vaccination to reduce the impact of COVID-19 in healthcare workers (BRACE) Trial
Protocol #:	Version 10.1, 10 December 2020
Protocol Version & Date this Charter is based on:	Version 10.1, 10 December 2020
Study Sponsor:	
Sponsor-Investigator (clinical trials only):	Murdoch Children's Research Institute

REVISION HISTORY

Version No.	Date	Summary of Changes
1.0	27/01/21	Initial version

Pittet LF, *et al. BMJ Open* 2023; 11:e052101. doi: 10.1136/bmjopen-2021-052101

TABLE OF CONTENTS

REVISION HISTORY ... 2
1. CORE MEMBERSHIP ... 4
  1.1 Chair of the BRACELET Committee ... 4
2. RESPONSIBILITIES OF THE BRACELET Committee ... 4
3. DATA ... 5
4. FREQUENCY AND FORMAT OF MEETINGS ... 5
  4.1 Meeting Frequency ... 5
  4.2 *Ad Hoc* Meetings ... 5
  4.3 Meeting Attendance and Quorum ... 5
  4.4 Meeting Deliberations ... 5

Pittet LF, *et al. BMJ Open* 2023; 11:e052101. doi: 10.1136/bmjopen-2021-052101

2. CORE MEMBERSHIP

The BRACELET Committee membership consists of:

Name and Title	Affiliation / Institution	Email
Prof. Nigel Curtis	Murdoch Children's Research Institute	nigel.curtis@rch.org.au
Andrew Davidson	Melbourne Children's Trials Centre	andrew.davidson@rch.org.au
Susan Perlen	Murdoch Children's Research Institute	susan.perlen@mcri.edu.au
Nicole Messina	Murdoch Children's Research Institute	nicole.messina@mcri.edu.au
Laure Pittet	Murdoch Children's Research Institute	laure.pittett@mcri.edu.au
Tenaya Jamieson	Murdoch Children's Research Institute	tenaya.jamieson@mcri.edu.au
Jia Wei Teo	Murdoch Children's Research Institute	jiawei.teo@mcri.edu.au
Kaya Gardiner	Murdoch Children's Research Institute	kaya.gardiner@mcri.edu.au
Thilanka Morawakage	Murdoch Children's Research Institute	thilanka.morawakage@mcri.edu.au
Katherine Lee	Murdoch Children's Research Institute	katherine.lee@mcri.edu.au
Francesca Orsini	Murdoch Children's Research Institute	francesca.orsini@mcri.edu.au
Cecilia Moore	Murdoch Children's Research Institute	cecilia.moore@mcri.edu.au
Amanda Gwee	Royal Children's Hospital	amanda.gwee@rch.org.au
Kirsten Perrett	Royal Children's Hospital	kirsten.perrett@rch.org.au

2.1 Chair of the BRACELET Committee

The Chair of the meeting is Susan Perlen.

3. RESPONSIBILITIES OF THE BRACELET Committee

The BRACELET Committee provides operational oversight and ensures that the trial is conducted to the required standards. The Committee, through the CPI, can seek advice from the PI Committee and the Steering Committee; the primary responsibility for the trial and its day-to-day management remains the responsibility of the CPI.

The BRACELET Committee will have responsibility for monitoring trial progress and managing the operational requirements of the trial. These include:

1. Overseeing progress towards trial milestones (i.e. recruitment accruals, timelines etc.).
2. Reviewing adherence/compliance to the protocol and adherence/compliance to good clinical research practices in line with GCP requirements.
3. Discussing and managing operational requirements for ongoing conduct of the trial (For example, ethics, laboratory, communications, safety, monitoring, data management, risk, staffing).
4. Providing and discussing local and international site updates/issues.
5. Discussing discontinuation or extension of recruitment.
6. Discussing strategic or specific decisions such as scientific developments, rigour, and funding opportunities.
7. Considering any new external information relevant to the study.

4. DATA

The BRACELET Committee will be provided with updated documents containing the following data elements:

- Summary of progress to date, including site activation status, recruitment update, follow-up update, trigger episodes, missing COVID-19 tests etc.
- Summary of any requests reviewed by the Biosample and Data Use Committee.
- Summary of any upcoming manuscripts/abstracts.

5. FREQUENCY AND FORMAT OF MEETINGS

5.1 Meeting Frequency

The BRACELET Committee will meet every week via videoconference during the recruitment phase of the study. During the follow-up phase of the study, timing of meetings will be re-visited and held as agreed via videoconference.

5.2 *Ad Hoc* Meetings

Additional *ad hoc* BRACELET meetings may be scheduled if requested by the CPI.

5.3 Meeting Attendance and Quorum

The minimum number of members in attendance for the BRACELET Committee to be quorate for decision-making is seven members. If at any time the number of members is less than a quorum, the BRACELET Committee may meet only for discussion purposes.

5.4 Meeting Deliberations

The Chair will facilitate and summarise discussions and will encourage decision-making via consensus. Meetings will be minuted and any decisions made electronically will be recorded in the form of meeting minutes and distributed to members within 4 days of the meeting. All meeting agendas, meeting minutes generated, and other relevant documentation will be filed in the Trial Master File (TMF).

The discussions of the BRACELET Committee are confidential to its members.

Pittet LF, *et al. BMJ Open* 2023; 11:e052101. doi: 10.1136/bmjopen-2021-052101

murdoch children's research institute | BRACE trial
BCG vaccination to reduce the impact of COVID-19 in healthcare workers

BRACE trial – Authorship guidelines

Purpose: This guideline outlines a process for the decisions about authorship for the manuscripts, presentations and posters arising from the data or samples collected as part of the BRACE trial. The procedure has been established to ensure that eligibility for authorship is carefully considered and to enable MCRI to comply with international guidance, funding bodies' requirements and legal obligations.

Applicability: This guideline applies to all those involved in the BRACE trial including principal and sub-investigators, research coordinators, data managers and other staff. All principal investigators are directly responsible for ensuring that their research team are aware of the authorship guidelines where applicable.

Authorship eligibility: In all cases authorship will be determined within the ICMJE guidelines (see http://www.icmje.org/recommendations/browse/roles-and-responsibilities/defining-the-role-of-authors-and-contributors.html). Authors must have made substantive contributions to the design, conduct, interpretation and reporting of the trial.

Governance: Decisions in relation to authorship will follow the following process:

1. Depending on the category of the manuscript (as described below), the Chief Principal Investigator (CPI, Nigel Curtis)/Principal Investigator team will develop a core authorship list.
2. The authorship list will be reviewed, amended and/or approved by the BRACE trial Steering committee.
3. Disputes regarding authorship will be resolved between the Chair of the Steering committee and the Chief Principal Investigator committee

Process for publication of manuscripts: The following outlines the process for three separate categories of manuscripts from the BRACE trial:

<u>Category 1: Reports of the primary and secondary outcomes of the trial</u>

- The CPI (Nigel Curtis) will develop a core authorship list for each paper including at least one author from each participating study region. The number of named authors may vary but will not exceed the author limit stated in journal guidelines.
- The BRACE trial consortium will also be listed on the by-line. The BRACE trial consortium will include all those who meet criteria for authorship. The composition of the consortium may vary between papers and will be approved as per the governance process outlined above. In some circumstances those who contributed to BRACE may be acknowledged rather than named as authors or in the consortium.

<u>Category 2: Reports addressing one aspect of the trial but where the data are derived from the whole trial</u>

- Expressions of interest will be called for from the principal investigators to take the leads in preparing each manuscript.
- Based on the above, the principal investigator team will develop a core authorship list for each paper which will be approved as per the governance process above.
- The BRACE trial consortium will also be listed on the by-line as outlined above.

Pittet LF, *et al. BMJ Open* 2023; 11:e052101. doi: 10.1136/bmjopen-2021-052101

murdoch children's research institute | BRACE trial
BCG vaccination to reduce the impact of COVID-19 in healthcare workers

Category 3: Reports on data derived from sub-studies or reports of studies initiated outside of the trial but that use data or samples collected as part of the BRACE trial

- Authorship on sub-studies or reports of studies initiated outside the trial will follow the governance process outlined above.
- For site-specific publications, the lead author and majority of the authors will generally be from the site where the study was done with at least one co-author from each major study region that contributed to the data collection, sample collection or design.
- For studies that use data or samples collected as part of the BRACE trial, the lead and author list will be chosen by agreement between those involved in the study and the CPI. At least one co-author from each study site that contributed to the data or sample collection or design will be included.
- The listing of the BRACE consortium is optional, to be considered on a case-by-case basis.

Process for publication of abstracts:
It is recognised that authorship for posters may require fewer authors, however the approach to authorship will follow, as far as possible, the process outlined above as for manuscripts.

Future requests for data and samples:
It is recognised that future groups external to the current BRACE collaboration may request data and samples. These requests will be considered by the BRACE Biosample and Data Use Committee. This includes discussion about authorship and acknowledgement between the requestors, the MCRI as sponsor and data custodian, and the CPI of the BRACE trial team. A similar process will be in place for the BRACE data held in externally accessible platforms such as Vivli and BMGF except there Vivli and BMGF act as custodians rather than MCRI. Note that in all these circumstances the BRACE trial team will be consulted but cannot determine final authorship. Consultation will be via the CPI and the CPI may seek advice from other BRACE investigators as they see fit.

Pittet LF, *et al. BMJ Open* 2023; 11:e052101. doi: 10.1136/bmjopen-2021-052101

BRACE TRIAL CONSORTIUM GROUP LIST

VIC – MCRI, RCH

Name	Email	Role
**MCRI Central Team**		
**Nigel Curtis**	nigel.curtis@rch.org.au	BRACE Chief Principal Investigator
**Andrew Davidson**	Andrew.Davidson@rch.org.au	Investigator; Medical Director, MCTC
**Laure Pittet**	laure.pittet@mcri.edu.au	Investigator; BRACE Clinical Data Lead and Safety Lead
**Nicole Messina**	nicole.messina@mcri.edu.au	Investigator; BRACE Laboratory Lead
**Kirsten Perrett**	Kirsten.Perrett@rch.org.au	Investigator; Co-group Leader, Population Allergy; Clinician-Scientist Fellow, MCTC
**Amanda Gwee**	Amanda.Gwee@rch.org.au	Investigator; Team Leader, Infectious Group, MCRI
**Kaya Gardiner**	kaya.gardiner@mcri.edu.au	Investigator; former Program Manager
**Susan Perlen**	susan.perlen@mcri.edu.au	Current Program Manager
**Amber Sastry**	amber.sastry@mcri.edu.au	Former Program Manager
**Tenaya Jamieson**	tenaya.jamieson@mcri.edu.au	Senior Trial Coordinator
**Jia Wei Teo**	jiawei.teo@mcri.edu.au	Senior Trial Coordinator
**Thilanka Morawakage**	thilanka.morawakage@mcri.edu.au	Project Assistant
**Harriet Edmund**	harriet.edmund@mcri.edu.au	BRACE Communications Officer
**Biostatisticians**		
**Katherine Lee**	katherine.lee@mcri.edu.au	Senior Biostatistician
**Francesca Orsini**	francesca.orsini@mcri.edu.au	Biostatistician
**Cecilia Moore**	cecilia.moore@mcri.edu.au	Biostatistician
**Data Team**		
**Laure Pittet**	laure.pittet@mcri.edu.au	Investigator; Clinical Data Lead
**Ellie McDonald**	ellie.mcdonald@mcri.edu.au	Data Quality Senior Research Officer
**Richard Hall**	richard.hall@mcri.edu.au	Data Manager

BRACE trial Consortium Group List_29MAR2021

**Casey Goodall**	casey.goodall@mcri.edu.au	Senior Data Officer
**Grace Gell**	grace.gell@mcri.edu.au	Data Follow-up Coordinator
**Nathan La**	nathan.la@mcri.edu.au	Data Quality RA
**Ann Krastev**	ann.krastev@mcri.edu.au	Data Quality RA
**Ross Dunn**	ross.dunn@mcri.edu.au	REDCap RA
**Luke Stevens**	luke.stevens@mcri.edu.au	Data Management Coordinator
**Nick McPhate**	nick.mcphate@mcri.edu.au	Original/Current BRACE Team
**Jack Ren**	jack.ren@mcri.edu.au	Start-up Data, Data Engineer, Gen V, MCRI
**Laboratory Team**		
**Nicole Messina**	nicole.messina@mcri.edu.au	Investigator; BRACE Laboratory Lead
**Rhian Bonnici**	rhian.bonnici@mcri.edu.au	Site Laboratory Coordinator
**Susie Germano**	susie.germaNo@mcri.edu.au	Laboratory RA
**Rebecca McElroy**	rebecca.mcelroy@mcri.edu.au	Laboratory RA
**Laboratory Scientists**		
**Ashleigh Wee-Hee**	ashleigh.weehee@mcri.edu.au	Regular Lab
**Ahmed Alamrousi**	ahmed.alamrousi@mcri.edu.au	Regular Lab
**Belinda Ortika**	belinda.ortika@mcri.edu.au	Regular Lab
**Casey Pell**	casey.pell@mcri.edu.au	Regular Lab
**Leena Spry**	leena.spry@mcri.edu.au	Regular Lab
**Kristy Azzopardi**	kristy.azzopardi@mcri.edu.au	Start-up Lab
**Nadia Mazarakis**	nadia.mazarakis@mcri.edu.au	Start-up Lab
**Ryan Toh**	zheng.quantoh@mcri.edu.au	Start-up Lab
**Vicki Bennett-Wood**	vicki.bennettwood@mcri.edu.au	Start-up Lab
**Sunitha Velagapudi**	sunitha.velagapudi@mcri.edu.au	Start-up Lab
**Jeremy Anderson**	jeremy.anderson@mcri.edu.au	Start-up Lab
**Amanda Vlahos**	amanda.vlahos@mcri.edu.au	Start-up Lab
**Anna Czajko**	anna.czajko@mcri.edu.au	Start-up Lab

BRACE trial Consortium Group List_29MAR2021

**Biobanking**		
**Benjamin Ong**	benjamin.ong@mcri.edu.au	Lab Support; Head of Scientific Services
**Pedro Ramos**	pedro.ramos@mcri.edu.au	Biobanking Coordinator
**Anushka Karunanayake**	anushka.karunanayake@mcri.edu.au	Biobanking Staff
**Dinusha Gamage**	dinusha.gamage@mcri.edu.au	Biobanking Staff
**Enoshini Sooriyarachchi (Ushani)**	enoshini.sooriyarach@mcri.edu.au	Biobanking Staff
**Isabella Mezzetti**	isabella.mezzetti@mcri.edu.au	Biobanking Staff
**Karina De La Cruz**	karina.delacruz@mcri.edu.au	Biobanking Staff
**Ronita Singh**	ronita.singh@mcri.edu.au	Biobanking Staff
**Frances Oppedisano**	frances.oppedisano@mcri.edu.au	Laboratory Manager
**Study Visit and Phone call Staff**		
**Veronica Abruzzo**	veronica.abruzzo@mcri.edu.au	Visit coordinator; Senior Research Nurse Coordinator
**Joyce Chan**	joyce.chan@mcri.edu.au	Project Assistant
**Jac Cushnahan**	jac.cushnahan@mcri.edu.au	Clinic Staff
**Emma Burrell**	emma.burrell@mcri.edu.au	Clinic Staff
**Jamie Wedderburn**	jamie.wedderburn@mcri.edu.au	Clinic Staff
**Sarah Fowler**	sarah.fowler@mcri.edu.au	Clinic Staff
**Patricia Bimboese**	patricia.bimboese@rch.org.au	Clinic Staff; Start-up Support; Paediatric Trainee, RCH
**Hannah Elborough**	hannah.elborough@mcri.edu.au	Clinic Staff
**Jill Nguyen**	jill.nguyen@mcri.edu.au	Clinic Staff
**Stephanie Reynolds**	steph.reynolds@mcri.edu.au	Clinic Staff
**Liz O'Donnell**	liz.odonnell@mcri.edu.au	Clinic Staff
**Kirsty Bowes**	kirsty.bowes@mcri.edu.au	Clinic Staff
**Olivia Elkington**	oelkington@student.unimelb.edu.au	Clinic Staff
**Sam Macalister**	smacalister@student.unimelb.edu.au	Clinic Staff
**Catherine Flynn**	catherineflynn@hotmail.com.au	Clinic Staff
**Norine Ma**	norinem@student.unimelb.edu.au	Clinic Staff

BRACE trial Consortium Group List_29MAR2021

**Bojana Gladanac**	bgladanac@student.unimelb.edu.au	Clinic Staff
**Morgan Bealing**	mbealing@student.unimelb.edu.au	Clinic Staff
**Isabelle Ooi**	wooi@student.unimelb.edu.au	Clinic Staff
**Nadia Olivier**	nadiaolivier94@gmail.com	Clinic Staff
**Monique Fernandez**	mfernandez1@student.unimelb.edu.au	Clinic Staff
**Angela Younes**	ayounes@student .unimelb.edu.au	Clinic Staff
**Kieran Fahey**	kfahey@student.unimelb.edu.au	Clinic Staff
**Lisa Shen**	Ishen1@student.unimelb.edu.au	Clinic Staff
**Jesutofunmi Mojeed**	jmojeed@student.unimelb.edu.au	Clinic Staff
**Orygen Group**		Volunteers; phone calls to participants
**Pharmacy**		
**Donna Legge**	Donna.Legge@rch.org.au	Clinical Trial Pharmacist
**Annie Cobbledick**	Annie.cobbledick@rch.org.au	Pharmacist
**Kee Lim**	KeeLin.Lim@rch.org.au	Pharmacist
**Jo Cheah**	jo.cheah@rch.org.au	Pharmacist
**Jason Bell**	jason.bell@rch.org.au	Pharmacist
**Nurse Immuniser**		
**Sonja Elia**	sonja.elia@rch.org.au	Head of Immunisation Service
**Skye Miller**	Skye.Miller@rch.org.au	Vaccinator
**Lynne Addlem**	lynne.addlem@rch.org.au	Vaccinator
**Narelle Jenkins**	Narelle.Jenkins@rch.org.au	Vaccinator
**Nadine Henare**	Nadine.Henare@rch.org.au	Vaccinator
**Clare Brophy**	Clare.brophy@mcri.edu.au	Vaccinator
**Sigrid Pitkin**	sigrid.pitkin@mcri.edu.au	Vaccinator
**Anna Bourke**	anna.bourke@mcri.edu.au	Vaccinator
**Francesca Machingaifa**	Francesca.Machingaifa@mcri.edu.au	Vaccinator
**Kirsten Mitchell**	kirsten.mitchell@mcri.edu.au	Vaccinator

BRACE trial Consortium Group List_29MAR2021

**Kate Wall**	Kate.wall@mcri.edu.au	Vaccinator
**Safety and Quality Monitoring**		
**Laure Pittet**	laure.pittet@mcri.edu.au	Investigator; Clinical Safety Lead
**Paola Villanueva**	Paola.Villanueva@rch.org.au	Safety Lead, PhD student
**Nigel Crawford**	nigel.crawford@mcri.edu.au	Director of SAEFVIC
**Wendy Norton**	Wendy.norton@mcri.edu.au	VIC Safety Representative
**Ushma Wadia**	ushma.Wadia@telethonkids.org.au	WA Safety Representative
**Alice Sawka**	alice.Sawka@sa.gov.au	SA Safety Representative
**Ketaki Sharma**	Ketaki.sharma@health.nsw.gov.au	NSW Safety Representative
**Darren Troeman**	D.P.R.Troeman@umcutrecht.nl	Former Netherland Safety Representative
**Cristina Prat Aymerich**	C.PratAymerich-2@umcutrecht.nl	Spain and Netherland Safety Representative
**Prof Adilia Warris**	A.Warris@exeter.ac.uk	UK Safety Representative
**Mariana Garcia Croda**	mgcroda@gmail.com	Brazil Safety Representative (Mato Grosso Do Sul)
**Jorge Rocha**	jorgeluiz.rocha@yahoo.com.br	Brazil Safety Representative (Rio de Janeiro)
**Bruno Jardim**	brunojardim89@hotmail.com	Brazil Safety Representative (Manaus)
**MCRI Start-up Team**		
**Carolyn Stewart**	carolyn.stewart@mcri.edu.au	Start-up Support; Business and Operations Manager, MCTC
**Katherine Lieschke**	katherine.lieschke@mcri.edu.au	Start-up Support; RCH Research Ethics and Governance, MCTC
**Jess Bucholc**	jess.bucholc@mcri.edu.au	Start-up Support Staff; coordination Monash
**Samantha Bannister**	Samantha.Bannister@rch.org.au	Start-up Support; PhD Student, Infectious Diseases Group, MCRI
**Eva Sudbury**	evasudbury@gmail.com	Start-up Support; PhD Student, Infectious Diseases Group, MCRI
**Emma Watts**	emma.watts@mcri.edu.au	Start-up Support; Regulatory Support
**Angela Young**	angela.young@mcri.edu.au	Start-up Support
**Chris Richards**	chris.richards@mcri.edu.au	Start-up Support Grants
**Christina Guo**	christina.guo@mcri.edu.au	Start-up Support; Paediatric Trainee, RCH
**Helen Thomson**	helen.thomson@mcri.edu.au	Support Grants; Research Manager, Asia-Pacific Health, MCRI
**Stephanie Firth**	stephanie.firth@mcri.edu.au	Start-up Support

BRACE trial Consortium Group List_29MAR2021

**Marianna Ciaverella**	marianne.ciavarella@mcri.edu.au	Support Grants; Grants Manager, Grants Office, MCRI
**Matthew Hannan**	matthew.hannan@mcri.edu.au	Support Grants; Head of Engagement & Philanthropy, MCRI
**Erin Hill**	erin.hill@mcri.edu.au	Start-up Support; Population Allergy, MCRI
**Beatriz Camesella**	beatriz.camesellaper@mcri.edu.au	Start-up Support; Population Allergy, MCRI
**Ashleigh Rak**	ashleigh.rak@mcri.edu.au	Start-up Support Staff; coordination
**Sasha Odoi**	sasha.odoi@mcri.edu.au	Start-up Support Staff; coordination
**Megan Mathers**	megan.mathers@mcri.edu.au	Start-up Support Staff; coordination
**Sri Joshi**	sri.joshi@mcri.edu.au	Start-up support; Database Manager, MCTC
**Sophie Agius**	sophie.agius@mcri.edu.au	Start-up Support; logistics and consumables
**Alison Burns**	alison.burns@mcri.edu.au	Start-up Support; logistics and consumables
**Leah Steve**	leah.steve@mcri.edu.au	Start-up Support; logistics and consumables
**John Carlin**	john.carlin@mcri.edu.au	Director, Clinical Epidemiology & Biostatistics, MCRI
**Name**	**Email**	**Role**
**Steering Committee**		
**Andrew Steer**	andrew.steer@rch.org.au	Theme Director, Infection & Immunity, MCRI
**Ann Ginsberg**	ann.ginsberg@gatesfoundation.org	Deputy Director of TB Vaccines, Bill and Melinda Gates Foundation
**David Paterson**	d.paterson1@uq.edu.au	BRACE Steering Committee Chair
**Kanta Subbarao**	kanta.subbarao@influenzacentre.org	Director, WHO Collaborating Centre; Peter Doherty Institute
**Kim Mulholland**	kim.mulholland@mcri.edu.au	New Vaccines Group, MCRI
**Peter Richmond**	peter.richmond@uwa.edu.au	Principal Investigator WA, TKI
**Nigel Curtis**	nigel.curtis@rch.org.au	BRACE Chief Principal Investigator
**External Advisors**		
**Mihai Netea**	mihai.netea@radboudumc.nl	Head of Experimental Medicine, The Netherlands
**Richard Malley**	richard.malley@childrens.harvard.edu	Boston Children's Hospital; Harvard Medical School
**Adam Finn**	adam.finn@bristol.ac.uk	University of Bristol; NIHR Clinical Research Network; WHO
**Denise Faustman**	dfaustman@mgh.harvard.edu	Harvard University; Massachusetts General Hospital
**Frank Shann**	shannf@netspace.net.au	Start-up Support; Department of Paediatrics, University of Melbourne

BRACE trial Consortium Group List_29MAR2021

**Kim Mulholland**	kim.mulholland@mcri.edu.au	New Vaccines Group, MCRI
**Regulatory, Legal and Finances**		
**Penny Glenn**	penny.glenn@mcri.edu.au	Senior Legal Counsel
**Laura Galletta**	laura.galletta@mcri.edu.au	Senior Study Coordinator, MCTC
**Amandine Philippart De Floy**	amandine.philippart@mcri.edu.au	Senior Contract Manager & Privacy Officer
**Neil Harker**	neil.harker@mcri.edu.au	Financial Consultant (Insurance & Risk)
**Karen Dalton**	karen.dalton@mcri.edu.au	Finance
**Galina Fidler**	galina.fidler@mcri.edu.au	Finance
**App Development**		
**Ivy Xie**	ivy@curvetomorrow.com.au	Product Manager, Curve Tomorrow
**Sandy Buchanan**	sandy@weguide.com.au	COO & Head of Partnerships at WeGuide
**Thijs Sondag**	thijs.sondag@mcri.edu.au	Innovation Consultant, Curve Tomorrow
**Media and Communications**		
**Harriet Edmund**	harriet.edmund@mcri.edu.au	BRACE Communications Officer
**Michelle Wearing-Smith**	michelle.wearingsmith@mcri.edu.au	Head of Communications & Marketing
**Tom Keeble**	tom.keeble@mcri.edu.au	Communications Manager
**Bridie Byrne**	bridie.byrne@mcri.edu.au	Communications Specialist
**Belle Ngien**	belle.ngien@mcri.edu.au	Marketing Projects Officer
**Fran Noonan**	fran.noonan@mcri.edu.au	Content Manager
**Logistics - Consumables**		
**Sophie Agius**	sophie.agius@mcri.edu.au	Start-up Support
**Alison Burns**	alison.burns@mcri.edu.au	Regular Stocks and Shipping
**Leah Steve**	leah.steve@mcri.edu.au	Regular Stocks and Shipping
**IT Support**		
**Wayne Mather**	wayne.mather@mcri.edu.au	CIO, MCRI
**Nick Evans**	nick.evans@mcri.edu.au	IT Infrastructure Manager, MCRI
**Luke Stevens**	luke.stevens@mcri.edu.au	REDCap expert

BRACE trial Consortium Group List_29MAR2021

VIC – MONASH, EPWORTH

Name	Email	Role
**VIC Sites**		
**Niki Tan**	niki.tan@anaestheticservices.com.au	Epworth Healthcare PI
**Diane Dawson**	Di.Dawson@epworth.org.au	Site Coordinator
**Victoria Gordon**	victoria.gordon@epworth.org.au	Research Nurse
**Thilakavathi Chengodu**	thili.chengodu@epworth.org.au	Research Nurse
**Tony Korman**	tony.korman@monash.edu	Monash PI
**Jess O'Bryan**	Jessica.O'bryan@monashhealth.org	Site Coordinator

WA – PCH, FSH, SCGH

Name	Email	Role
**WA Sites**		
**Peter Richmond**	peter.richmond@uwa.edu.au	Principal Investigator WA, TKI, PCH
**Tobias Kollmann**	Tobias.Kollmann@telethonkids.org.au	Biosample and Data Use Committee WA Representative
**Ushma Wadia**	ushma.Wadia@telethonkids.org.au	WA Safety Representative
**Krist Ewe**	Yean.ewe@health.wa.gov.au	Sub-investigator
**Jaslyn Ong**	jaslyn.ong@health.wa.gov.au	Sub-investigator
**Joanne Ong**	Joanne.ong@telethonkids.org.au	Sub-investigator
**Andrea Meehan**	Andrea.meehan@health.wa.gov.au	Sub-investigator
**Carolyn Finucane**	Carolyn.finucane@telethonkids.org.au	Research Nurse

BRACE trial Consortium Group List_29MAR2021

**Rachael Wallace**	Rachael.wallace@health.wa.gov.au	Research Nurse
**Annabelle Arnold**	Annabelle.arnold@health.wa.gov.au	Research Nurse (also helped at SCGH)
**Jemma Dunnill**	Jemma.dunnill@telethonkids.org.au	Research Nurse/follow up
**Catherine Power**	Catherine.power@health.wa.gov.au	Vaccinator
**Glady Perez**	Gladymar.perez@telethonkids.org.au	Vaccinator
**Jane Jones**	Jane.jones@telethonkids.org.au	Vaccinator/phlebotomist
**Camille Gibson**	Camille.gibson@telethonkids.org.au	Vaccinator/phlebotomist
**Fiona McDonald**	Fiona.mcdonald@telethonkids.org.au	Vaccinator/phlebotomist/follow up
**Lisa Stiglmayer**	Lisa.stiglmayer@health.wa.gov.au	Vaccinator
**Sally Rogers**	Sally.rogers@health.wa.gov	Vaccinator
**Lance Jarvis**	Lance.jarvis@health.wa.gov.au	Vaccinator
**Alexandra Truelove**	Alexandra.trulove@health.wa.gov.au	Vaccinator
**Jennifer Kent**	Jennifer.kent@telethonkids.org.au	Vaccinator
**Christina Anthony**	Christina.anthony@telethonkids.org.au	Phlebotomist
**Beth Arrowsmith**	Beth.arrowsmith@telethonkids.org.au	Phlebotomist
**Heidi Hutton**	Heidi.hutton@telethonkids.org.au	Phlebotomist
**Lorraine Flynn**	Lorraine.flynn@health.wa.gov.au	Phlebotomist
**Lisa Montgomery**	Lisa.montgomery@telethonkids.org.au	Phlebotomist/lab
**Jan Jones**	Jan.jones@telethonkids.org.au	Lab
**Nikki Schultz**	Nikki.Schultz@telethonkids.org.au	Lab
**Sonia McAlister**	Sonia.mcalister@telethonkids.org.au	Lab
**Sharon Clark**	Sharon.clark@telethonkids.org.au	Lab
**Kimberley Parkin**	Kimberley.parkin@telethonkids.org.au	Lab
**Holly Richmond**	holly.richmond@telethonkids.org.au	Follow up staff (part-time)/Site Coordinator contact
**Karen Jones**	Karen.a.jones@uwa.edu.au	Follow up staff
**Margaret Shave**	Margaret.shave@health.wa.gov.au	Pharmacist
**Melissa O’Donnell**	Melissa.o’donnell@health.wa.gov.au	Pharmacist

BRACE trial Consortium Group List_29MAR2021

**Debbie Lalich**	Debbie.lalich@health.wa.gov.au	Pharmacist
**Justin Waring**	Justin.waring@health.wa.gov.au	Medical Director of the Anita Clayton Service- Follow up of the BCG vaccination adverse events of concern and also provision of nurses to train nurses regarding intradermal vaccination
**Laurens Manning**	laurens.manning@uwa.edu.au	Fiona Stanley Hospital PI
**Erin Latkovic**	erin.latkovic@health.wa.gov.au	Site Coordinator
**Michelle England**	michelle.england@health.wa.gov.au	FSH
**Michaela Lucas**	michaela.lucas@uwa.edu.au	Sir Charles Gairdner Hospital PI
**Susan Herrmann**	susan.herrmann@uwa.edu.au	Site Coordinator
**Hana Karuppasamy**	Hana.karuppasamy@health.wa.gov.au	Research Nurse
**Annabelle Arnold**	Annabelle.Arnold@health.wa.gov.au	Research Nurse
**Zaheerah Haywood**	zaheerah.haywood@health.wa.gov.au	Follow-up Staff
**Ruth Warren**	ruth.warren@uwa.edu.au	Follow-up Staff
**Nat Eiffler**	Nat.Eiffler@telethonkids.org.au	WA Participant Communication Support

SA – RAH, WCH

**Name**	**Email**	**Role**
**SA Sites**		
**Prof David Lynn**	David.lynn@sahmri.com	Principal Investigator SA, SAHMRI
**Prof Steve Wesselingh**	Steve.Wesselingh@sahmri.com	Sub Investigator
**Liddy Griffith**	liddy.griffith@sahmri.com	Senior Study Coordinator
**Domenic Sacca**	domenic.sacca@sahmri.com	Study Coordinator
**Angela Markow**	angela.markow@sahmri.com	Assistant Study Coordinator
**Rochelle Botten**	rochelle.botten@sahmri.com	Study Coordinator
**Miriam Lynn**	Miriam.Lynn@sahmri.com	Lab Researcher
**Stephen Blake**	stephen.blake@sahmri.com	Lab Researcher
**Natalie Stevens**	natalie.stevens@sahmri.com	Lab Researcher

BRACE trial Consortium Group List_29MAR2021

**Georgina Eden**	Georgina.eden@sahmri.com	Lab Researcher
**Saoirse Benson**	Saoirse.benson@sahmri.com	Lab Researcher
**Tee Yee Chern**	Tee.Yee@sahmri.com	Lab Researcher
**Jane James**	jane.james@sahmri.com	Lab Researcher
**Dr Simone Barry**	Simone.barry@sa.gov.au	Royal Adelaide Hospital PI
**Catriona Doran**	Catriona.Doran@sa.gov.au	Site Coordinator
**Alice Sawka**	alice.Sawka@sa.gov.au	SA Safety Representative
**Prof Helen Marshall**	helen.marshall@adelaide.edu.au	Women's and Children's Hospital PI
**Christine Heath**	christine.heath@adelaide.edu.au	Site Coordinator
**Mark McMillan**	mark.mcmillan@adelaide.edu.au	Study Coordinator
**Meredith Krieg**	meredith.krieg@adelaide.edu.au	Research Nurse
**Mary Walker**	mary.walker@adelaide.edu.au	Research Nurse
**Louise Goodchild**	louise.goodchild@adelaide.edu.au	Research Nurse
**Dr Sue Evans**	sue.evans@adelaide.edu.au	Research Doctor

NSW – WC, WH, POWH, SCH, ST V'S

Name	Email	Role
**NSW Sites**		
**A/Prof Nick Wood**	nicholas.wood@health.nsw.gov.au	Principal Investigator NSW, WC
**Prof Craig Munns**	craig.munns@health.nsw.gov.au	Principal Investigator NSW, WC
**Aiken Dao**	aiken.dao@sydney.edu.au	Site Coordinator
**Katrina Sterling**	katrina.sterling@health.nsw.gov.au	Site Coordinator (WC, POWH, SCH)
**Lisa Pelayo**	lisa.pelayo@health.nsw.gov.au	NSW State Coordinator
**Andrew Dunn**	Andrew.Dunn@health.nsw.gov.au	Clinic Staff
**Therese Baulman**	therese.baulman@health.nsw.gov.au	Vaccinator
**Mary Corbett**	mary.corbett@health.nsw.gov.au	Vaccinator

**Maria Desylva**	maree.desylva@health.nsw.gov.au	Vaccinator
**Rosemary Joyce**	rosemary.joyce@health.nsw.gov.au	Clinical Nurse Consultant
**Evangeline Gardiner**	Evangeline.Gardiner@health.nsw.gov.au	Research Assistant
**A/Prof Mark Douglas**	mark.douglas@sydney.edu.au	Westmead Hospital PI
**Clinton Colaco**	Clinton.colaco@health.nsw.gov.au	Sub-investigator
**Kate Hamilton**	Kate.Hamilton@health.nsw.gov.au	Site Coordinator
**Dr Brendan McMullan**	brendan.mcmullan@health.nsw.gov.au	Sydney Children's Hospital, Randwick PI
**Pamela Palasanthiran**	pamela.palasanthiran@health.nsw.gov.au	Clinic Staff
**Adam Bartlett**	adam.bartlett@health.nsw.gov.au	Clinic Staff
**Phoebe Williams**	phoebe.williams1@health.nsw.gov.au	Clinic Staff
**A/Prof Jeffrey Post**	jeffrey.post@health.nsw.gov.au	Prince of Wales Hospital PI
**Renier Lagunday**	Renier.Lagunday@health.nsw.gov.au	Clinic Staff
**Justin Beardsley**	Justin.beardsley@health.nsw.gov.au	Clinic Staff
**Kristen Overton**	Kristen.overton@health.nsw.gov.au	Clinic Staff
**Nikki Bergant**	nikki.bergant6@gmail.com	Vaccinator
**Dr Anthony Byrne**	anthony.byrne@svha.org.au	St Vincent's Hospital Sydney PI
**Lee Mead**	Lee.Mead@svha.org.au	Site Coordinator
**Yasmeen Al-Hindawi**	Yasmeen.Al-hindawi@svha.org.au	Site Investigator SVHS
**Sarah Barney**	Sarah.Barney@svha.org.au	Clinical Trial Assistant
**Nikki Bergant**	nikki.bergant6@gmail.com	Vaccinator
**Dr Rama Kandasamy**	Rama.Kandasamy@health.nsw.gov.au	Key Contact
**Twinkle Bahaduri**	twinkle.bahaduri@health.nsw.gov.au	NSW Ethics/Governance Submission Coordinator
**Ketaki Sharma**	Ketaki.sharma@health.nsw.gov.au	NSW Safety Representative

BRACE trial Consortium Group List_29MAR2021

THE NETHERLANDS

Name	Email	Role
**UMC Utrecht**		
**Prof Marc Bonten**	m.j.m.bonten@umcutrecht.nl	Sponsor Investigator - Europe PI
**Leo Van Den Heuvel**	L.M.C.v.denHeuvel-2@umcutrecht.nl	
**Cristina Prat Aymerich**	C.PratAymerich-2@umcutrecht.nl	Coordinating Investigator - Europe
**Nicolette van Sluis**	N.L.J.vanSluis@umcutrecht.nl	Project Manager European Projects
**Astrid Suiker**	astrid.suiker@juliusclinical.com	Clinical Research Associate
**Radhika Ganpat**	j.r.ganpat@umcutrecht.nl	Clinical Trial Assistant
**Marije van der Waal**	M.vanderWaal-13@umcutrecht.nl	Junior Program Manager (Spain)
**Sigrid van der Veen**	s.vanderveen-11@umcutrecht.nl	Junior Program Manager (Dutch)
**Engelien Septer-Bijleveld**	engelien.septerbijleveld@juliusclinical.com	Sr. Project Manager
**Titia Leurink**	titia.leurink@juliusclinical.com	Jr. Project Manager
**Chantal van de Ven**	chantal.vandeven@juliusclinical.com	Clinical Trial Assistant
**Axel Janssen**	a.b.janssen-9@umcutrecht.nl	Head of Central Laboratory
**Beatrijs Wolters**	b.e.wolters-2@umcutrecht.nl	
**Darren Troeman**	D.P.R.Troeman@umcutrecht.nl	Medical Monitor
**Toos Lemmers**	A.D.P.Lemmens-2@umcutrecht.nl	Study Coordinator
**Endriaen Prajitno**	E.Prajitno@umcutrecht.nl	Pharmacist
**Anne Boon**	A.Boon@umcutrecht.nl	Laboratory
**Marjoleine van Opdorp**	M.J.W.vanOpdorp@umcutrecht.nl	Research Nurse
**Amphia Hospital**		
**Prof Jan Kluytmans**	jankluytmans@gmail.com	Hospital PI
**Dr M.M.L (Miranda) van Rijen**	MVanRijen@amphia.nl	Sub Investigator
**Dr Wouter Bijllaardt**	WBijllaardt@amphia.nl	Sub Investigator
**Linda van Mook**	Lmlresearch@amphia.nl; Lvanmook@amphia.nl	Study Coordinator

BRACE trial Consortium Group List_29MAR2021

**Jannie Romme**	Lmlresearch@amphia.nl; JRomme@amphia.nl	Study Coordinator
**H. van Onzenoort**	trialbureau@amphia.nl	Pharmacist
**Vivian Zwart**	vzwart@amphia.nl	Vaccinator
**Rijnstate Hospital**		
**Jet Gisolf**	JGisolf@rijnstate.nl	Hospital PI
**Dr Robert Jan Hassing**	RHassing@rijnstate.nl	Sub Investigator
**Harald Verheij**	HVerheij@rijnstate.nl	Study Coordinator
**P.M.G. Filius**	trialapotheek@rijnstate.nl	Pharmacist
**Frances Greven**	Fgreven@rijnstate.nl	Research Nurse
**Lieke Preijers**	LPreijers@rijnstate.nl	Research Nurse
**P.M.Verhoeven**	pverhoeven@rijnstate.nl	Research Nurse
**J. H. van Leusen**	jvanleusen@rijnstate.nl	Vaccinator
**R. C. Pon**	rpon@rijnstate.nl	Vaccinator
**Sille Pelser**	spelser@rijnstate.nl	Research Nurse
**Marlot Uffing**	muffing@rijnstate.nl	Research Nurse
**Danique Huijbens**	Dhuijbens@rijnstate.nl	Research Nurse
**Noord West Ziekenhuis**		
**Dr Wim Boersma**	w.boersma@nwz.nl	Hospital PI
**Dr Nienke Paternotte**	n.paternotte@nwz.nl	Sub Investigator
**Lida Stooper**	researchlongziekten@nwz.nl	Study Coordinator
**Anke Rol**	researchlongziekten@nwz.nl	Study Coordinator
**Paul Kloeg**	p.kloeg@nwz.nl	Pharmacist
**Khanh Nguyen**	k.nguyen@nwz.nl	Pharmacy Assistant
**Kitty Molenaar**	c.a.m.molenaar-groot@nwz.nl	Pharmacy Coordinator
**Radboud UMC**		
**Dr Jaap ten Oever**	Jaap.tenOever@radboudumc.nl	Hospital PI
**Dr Simone Moorlag**	Simone.Moorlag@radboudumc.nl	Sub Investigator

BRACE trial Consortium Group List_29MAR2021

**Dr Esther Taks**	Esther.Taks@radboudumc.nl	Sub Investigator
**Prof Mihai Netea**	Mihai.Netea@radboudumc.nl	Sub Investigator
**Rob ter Heine**	R.terHeine@radboudumc.nl	Pharmacist
**Helga Dijkstra**	Helga.Dijkstra@radboudumc.nl	Laboratory
**St Antonius Hospital**		
**Dr Bob Meek**	b.meek@antoniusziekenhuis.nl	Hospital PI
**Dr Nienke Roescher**	n.roescher@antoniusziekenhuis.nl	Sub Investigator
**Kitty Blauwendraat**	k.blauwendraat@antoniusziekenhuis.nl	Study Coordinator
**Carmen Zhou**	c.zhou@antoniusziekenhuis.nl	SC/Vaccinator
**Houda Harbech**	h.harbech@antoniusziekenhuis.nl	SC/Vaccinator
**Menno te Riele**	m.te.riele@antoniusziekenhuis.nl	SC/Vaccinator

SPAIN

Name	Email	Role
**Mutua Terrassa University Hospital**		
**Tomás Perez Porcuna**	tomasperez@mutuaterrassa.es	Hospital PI
**Dr Esther Calbo**	ecalbo@mutuaterrassa.es	Sub Investigator
**Cristina Badia Marti**	cbadia@mutuaterrassa.cat	Study Coordinator
**Susana Gonzalez Marcos**		Sub Investigator
**Sonia Sallent**	ssallent@mutuaterrassa.es	Study Nurse
**Maria Roser Font**	rfont@mutuaterrassa.es	Study Nurse
**University Hospital Germans Trias I Pujol**		
**Dr Antoni Rosell**	arosellg.germanstrias@gencat.cat	Hospital PI
**Adrian Siles Baena**	asiles@igtp.cat	Pharmacist
**Jose Dominguez**	jadomb69@gmail.com	Laboratory
**Ana Maria Barriocanal**	ambarriocanal@igtp.cat	Study Coordinator

BRACE trial Consortium Group List_29MAR2021

**Alicia Lacoma**	alacoma@igtp.cat	Study Coordinator
**Maria Esteve**	mariaesteve.germanstrias@gencat.cat	Researcher
**Irma Casas**	icasas.germanstrias@gencat.cat	Researcher
**Guillermo Mena**	guillemena.germanstrias@gencat.cat	Researcher
**Anabel Barriocanal**	ambarriocanal@igtp.cat	Researcher
**Sandra Vidal**	svidal@igtp.cat	Research Nurse
**Gemma Molina**	gmolina@igtp.cat	Researcher
**Irene Latorre**	ilatorre@igtp.cat	Researcher
**Barbara Molina**	bmolina@igtp.cat	Researcher
**Raquel Villar**	rvillar@igtp.cat	Researcher
**Ester Valls**		Researcher
**Patricia Comella**		Researcher
**University Hospital Virgen Macarena**		
**Prof Jesús Rodríguez-Baño**	jesusrb@us.es	Hospital PI
**Lydia Barrera**	redancraid.hvm.sspa@juntadeandalucia.es	Study Coordinator
**Enriqueta Tristán**	redancraid.hvm.sspa@juntadeandalucia.es	Study Coordinator
**Jose Manuel Carrerero**	ensayosclinicosmacarena@yahoo.es	Pharmacist
**Carlos García**	administracion.eecc.hvm.sspa@juntadeandalucia.es	Clinical Trials Management
**Almudena de la Serna**	almudena.serna@juntadeandalucia.es	Clinical Trials Management
**Virginia Palomo Jiménez**	virgi2015@hotmail.com	Study Coordinator
**Angel Dominguez Castellano**	adomin60@gmail.com	Sub Investigator
**Maria Jose Rios Vilegas**	mjriosvillegas@gmail.com	Sub Investigator
**Reyes Lopez Marques**	mreyes.lopez.sspa@juntadeandalucia.es	Sub Investigator
**Nicolas Navarrette**	alambrada21@gmail.com	Sub Investigator
**Maria Dolores del Toro Lopez**	mdeltoro@us.es	Sub Investigator
**Rosario Vigo Ortega**	charovo2102@gmail.com	Research Nurse
**David Gutierrez Campos**	david.gutierrezcampos@hotmail.com	Research Nurse

BRACE trial Consortium Group List_29MAR2021

**Ana Belen Martin Gutierrez**	anab.martin.gutierrez@outlook.com	Research Nurse
**Marie-Alix Clement Espindola**	mariealixclement@gmail.com	Research Nurse
**Encarnacion Ramirez de Arellano**	encarnacion.ramirezarellano.sspa@juntadeandalucia.es	Laboratory
**Alvaro Pascual Hernandez**		Laboratory
**Maria Carmen Roque**	mariac.roque@juntadeandalucia.es	Scientific Coordinator
**Teresa Rodrigues**	tereler@gmail.com	Laboratory
**Raquel Serrano**	ryrserrano@gmail.com	Laboratory
**Bouchra Daitiri**		Pharmacist
**Ines Portillo Calderón**	ines6290@gmail.com	Laboratory
**Natalia Bustos**	natalia.bustos@juntadeandalucia.es	Study Coordinator
**Rocio del Alba Rey Morillo**	rocio.rey@juntadeandalucia.es	Clinical Research Associate
**University Hospital Cruces**		
**Dr. Josune Goikoetxea**	ANEJOSUNE.GOIKOETXEAAGIRRE@osakidetza.eus	Hospital PI
**Atsegiñe Cangas**	ATSEGINE.CANGAGARCES@osakidetza.eus	Study Coordinator
**Cristina Perez**		Pharmacist
**Leticia Jorge**	ensayosclinicos.farmaciacruces@osakidetza.eus	Pharmacist
**Erika Castro**	ERIKA.CASTROAMO@osakidetza.eus	Laboratory
**Raquel Coya**	RAQUEL.COYAGUERRERO@osakidetza.eus	Laboratory
**Marqués de Valdecilla University Hospital**		
**Dr María Carmen Fariñ Álvarez**	mcarmen.farinas@scsalud.es	Hospital PI
**Dr Francisco Arnaiz de las Revillas Almajano**	francisco.arnaizlasrevillas@scsalud.es	Sub Investigator
**Claudia González Rico**	claudia.glez.rico@gmail.com; claudia.gonzalez@scsalud.es	Site Coordinator
**Dr Teresa Giménez Poderos**	farmacia_ensayos.humv@scsalud.es	Pharmacist
**Dr Jorge Calvo Montes**	jorge.calvo@scsalud.es	Laboratory
**Olga Valero**	olga.valero@scsalud.es	Study Nurse
**Noelia Vega**	noelia.vega@scsalud.es	Study Coordinator
**Nuria Sanchez**		Study Nurse

BRACE trial Consortium Group List_29MAR2021

**Blanca Sanchez**		Study Coordinator
**Pilar Bohedo Garcia**	mpilar.bohedo@scsalud.es	Vaccinator
**Manuel Gutierrez Cuadra**	manuel.gutierrezc@scsalud.es	Sub Investigator

THE UK

Name	Email	Role
**University of Exeter/Exeter Clinical Trials Unit**		
**Prof John Campbell**	john.campbell@exeter.ac.uk	Principal Investigator (UK)
**Prof Adilia Warris**	A.Warris@exeter.ac.uk	Co-Principal Investigator (UK)
**Shelley Rhodes**	S.Rhodes@exeter.ac.uk	Senior Trial Manager
**Lynne Quinn**	L.Quinn@exeter.ac.uk	ExeCTU Director of Operations
**Abby O'Connell**	a.j.oconnell@exeter.ac.uk	Trial Manager
**Emily Fletcher**	E.Fletcher@exeter.ac.uk	Trial Manager
**Amy McAndrews**	A.McAndrew@exeter.ac.uk	Trial Manager
**Bethany Whale**	B.Whale@exeter.ac.uk	Trial Coordinator
**Harry Tripp**	H.E.Tripp@exeter.ac.uk	Data Manager
**Rosie Owens**	R.S.Owens@exeter.ac.uk	Data Manager
**Liam Fouracre**	L.Fouracre@exeter.ac.uk	Data Manager
**Phoebe Dawe**	P.Dawe@exeter.ac.uk	Data Manager
**Jakob Onysk**	jao208@exeter.ac.uk	Data Manager
**Helen Catterick**	helen.catterick@nhs.net	Safety Medical Doctor
**Lorrie Symons**	lorrie.symons@nhs.net	Safety Medical Doctor
**Georgina Newman**	gn272@exeter.ac.uk	Safety Medical Doctor
**Alison Gifford**	ag839@exeter.ac.uk	Safety Medical Doctor
**Clare Seamark**	clare.seamark@nhs.net	Safety Medical Doctor
**David Seamark**	david.seamark@nhs.net	Safety Medical Doctor

**Christopher Martin**	C.Martin4@exeter.ac.uk	Pharmacist
**Marcus Mitchell**	M.R.P.Mitchell@exeter.ac.uk	Laboratory Technician
**Louise Vennells**	L.Vennells@exeter.ac.uk	Senior Press and Media Manager
**St Leonard's Practice**		
**Dr Alex Harding**	a.m.harding@nhs.net	Practice PI
**Gemma Lockhart**	gemma.lockhart@nhs.net	Nurse
**Kate Sidaway-Lee**	k.sidaway-lee@nhs.net	Research Fellow
**Ide Lane Surgery**		
**Dr Daniel Webber-Rookes**	d.webber-rookers@nhs.net	Practice PI
**Sarah Manton**	sarahmanton@nhs.net	Nurse
**Dr Sam Hilton**	sam.hilton@nhs.net	Clinician
**Travel Clinic**		
**James Moore**	james@travelhealthconsultancy.co.uk	Clinic PI/Nurse
**Royal Devon and Exeter NHS Foundation Trust**		
**Dr Michael Gibbons**	michael.gibbons2@nhs.net	Hospital PI
**Josephine Studham**	j.m.studham@exeter.ac.uk	NIHR Exeter Clinical Research Facility Manager
**Bridget Knight**	B.A.Knight@exeter.ac.uk	Research Nurse
**Julie Moss**	Julie.moss10@nhs.net	Research Nurse
**Sarah Statton**	s.statton@nhs.net	Clinician
**Glendevon Medical Practice**		
**Tamsin Venton**	Tamsin.venton@nhs.net	Practice PI
**Will Moyle**	will.moyle@nhs.net	Practice Manager
**Robert Harrison**	robert.harrison2@nhs.net	GP
**Rachel Dixon**	rdixon3@nhs.net	GP
**Lydia Hall**	lydiahall@nhs.net	Research Practioner
**Jill Fairweather**	jill.fairweather@nhs.net	Practice Nurse

BRACE trial Consortium Group List_29MAR2021

BRAZIL – MATO GROSSO DO SUL

Name	Email	Role
**UEMS, Mato Grosso do Sul**		
**Julio Croda**	juliocroda@gmail.com	Principal Investigator (MS)
**Prof Roberto Oliveira**	roberto@uems.br; prof.roberto@me.com	Study Coordinator
**Patricia Vieira**	patriciavieira.s@hotmail.com	Study Coordinator
**Daniel Tsuha**	danlnca@gmail.com	Data Manager
**Marco Puga**	marco.m.puga@gmail.com	Laboratory Lead
**Bruna Tayara Leopoldina Meireles**	bruna.tayara@hotmail.com	Operator not blind
**Carolinne Abreu**	abreucarolinne5@gmail.com	Operator not blind
**Dyenyffer Stéffany Leopoldina dos Santos**	dyenyffer98@hotmail.com	Operator not blind
**Miriam de Jesus Costa**	myricosta29@gmail.com	Operator not blind
**Caroliny Veron Ramos**	carolinyvr19@gmail.com	Telephonist
**Claudinalva Ribeiro dos Santos**	claudinalva2009@gmail.com	Telephonist
**Guilherme Teodoro de Lima**	guilhermexaira@gmail.com	Telephonist
**Katya Martinez Almeida**	katyamartinez1982@gmail.com	Telephonist
**Matheus Machado Ramos**	mrmachadobiotec@gmail.com	Telephonist
**Wellyngthon Espindola Ayala**	wellyngtonespindola@gmail.com	Telephonist
**Bianca Maria Silva Menezes Arruda**	biancamenezes14@hotmail.com	Laboratory
**Camila Bitencourt de Andrade**	camilabitencourtdeandrade@gmail.com	Laboratory
**Débora dos Santos Silva**	d.rodruigues85@bol.com.br	Laboratory
**Joyce dos Santos Lencina**	joycedslencina@gmail.com	Laboratory
**Lais Alves da Cruz**	laismcr@outlook.com	Laboratory
**Mariana Mayumi Tadokoro**	mayumitadokoro@gmail.com	Laboratory
**Paulo Victor Rocha da Silva**	pvitor993@gmail.com	Laboratory
**Karla Regina Warszawski de Oliveira**	karlareginawo@gmail.com	Pharmaceutical Head

BRACE trial Consortium Group List_29MAR2021

**Andrea Antonia Souza de Almeida dos Reis Pereira**	farmaceuticaandreareis@gmail.com	Pharmaceutical
**Iara Rodrigues Fernandes**	iara_rfernandes@yahoo.com.br	Pharmaceutical
**Roberta Carolina Pereira Diogo**	roberta_rcpd@hotmail.com	Pharmaceutical
**Rodrigo Cezar Dutra Escobar**	rodrigo.escoba@outlook.com	Pharmaceutical
**Adelita Agripina Refosco Barbosa**	adelita.barbosa@hotmail.com	Nurse
**Adriely de Oliveira**	adriely.o.b@gmail.com	Nurse
**Felipe Zampieri Vieira Batista**	lipe_zampieri@hotmail.com	Nurse
**Hugo Miguel Ramos Vieira**	hugobossram@hotmail.com	Nurse
**Jhenyfer Thalyta Campos Angelo**	jhenyfer_thalyta@hotmail.com	Nurse
**Karla Lopes dos Santos**	karla.lds@hotmail.com	Nurse
**Leticia Ramires Figueiredo**	le.enf.ramires@gmail.com	Nurse
**Lilian Batista Silva Muranaka**	liabatistamuranaka@gmail.com	Nurse
**Thaynara Haynara Souza da Rosa**	thayouza@gmail.com	Nurse
**Fábio Mauricio Nogueira Gomes**	fabinhomng@hotmail.com	Driver
**Leandro Galdino Cavalcanti Gonçalves**	leandrogcg@gmail.com	Driver
**Mariana Garcia Croda**	mgcroda@gmail.com	Safety Monitoring
**Matheus Vieira de Oliveira**	vieiramatheus79@gmail.com	Data Manager
**Mayara Góes dos Santos**	goes_may@hotmail.com	Coordinator Assistant
**Fabiani de Morais Batista**	fabianimb@hotmail.com	Coordinator Assistant

BRAZIL – RIO DE JANEIRO

Name	Email	Role
**Rio de Janeiro**		
**Margareth Dalcolmo**	margarethdalcolmo@gmail.com	Principal Investigator (Rio)
**Glauce Dos Santos**	glaucedossantos@gmail.com	Study Coordinator
**Ana Paula Conceição de Souza**	souzaannadi@hotmail.com	Assistant Coordinator

BRACE trial Consortium Group List_29MAR2021

**Ivan Maia**	ivanramosnut@gmail.com	Data Manager
**Adriano Gomes**	adriano.gomes@ini.fiocruz.br	Laboratory Lead
**Alda Cruz**	alda@ioc.fiocruz.br	Laboratory Lead
**Samyra Almeida Da Silveira**	samybiologia@gmail.com	Laboratory RA
**Maria Luciana Silva De Freitas**	mluciana@id.uff.br	Laboratory RA
**Rosa Maria Plácido Pereira**	rosamplacido@gmail.com	Laboratory RA
**Gabriela Correa E Castro**	gabi.castro027@gmail.com	Laboratory RA
**Paulo Leandro Garcia Meireles Junior**	paulolgmj@gmail.com	Laboratory RA
**Erica Fernandes Silva**	ericafernandesfarma@gmail.com	Pharmacist
**Aline Gerhardt de Oliveira**	agerhardt@ensp.fiocruz.br	Pharmacist
**Cristiane Machado**	cmarcondes07@gmail.com	Pharmacist
**Jorge Rocha**	jorgeluiz.rocha@yahoo.com.br	Safety Lead
**Ligia Olivio**	ligiamon@gmail.com	Safety Medical Doctor
**Estela Carvalho**	estellacarvalho@gmail.com	Safety Medical Doctor
**Telma Goldenberg**	telmagold@gmail.com	Safety Medical Doctor
**Simone Collopy**	sicollopy@gmail.com	Safety Medical Doctor
**Girlene Pandine**	gspandine@gmail.com	Phlebotomist
**Rafaela Silva**	rafaelaenf@yahoo.com.br	Phlebotomist
**Daniella Mesquita**	danielle.mesquita@gmail.com	Phlebotomist
**Cíntia Maria Lopes Alves**	cintiamarialopes9@gmail.com	Phlebotomist
**Ana Rita Lopes Souza**	nanalopes_18@hotmail.com	Call Operator
**Marilena Oliveira**	marilena_pires@hotmail.com	Call Operator
**Cíntia Lopes Bogéa**	bogeacintia@gmail.com	Phlebotomist
**Marciléia Soares D.Allão Chaves**	marcileia.enf@gmail.com	Study Nurse
**Ayla Alcoforado da Silva dos Santos**	aylauanny@gmail.com	Study Nurse
**Renato da Costa Silva**	renatocostta1212@gmail.com	Study Nurse
**Marilda Siqueira**	mmsiq@ioc.fiocruz.br	Virology Laboratory Lead

BRACE trial Consortium Group List_29MAR2021

BRAZIL – MANAUS

Name	Email	Role
**Manaus**		
**Marcus Lacerda**	marcuslacerda.br@gmail.com	Principal Investigator (Manaus)
**Bruno Jardim**	brunojardim89@hotmail.com	Co-Principal Investigator
**Mariana Simão**	marianasimaoxavier@gmail.com	Study Coordinator
**Fernando Val**	ffaval@gmail.com	Study Coordinator
**Larissa Brasil**	larissa_brasil@hotmail.com	Laboratory Head
**Christiane Prado**	christianeprado95@gmail.com	Nurse Leader
**Kelry Mazurega**	kelryoliveira27@gmail.com	Pharmacist Leader
**Vanderson Sampaio**	vandersons@gmail.com	Data Manager
**Anna Gabriela Santos**	annagabrielarezende@gmail.com	Call Center Head
**Tyane Jardim**	tyane_almeida@hotmail.com	Safety Medical Doctor
**Bernardo Maia**	bernardo.mpesq88@gmail.com	Data Assistant
**Ariandra Sartim**	ariandrag@hotmail.com	Pharmacist
**Alexandre Trindade**	atrindade15@gmail.com	Pharmacist
**Rosangela Melo**	tanantasmelo@gmail.com	Pharmacist
**Arthur Otsuka**	arthurfarmaceutico@outlook.com	Pharmacist
**Dayanne Barros**	dayanne.barroslk@gmail.com	Pharmacist
**Ana Carolina Furtado**	acazevedofurtado@gmail.com	Call Operator
**Rayssa Paes**	Rayssapaesv2@gmail.com	Call Operator
**Ramon Castro**	rmnpeixoto@outlook.com	Call Operator
**Ana Greyce Capella**	Ana.g.capella@gmail.com	Call Operator
**Daniel Santos**	elguedes07@gmail.com	Call Operator
**Erlane Costa**	erlanesantiago72854@gmail.com	Call Operator
**Larissa Gama**	Larissamotag@gmail.com	Call Operator

Pittet LF, *et al. BMJ Open* 2023; 11:e052101. doi: 10.1136/bmjopen-2021-052101

**Maria Gabriela Oliveira**	Mgvdo.enf16@uea.edu.br	Call Operator
**Thamires Freitas**	mesquita.thamires24@gmail.com	Call Operator
**Antonny Sousa**	michaelantonny@gmail.com	Data quality control
**Thais Oliveira**	tgo.enf19@gmail.com	Nurse
**Juliana Silva**	juliananssilva22@gmail.com	Nurse
**Adria Vasconcelos**	adria.lemos22@gmail.com	Nurse
**Joel Junior**	joeljuniorifpa@gmail.com	Nurse
**Elizandra Nascimento**	eliizandrafn@gmail.com	Nurse
**Tilza Santos**	thilzadepaula@gmail.com	Nurse
**Evelyn Queiroz**	Evelyn.queiroz24@hotmail.com	Nurse
**Handerson Pereira**	handersonsilva13@hotmail.com	Nurse
**Laleyska Rodrigues**	Laleyska@gmail.com	Nurse
**Fabiane Bianca Barbosa**	bianca.albar11@gmail.com	Laboratory
**Juliana Neves**	neves.juh.costa@gmail.com	Laboratory
**Emanuelle Silva**	emanuellelira96@gmail.com	Laboratory
**Adriana Marins**	dricka.ferreira16@hotmail.com	Laboratory
**Bebeto Rodrigues**	b.rodrigues1403@gmail.com	Laboratory
**Paulo Henrique Andrade**	andrade.biomedi@gmail.com	Laboratory
**Ingrid Oliveira**	ingrid.igor30@gmail.com	Laboratory
**Gabrielle Pereira**	gabriellepereira61@gmail.com	Laboratory
**Vanessa Godinho**	vanessa.vk.castro@gmail.com	Laboratory

BRACE trial Consortium Group List_29MAR2021

ClinicalTrials.gov PRS
Protocol Registration and Results System

ClinicalTrials.gov PRS DRAFT Receipt (Working Version)
Last Update: 02/02/2021 19:11

ClinicalTrials.gov ID: NCT04327206

Study Identification

Unique Protocol ID: 62586

Brief Title: BCG Vaccination to Protect Healthcare Workers Against COVID-19 (BRACE)

Official Title: BCG Vaccination to Reduce the Impact of COVID-19 in Healthcare Workers (BRACE) Trial

Secondary IDs: U1111-1256-4104 [Registry ID: The Universal Trial Number (UTN)]

Study Status

Record Verification: February 2021

Overall Status: Recruiting

Study Start: March 30, 2020 [Actual]

Primary Completion: June 30, 2021 [Anticipated]

Study Completion: March 30, 2022 [Anticipated]

Sponsor/Collaborators

Sponsor: Murdoch Childrens Research Institute

Responsible Party: Sponsor

Collaborators: Royal Children's Hospital

Oversight

U.S. FDA-regulated Drug: No

U.S. FDA-regulated Device: No

U.S. FDA IND/IDE: No

Human Subjects Review: Board Status: Approved
Approval Number: HREC 62586
Board Name: Royal Children's Hospital Human Research Ethics Committee
Board Affiliation: Royal Children's Hospital
Phone: 03 9345 4494
Email: rch.ethics@rch.org.au
Address:

50 Flemington Road, Parkville, VIC 3052

Data Monitoring:

Study Description

Brief Summary: Phase III, two-group multicentre, randomised controlled trial in up to 10 078 healthcare workers to determine if BCG vaccination reduces the incidence and severity of COVID-19 during the 2020 pandemic.

Detailed Description: Healthcare workers are at the frontline of the coronavirus disease (COVID-19) pandemic. They will be randomised to receive a single dose of BCG vaccine or 0.9% NaCl placebo. Participants will be followed-up for 12 months with notification from a Smartphone application or phone calls (up to daily when ill) and surveys to identify and detail COVID-19 infection. Additional information on severe disease will be obtained from hospital medical records and/or government databases. Blood samples will be collected prior to randomisation and at 3, 6, 9 and 12 months to determine exposure to severe acute respiratory syndrome coronavirus 2 (SARS-CoV-2). Where required, swab/blood samples will be taken at illness episodes to assess SARS-CoV-2 infection.

The trial includes a pre-planned meta-analysis with data from 2834 participants recruited in the first phase of this study, where participants were randomised to receive BCG or no BCG vaccine at the time of receiving influenza vaccination.

Conditions

Conditions: Coronavirus Disease 2019 (COVID-19)
Respiratory Illness
Corona Virus Infection
COVID-19

Keywords:

Study Design

Study Type: Interventional

Primary Purpose: Prevention

Study Phase: Phase 3

Interventional Study Model: Parallel Assignment
Phase III, two group, multicentre, randomised controlled trial

Number of Arms: 2

Masking: Double (Participant, Outcomes Assessor)
The control group will receive a placebo of 0.9% sodium chloride (NaCl). Members of the research team doing the follow-up of participants and analysis will be blinded to the group allocation (by the removal of this variable and all other variables related to BCG from the dataset) until the formal detailed statistical analysis plan is confirmed and signed by all investigators and all data cleaning/preparation is complete.

Allocation: Randomized

Enrollment: 10078 [Anticipated]

Arms and Interventions

Arms	Assigned Interventions
Experimental: BCG vaccine	Drug: BCG Vaccine

Arms	Assigned Interventions
Participants will receive a single dose of BCG vaccine (BCG-Denmark). The adult dose of BCG vaccine is 0.1 mL injected intradermally over the distal insertion of the deltoid muscle onto the humerus (approximately one third down the upper arm).	Freeze-dried powder: Live attenuated strain of Mycobacterium bovis (BCG), Danish strain 1331. Each 0.1 ml vaccine contains between 200000 to 800000 colony forming units. Adult dose is 0.1 ml given by intradermal injection<br>Other Names:<br>• Bacille Calmette-Guerin Vaccine<br>• Bacillus Calmette-Guerin Vaccine<br>• Statens Serum Institute BCG vaccine<br>• Mycobacterium bovis BCG (Bacille Calmette Guérin), Danish Strain 1331<br>• BCG Denmark
Placebo Comparator: 0.9% Saline<br>Participants will receive a single 0.1 mL dose of 0.9%NaCl injected intradermally over the distal insertion of the deltoid muscle onto the humerus (approximately one third down the upper arm).	Drug: 0.9%NaCl<br>0.9% Sodium Chloride Injection<br>Other Names:<br>• 0.9% Saline

Outcome Measures

Primary Outcome Measure:

1. COVID-19 disease incidence
   Number of participants with COVID-19 disease defined as
   - positive SARS-Cov-2 test (PCR, antigen or serology), plus
   - fever (using self-reported questionnaire), or
   - at least one sign or symptom of respiratory disease including cough, shortness of breath, respiratory distress/ failure (using self-reported questionnaire)

   [Time Frame: Measured over the 6 months following randomisation]
2. Severe COVID-19 disease incidence
   Number of participants with severe COVID-19 disease, defined as: COVID-19 disease with hospitalisation, death, or non-hospitalised severe disease.

   Non-hospitalised severe disease is defined as non-ambulant (*) for ≥ 3 consecutive days OR unable to work (**) for ≥ 3 consecutive days.

   (*) “pretty much confined to bed (meaning finding it very difficult to do any normal daily activities”.

   (**) “I do not feel physically well enough to go to work”

   [Time Frame: Measured over the 6 months following randomisation]

Secondary Outcome Measure:

3. COVID-19 incidence by 12 months
   Number of participants with COVID-19 disease defined as
   - positive SARS-Cov-2 test (PCR or serology), plus
   - fever (using self-reported questionnaire), or
   - at least one sign or symptom of respiratory disease including cough, shortness of breath, respiratory distress/ failure (using self-reported questionnaire)

   [Time Frame: Measured over the 12 months following randomisation]
4. Severe COVID-19 incidence by 12 months
   Number of participants with severe COVID-19 disease, defined as: COVID-19 disease with hospitalisation, death, or non-hospitalised severe disease.

Pittet LF, *et al. BMJ Open* 2023; 11:e052101. doi: 10.1136/bmjopen-2021-052101

Non-hospitalised severe disease is defined as non-ambulant(*) for ≥ 3 consecutive days OR unable to work (**) for ≥ 3 consecutive days.

* “pretty much confined to bed (meaning finding it very difficult to do any normal daily activities”

** “I do not feel physically well enough to go to work”

[Time Frame: Measured over the 12 months following randomisation]

5. Time to first symptom of COVID-19
Time to first symptom of COVID-19 in a participant who subsequently meets the case definition:

    - positive SARS-Cov-2 test (PCR, antigen or serology), plus
    - fever (using self-reported questionnaire), or
    - at least one sign or symptom of respiratory disease including cough, shortness of breath, respiratory distress/failure (using self-reported questionnaire)

    [Time Frame: Measured over the 12 months following randomisation]

6. Episodes of COVID-19
Number of episodes of COVID-19 disease defined as

    - positive SARS-Cov-2 test (PCR, antigen or serology), plus
    - fever (using self-reported questionnaire), or
    - at least one sign or symptom of respiratory disease including cough, shortness of breath, respiratory distress/failure (using self-reported questionnaire)

    [Time Frame: Measured over the 12 months following randomisation]

7. Asymptomatic SARS-CoV-2 infection
Number of participants with asymptomatic SARS-CoV-2 infection defined as

    - Evidence of SARS-CoV-2 infection (by PCR or seroconversion)
    - Absence of respiratory illness (using self-reported questionnaire)
    - No evidence of exposure prior to randomisation (inclusion serology negative)

    [Time Frame: Measured over the 12 months following randomisation]

8. Work absenteeism due to COVID-19
Number of days (using self-reported questionnaire) unable to work (excludes quarantine/workplace restrictions) due to COVID-19 disease defined as

    - positive SARS-Cov-2 test (PCR, antigen or serology), plus
    - fever (using self-reported questionnaire), or
    - at least one sign or symptom of respiratory disease including cough, shortness of breath, respiratory distress/failure (using self-reported questionnaire)

    [Time Frame: Measured over the 12 months following randomisation]

9. Bed confinement due to COVID-19
Number of days confined to bed (using self-reported questionnaire) due to COVID-19 disease defined as

    - positive SARS-Cov-2 test (PCR or serology), plus
    - fever (using self-reported questionnaire), or
    - at least one sign or symptom of respiratory disease including cough, shortness of breath, respiratory distress/failure (using self-reported questionnaire)

    [Time Frame: Measured over the 12 months following randomisation]

10. Symptom duration of COVID-19
Number of days with symptoms in any episode of illness that meets the case definition for COVID-19 disease:

    - positive SARS-Cov-2 test (PCR, antigen or serology), plus
    - fever (using self-reported questionnaire), or
    - at least one sign or symptom of respiratory disease including cough, shortness of breath, respiratory distress/failure (using self-reported questionnaire)

    [Time Frame: Measured over the 12 months following randomisation]

11. SARS-CoV-2 pneumonia
    Number of pneumonia cases (abnormal chest X-ray) (using self-reported questionnaire and/or medical/hospital records) associated with a positive SARS-CoV-2 test

    [Time Frame: Measured over the 12 months following randomisation]
12. Oxygen therapy with SARS-CoV-2
    Need for oxygen therapy (using self-reported questionnaire and/or medical/hospital records) associated with a positive SARS-CoV-2 test

    [Time Frame: Measured over the 12 months following randomisation]
13. Critical care admissions with SARS-CoV-2
    Number of admission to critical care (using self-reported questionnaire and/or medical/hospital records) associated with a positive SARS-CoV-2 test

    [Time Frame: Measured over the 12 months following randomisation]
14. Critical care admission duration with SARS-CoV-2
    Number of days admitted to critical care (using self-reported questionnaire and/or medical/hospital records) associated with a positive SARS-CoV-2 test

    [Time Frame: Measured over the 12 months following randomisation]
15. Mechanical ventilation with SARS-CoV-2
    Number of participants needing mechanical ventilation (using self-reported questionnaire and/or medical/hospital records) and a positive SARS-CoV-2 test

    [Time Frame: Measured over the 12 months following randomisation]
16. Mechanical ventilation duration with SARS-CoV-2
    Number of days that participants needed mechanical ventilation (using self-reported questionnaire and/or medical/hospital records) and a positive SARS-CoV-2 test

    [Time Frame: Measured over the 12 months following randomisation]
17. Hospitalisation duration with COVID-19
    Number of days of hospitalisation due to COVID-19 (using self-reported questionnaire and/or medical/hospital records).

    [Time Frame: Measured over the 12 months following randomisation]
18. Mortality with SARS-CoV-2
    Number of deaths (from death registry) associated with a positive SARS-CoV-2 test

    [Time Frame: Measured over the 12 months following randomisation]
19. Fever or respiratory illness
    Number of participants with fever or respiratory illness will be defined as:

    - fever (using self-reported questionnaire), or
    - at least one sign or symptom of respiratory disease including cough, shortness of breath, respiratory distress/failure, runny/blocked nose (using self-reported questionnaire)

    [Time Frame: Measured over the 12 months following randomisation]
20. Episodes of fever or respiratory illness
    Number of episodes of fever or respiratory illness, defined as

    - fever (using self-reported questionnaire), or
    - at least one sign or symptom of respiratory disease including cough, shortness of breath, respiratory distress/failure, runny/blocked nose (using self-reported questionnaire)

    [Time Frame: Measured over the 12 months following randomisation]
21. Work absenteeism due to fever or respiratory illness
    Number of days (using self-reported questionnaire) unable to work (excludes quarantine/workplace restrictions) due to fever or respiratory illness defined as

    - fever (using self-reported questionnaire), or

Pittet LF, *et al. BMJ Open* 2023; 11:e052101. doi: 10.1136/bmjopen-2021-052101

- at least one sign or symptom of respiratory disease including cough, shortness of breath, respiratory distress/ failure, runny/blocked nose (using self-reported questionnaire)

[Time Frame: Measured over the 12 months following randomisation]

22. Bed confinement due to fever or respiratory illness
Number of days confined to bed (using self-reported questionnaire) due to fever or respiratory illness defined as

- fever (using self-reported questionnaire), or
- at least one sign or symptom of respiratory disease including cough, shortness of breath, respiratory distress/ failure, runny/blocked nose (using self-reported questionnaire)

[Time Frame: Measured over the 12 months following randomisation]

23. Symptom duration of fever or respiratory illness
Number of days with symptoms in any episode of illness that meets the case definition for fever or respiratory illness:

- fever (using self-reported questionnaire), or
- at least one sign or symptom of respiratory disease including cough, shortness of breath, respiratory distress/ failure, runny/blocked nose (using self-reported questionnaire)

[Time Frame: Measured over the 12 months following randomisation]

24. Pneumonia
Number of pneumonia cases (abnormal chest X-ray) (using self-reported questionnaire and/or medical/hospital records)

[Time Frame: Measured over the 12 months following randomisation]

25. Oxygen therapy
Need for oxygen therapy (using self-reported questionnaire and/or medical/hospital records)

[Time Frame: Measured over the 12 months following randomisation]

26. Critical care admissions
Number of admission to critical care (using self-reported questionnaire and/or medical/hospital records)

[Time Frame: Measured over the 12 months following randomisation]

27. Mechanical ventilation
Number of participants needing mechanical ventilation (using self-reported questionnaire and/or medical/hospital records)

[Time Frame: Measured over the 12 months following randomisation]

28. Mortality
Number of deaths (from death registry)

[Time Frame: Measured over the 12 months following randomisation]

29. Hospitalisation duration with fever or respiratory illness
Number of days of hospitalisation due to fever or respiratory illness (using self-reported questionnaire, medical/hospital records and/or government registries)

[Time Frame: Measured over the 12 months following randomisation]

30. Unplanned work absenteeism
Number of days of unplanned absenteeism for any reason (using self-reported questionnaire)

[Time Frame: Measured over the 12 months following randomisation]

31. Local and systemic adverse events to BCG vaccination in healthcare workers
Type and severity of local and systemic adverse events will be collected in self-reported questionnaire and graded using toxicity grading scale.

[Time Frame: Measured over the 3 months following randomisation]

Eligibility

Minimum Age: 18 Years

Maximum Age:

Sex: All

Gender Based:

Accepts Healthy Volunteers: Yes

Criteria: Inclusion Criteria:

- Over 18 years of age
- Healthcare worker
    - This is defined as anyone who works in a healthcare setting or has face to face contact with patients.
- Provide a signed and dated informed consent form
- Australian sites only: If annual influenza vaccination is available, receiving the flu vaccine is an eligibility requirement. The flu vaccine will be required a minimum of 3 days in advance of randomisation in the BRACE trial.
- Pre-randomisation blood collected

Exclusion Criteria:

- Has any BCG vaccine contraindication
    - Fever or generalised skin infection (where feasible, randomisation can be delayed until cleared)
    - Weakened resistance toward infections due to a disease in/of the immune system
    - Receiving medical treatment that affects the immune response or other immunosuppressive therapy in the last year.
        - These therapies include systemic corticosteroids (≥20 mg for ≥2 weeks), non-biological immunosuppressant (also known as 'DMARDS'), biological agents (such as monoclonal antibodies against tumour necrosis factor (TNF)-alpha).
    - People with congenital cellular immunodeficiencies, including specific deficiencies of the interferon-gamma pathway
    - People with malignancies involving bone marrow or lymphoid systems
    - People with any serious underlying illness (such as malignancy)
        - NB: People with cardiovascular disease, hypertension, diabetes, and/or chronic respiratory disease are eligible if not immunocompromised, and if they meet other eligibility criteria
    - Known or suspected HIV infection,even if they are asymptomatic or have normal immune function.
    - This is because of the risk of disseminated BCG infection
    - People with active skin disease such as eczema, dermatitis or psoriasis at or near the site of vaccination
    - A different adjacent site on the upper arm can be chosen if necessary
    - Pregnant
        - Although there is no evidence that BCG vaccination is harmful during pregnancy, it is a contra-indication to BCG vaccination. Therefore, we will exclude women who think they could be pregnant or are planning to become pregnant within the next month.
        - UK specific: Although there is no evidence that BCG vaccination is harmful during pregnancy, it is a contra-indication to BCG

    - vaccination. Therefore, we will exclude women of childbearing potential (WOCBP) who think they could be pregnant.
    - Spain specific: If the patient is female, and of childbearing potential, she must have a negative pregnancy test at the time of inclusion and practice a reliable method of birth control for 30 days after receiving the BCG vaccination.
  - Another live vaccine administered in the month prior to randomisation
  - Require another live vaccine to be administered within the month following BCG randomisation
    - If the other live vaccine can be given on the same day, this exclusion criteria does not apply
  - Known anaphylactic reaction to any of the ingredients present in the BCG vaccine
  - Previous active TB disease
  - Currently receiving long term (more than 1 month) treatment with isoniazid, rifampicin or quinolone as these antibiotics have activity against Mycobacterium bovis
- Previous adverse reaction to BCG vaccine (significant local reaction (abscess) or suppurative lymphadenitis)
- BCG vaccine given within the last year
- Have previously had a SARS-CoV-2 positive test result (positive PCR on a respiratory sample or a positive SARS-CoV-2 diagnostic antigen test approved by the local jurisdiction's public health policy)
- Already part of this trial, recruited at a different site/hospital.
- Participation in another COVID-19 prevention trial
- Have previously received a COVID-19-specific vaccine

Contacts/Locations

Central Contact Person: Prof Nigel Curtis, MBBS PhD
Telephone: +613 93456366
Email: nigel.curtis@rch.org.au

Central Contact Backup:

Study Officials: Prof Nigel Curtis
Study Principal Investigator
Murdoch Children's Research Institute

Locations: **Australia, Victoria**
Royal Children's Hospital
[Active, not recruiting]
Melbourne, Victoria, Australia, 3052
Contact: Prof Nigel Curtis, MBBS PhD +61 3 9345 6366
nigel.curtis@mcri.edu.au
Contact: +613 9936 6042

Monash Health- Monash Medical Centre
[Active, not recruiting]
Melbourne, Victoria, Australia, 3168
Contact: A/Prof Tony Korman, MBBS FRACP +61 3 9594 4533
tony.korman@monash.edu

Epworth Richmond
[Active, not recruiting]
Melbourne, Victoria, Australia, 3121
Contact: Dr Nicole Tan, MBBS FANZCA +61 3 9427 7899
niki.tan@anaestheticservices.com.au

Australia, Western Australia
Sir Charles Gairdner Hospital
[Active, not recruiting]
Perth, Western Australia, Australia, 6009
Contact: Prof Michaela Lucas, MD PhD +61 8 6383 4311
michaela.lucas@health.wa.gov.au

Fiona Stanley Hospital
[Active, not recruiting]
Murdoch, Western Australia, Australia, 6150
Contact: Dr Laurens Manning, MBChB PhD +61 8 6152 2222
laurens.manning@health.wa.gov.au

Perth Children's Hospital
[Active, not recruiting]
Perth, Western Australia, Australia, 6009
Contact: Prof Peter Richmond, MBBS FRACP +61 8 6456 5604
peter.richmond@uwa.edu.au

Australia, South Australia
Women's and Children's Hospital
[Active, not recruiting]
North Adelaide, South Australia, Australia, 5006
Contact: Prof Helen Marshall, MBBS MD MPH +61 8 8161 8115
helen.marshall@adelaide.edu.au

Royal Adelaide Hospital
[Active, not recruiting]
Adelaide, South Australia, Australia, 5000
Contact: Dr Simone Barry, MBBS PhD +61 8 7074 0000
simone.barry@sa.gov.au

Australia, New South Wales
Prince of Wales Hospital
[Active, not recruiting]
Sydney, New South Wales, Australia, 2031
Contact: A/Prof Jeffrey Post, MBBS PhD +61 2 93823405

The Children's Hospital at Westmead
[Active, not recruiting]
Sydney, New South Wales, Australia, 2145
Contact: A/Prof Nicholas Wood, MBBS PhD +61 2 9845 0000
nicholas.wood@health.nsw.gov.au

Sydney Children's Hospital, Randwick
[Active, not recruiting]
Sydney, New South Wales, Australia, 2145
Contact: Dr Brendan McMullan, BMed, FRACP +61 2 9382 1111
brendan.mcmullan@health.nsw.gov.au

Westmead Hospital
[Active, not recruiting]
Sydney, New South Wales, Australia, 2145
Contact: A/Prof Mark Douglas, MBBS PhD +61 2 8890 6012
mark.douglas@sydney.edu.au

St Vincent's Hospital, Sydney
[Active, not recruiting]
Sydney, New South Wales, Australia, 2010

Pittet LF, *et al. BMJ Open* 2023; 11:e052101. doi: 10.1136/bmjopen-2021-052101

Contact: Dr Anthony Byrne, MBBS PhD +61 2 8382 1111
anthony.byrne@svha.org.au

Netherlands
University hospital in Utrecht (UMCU)
[Active, not recruiting]
Utrecht, Netherlands, 3584 CX
Contact: Prof Marc Bonten, MD PhD +31 88-755 0350
m.j.m.bonten@umcutrecht.nl

Amphia Hospital
[Active, not recruiting]
Breda, Netherlands, 4818 CK
Contact: Prof Jan Kluytmans, MD PhD +31 6-533 854 67/003
jankluytmans@gmail.com

Rijnstate Hospital
[Active, not recruiting]
Arnhem, Netherlands, 6815 AD
Contact: Dr Jet Gisolf, MD PhD +31 88-005 6735 JGisolf@rijnstate.nl

Noord West Ziekenhuis
[Active, not recruiting]
Alkmaar, Netherlands, 1815 JD
Contact: Dr Wim Boersma, MD PhD +31 72-548 2700 w.boersma@nwz.nl

Radboud UMC
[Active, not recruiting]
Nijmegen, Netherlands, 6525 GA
Contact: Dr Jaap ten Oever, MD PhD +31 24-361 7257
Jaap.tenOever@radboudumc.nl

St Antonius Hospital
[Active, not recruiting]
Nieuwegein, Netherlands, 3435 CM
Contact: Dr Bob Meek, MSc PhD +31 88-320 7413
b.meek@antoniusziekenhuis.nl

Spain
Mutua Terrassa Univeristy Hospital
[Recruiting]
Terrassa, Barcelona, Spain, 08221
Contact: Dr Tomás Perez Porcuna, MD PhD +34 644460736
tomasperez@mutuaterrassa.es

University Hospital German Trias I Pujol
[Active, not recruiting]
Badalona, Barcelona, Spain, 08916
Contact: Dr Antoni Rosell, MD PhD +34934583561/639352383
arosellg.germanstrias@gencat.cat

University Hospital Cruces
[Active, not recruiting]
Barakaldo, Bizkaia, Spain, 48903
Contact: Dr Josune Goikoetxea, MD PhD +34946006000 Ext. 2330
ANEJOSUNE.GOIKOETXEAAGIRRE@osakidetza.eus

Marqués de Valdecilla University Hospital
[Active, not recruiting]
Santander, Spain, 39008

Contact: Dr María Carmen Fariñ Álvarez, MD PhD
+34677 984 594/942 31 55 42 mcarmen.farinas@scsalud.es

University Hospital Virgen Macarena
[Active, not recruiting]
Sevilla, Spain, 41009
Contact: Prof Jesús Rodríguez-Baño, MD PhD +34671592434
jesusrb@us.es

United Kingdom
St Leonard's Practice
[Active, not recruiting]
St Leonards, Exeter, United Kingdom, EX1 1SB
Contact: Alex Harding +44 01392 201790 a.m.harding@nhs.net

Ide Lane Surgery
[Active, not recruiting]
Alphington, Exeter, United Kingdom, EX2 8UP
Contact: Daniel Webber-Rookes +44 01392 439868 d.webber-rookers@nhs.net

Travel Clinic
[Active, not recruiting]
Exeter, Exeter, United Kingdom, EX1 1PR
Contact: James Moore +44 01392 430590
james@travelhealthconsultancy.co.uk

Brazil
Federal University of Mato Grosso do Sul
[Recruiting]
Campo Grande, Mato Grosso Do Sul, Brazil, 79070-900
Contact: Júlio Croda, MD PhD 55 67 981229959 julio.croda@fiocruz.br

Hospital Regional de Mato Grosso do Sul
[Recruiting]
Campo Grande, Mato Grosso Do Sul, Brazil, 79084-180
Contact: Júlio Croda, MD PhD 55 67 981229959 julio.croda@fiocruz.br

CASSEMS Hospital
[Recruiting]
Campo Grande, Mato Grosso Do Sul, Brazil, 79002-251
Contact: Júlio Croda, MD PhD 55 67 981229959 julio.croda@fiocruz.br

Santa Casa Hospital
[Recruiting]
Campo Grande, Mato Grosso Do Sul, Brazil, 79002-230
Contact: Júlio Croda, MD PhD 55 67 981229959 julio.croda@fiocruz.br

Centro de Referência Prof Hélio Fraga
[Recruiting]
Rio de Janeiro, RJ, Brazil, 22780-195
Contact: Margareth Dalcolmo, MD PhD 55 21 999894904
margarethdalcolmo@ensp.fiocruz.br

Centro de Estudos da Saúde do Trabalhador e Ecologia Humana
[Recruiting]
Rio de Janeiro, RJ, Brazil, 22780-195
Contact: Margareth Dalcolmo, MD PhD 55 21 999894904
margarethdalcolmo@ensp.fiocruz.br

Pittet LF, *et al. BMJ Open* 2023; 11:e052101. doi: 10.1136/bmjopen-2021-052101

Fundação de Medicina Tropical Dr Heitor Vieira Dourado (FMT-HVD) [Recruiting]
Manaus, Amazonas, Brazil, 69040-000
Contact: Marcus Lacerda, MD PhD 55 92 991147633
marcuslacerda.br@gmail.com

IPDSharing

Plan to Share IPD: Yes

Beginning 6 months following analysis and article publications, the following may be made available long-term for use by future researchers from a recognised research institution whose proposed use of the data has been ethically reviewed and approved by an independent committee and who accept MCRI's conditions, under a collaborator agreement, for accessing:

- Individual participant data that underlie the results reported in our articles after de-identification (text, tables, figures and appendices)
- Study protocol, Statistical Analysis Plan, Participant Informed Consent Form (PICF)

Supporting Information:
Study Protocol
Statistical Analysis Plan (SAP)
Informed Consent Form (ICF)

Time Frame:
Beginning 6 months following analysis and article publications, for long-term use

Access Criteria:
Researchers from a recognised research institution whose proposed use of the data has been ethically reviewed and approved by an independent committee and who accept MCRI's conditions, under a collaborator agreement

URL:

References

Citations:

Links:

Available IPD/Information:

Documents

Study Protocol and Statistical Analysis Plan
Document Date: December 10, 2020
Uploaded: 02/01/2021 01:43

U.S. National Library of Medicine | U.S. National Institutes of Health | U.S. Department of Health & Human Services

Insert Header with institution's name or institution's letterhead

Participant Information Sheet/Consent Form

Interventional Study - *Adult providing own consent*

[Insert site name]

**Title**	BCG vaccination to Reduce the impact of COVID-19 in healthcare workers (BRACE) Trial
**Short Title**	BRACE
**Protocol Number**	HREC number 62586
**Trial Sponsor**	Murdoch Children's Research Institute (MCRI)
**Chief Principal Investigator/ Principal Investigator**	Prof Nigel Curtis */ Principal Investigator]*
**Location** *(where CPI/PI will recruit)*	*[Location]*

1 Introduction

We are inviting you to take part in this trial because you are a healthcare worker. This trial is testing whether the Bacille Calmette-Guerin (BCG) vaccine can help reduce the severity of COVID-19 in healthcare workers.

This Participant Information Sheet/Consent Form tells you about the trial. It explains the tests and treatments involved. Knowing what is involved will help you decide if you want to take part in the trial.

Please read this information carefully. Ask questions about anything that you don't understand or want to know more about. Before deciding whether or not to take part, you might want to talk about it with a relative, friend or your local doctor.

Participation in this trial is voluntary. If you don't wish to take part, you don't have to. You will receive the best possible care whether or not you take part.

If you decide you want to take part in the trial, you will be asked to sign the consent section. By signing it you are telling us that you:

- understand what you have read
- consent to take part in the trial
- consent to have the tests and treatments that are described
- consent to the use of your personal and health information as described.

We will give you a copy of this Participant Information and Consent Form to keep.

If you want more information or wish to speak to a study team member before providing your consent, please contact:
[study team contact information]

2 What is the purpose of this trial?

The severe acute respiratory syndrome-coronavirus 2 (SARS-Cov-2) is a coronavirus that emerged in China in December 2019. It is predicted that up to 60% of the population could become infected. There have been already over 18,000,000 cases of coronavirus disease (COVID-19) and greater than 690,000 deaths globally (as of 04 Aug 2020). For around 80% of people, the virus causes mild to moderate disease with symptoms similar to common respiratory diseases such as influenza, including fever, cough, and fatigue. In around 14% of people, the disease causes severe disease that requires hospitalisation. The remaining 6% are critical cases that have respiratory failure, septic shock and/or organ failure.

Healthcare workers are at the frontline of the COVID-19 pandemic. Because healthcare workers work closely with patients they have greater exposure and possibly greater risk of contracting the virus. There is currently no vaccine for COVID-19, so protection of healthcare workers relies on the use of personal protective equipment. When healthcare workers are sick and unable to come to work, this puts extra pressure on the healthcare system. All hospital staff, including doctors, nurses, cleaners and administrative staff are vital to ensuring the hospital can function during a pandemic of this scale. It is vital that the hospitals don't lose a significant portion of their workforce due to illness.

The tuberculosis (TB) vaccine, Bacillus Calmette Guérin (BCG), has been shown to protect against non-TB infections by boosting the immune system. Studies show that it can decrease mortality of those infected by half and protects against other infectious diseases and improves the response to other vaccines. The mechanism by which BCG influences immunity is not completely understood

We want to find out whether the BCG vaccine might protect against COVID-19. We are interested to know if the vaccine can reduce the number of cases of COVID-19, and the severity of the illness caused by the virus, compared to a placebo.

The BCG vaccine is approved in [include country] to protect against tuberculosis. However, it is not approved to protect against other infections, such as COVID-19. This study is an experimental use of this vaccine.

The results of this trial will help us find out whether, in future novel disease outbreaks, BCG vaccination could be used as an early intervention to protect healthcare workers and high-risk groups.

You can be in the study whether or not you have had the BCG vaccine in the past.

This research has been initiated by Professor Nigel Curtis, Head of Infectious Diseases at The Royal Children's Hospital Melbourne (RCH), Leader of Infectious Diseases Group at Murdoch Children's Research Institute and Professor of Paediatric Infectious Diseases, Department of Paediatrics, The University of Melbourne.

<u>Who is involved in this trial?</u>
This trial is being led by the Murdoch Children's Research institute and will take place across multiple centres. There will be multiple sites across Australia, Europe and Latin America.

We hope to have 10078 healthcare workers in total be a part of this trial.

3 What does participation in this trial involve?

<site specific inclusion during influenza season> Because of the way this trial is designed, you must have received the current seasonal influenza vaccination to be in the trial (at least 3 days or more prior to your first study visit). We hope receiving the influenza vaccine will reduce the number

of non-COVID-19 respiratory illness, and lessen the risk of being co-infected with COVID-19 and influenza. It also means that any effect of the BCG vaccine will not be changed by participants having the flu vaccine after joining the study.>

You have already answered some screening questions that have determined that you may be eligible to be in this trial.

You will have a chance to consider the information in this form and discuss it with your family, friends or doctor. You can contact us for more information (see Section 20). We will ask you to provide your written consent when you have decided you are happy to participate.

If you agree to be in this trial, we will ask you to fill in some questions about yourself and your health. This will include your date of birth, name and other identifying details. We will ask you to complete a baseline questionnaire on whether you have had other vaccines recently, any other medical conditions you may have, your general health and lifestyle habits, and whether you have had the BCG vaccine before.

Once you have completed the questionnaire, you will come to get your vaccine. You can come at [any time/specified times of day]. [Sites to include information here about bookings, if required].

We will confirm that you have signed the consent form, filled out the baseline questionnaire and will ask you the screening questions again.

Because of the way this study is designed, even if you have provided consent, we may already have enough people in the trial when you come for your enrolment visit. If this is the case, we will tell you and you will not be put in the trial.

Pregnant healthcare workers will not be eligible to participate in this trial. Although BCG vaccination has not been shown to be harmful during pregnancy, the use of live vaccines (such as BCG) during these times is contra-indicated. Therefore, if you are pregnant, planning to fall pregnant within a month of enrolment in this trial, you will not be allowed to participate in this trial. If you think you could be pregnant we will ask you to do a pregnancy test prior to taking part. We will have pregnancy tests available when you come for enrolment if you would like to check on the day or to take away to self-test before enrolment.

You cannot take part in this trial if you are receiving medical treatment that affects the immune response (or other immunosuppressive therapy), have a serious underlying medical illness, have received any live vaccine in the past month or BCG vaccine in the past year.

Once we have confirmed that you are able to be part of the trial, the study team member will collect a blood sample of up to 30 mL <Brazil: 35ml>. This will be used to check whether you have already been exposed to COVID-19 before being in this trial and to look at the changes the vaccines make to your immune system. We will not have these results until the end of the study.

This is a randomised controlled research project. Sometimes we do not know which treatment is best for treating a condition. To find out we need to compare different treatments. We put people into groups and give each group a different treatment. The results are compared to see if one is better. To try to make sure the groups are the same, each participant is put into a group by chance (random).

In this trial we will put you into one of two groups:

- Intervention group 1 – You will be given a placebo vaccine. A placebo looks like the real thing but contains no active ingredients.
- Intervention group 2 – You will be given the BCG vaccine.

The chance of being in each group is 1 in 2, or 50%. You will not know which group you are in until the end of the trial. In an emergency, the study staff can find out which group you were in if this information is needed.

If you consent to being in this trial you are agreeing that you are happy to be in either group and to not knowing which group you are in.

After we have collected the blood sample, you will be randomly allocated to one of the two intervention groups. If we are unable to collect your blood, you cannot take part in this trial and will not be randomised.

A trial team member will administer your BCG vaccine or placebo in the arm. Once you have had your vaccine, you will need to stay in the hospital or clinic for 20 minutes, as per usual.

We will ask you to complete a questionnaire 2 weeks after your vaccination to tell us about your reaction to the vaccination (BCG or placebo). We will ask you about your vaccination site, and give you the option to send us a photograph of the vaccination site (using your smartphone).

We would like you to complete a survey about any time that you are unwell with a fever (temperature over 38˚C) or with any respiratory symptom (sore throat, cough, difficulty breathing). We expect the survey will take no longer than 2-minutes each day you are unwell. <locations using app only: You will be able to access the survey at any time using a phone app. Every week for the 12 months of the trial we will send a reminder, asking if you have had a fever or respiratory symptoms since the last time you responded.> If you haven't been unwell (with fever or respiratory symptom), all you will need to do is respond by saying 'no'. If you have been unwell you will respond with 'yes' and complete the survey.

If you report symptoms of respiratory illness or fever during the 12 months of this trial, we want to confirm whether you have COVID-19 or not. If you have these symptoms you should have a test done through a centralised service, and we will get these results. In rare circumstances, home visits or self-swabbing kits may be required to ensure access to COVID-19 testing. <locations using app only: The app you use to log your symptoms will prompt you to get a test if required.>

At 3, 6, 9 and 12 months after your enrolment, we will send you a longer questionnaire asking about your exposure to COVID-19 and any medical interventions you may have had.
To the best of your memory, we will also request you to confirm the main episodes of illness experienced in the prior 3 months. We will also ask for information on the vaccination site of the BCG or placebo, and if you have a wound, how your arm has healed.

Approximately 3, 6, 9 and 12 months after your enrolment in the trial, you will attend a study visit and the trial staff will collect a blood sample of up to 30mL. This will be tested to see if you had a COVID-19 infection without having symptoms and to look at the changes the vaccines made to your immune system. <country/site specific: The blood collections at 6 months and 9 months may be done by self-administered finger prick blood spots with kits provided to you by the study team. This means you could collect the sample at home yourself instead of at a site study visit. If this is your preferred way of providing your sample we will also ask you for your home address so that we can mail the in-home collection kits to you.>

<Locations using third party providers for messages/booking system> Mobile messages and managing study appointment

<Locations using message> As a participant in the study you will receive messages from the study team. You may receive these messages via a third party communications platform used by the study team.

You may need to use an online appointment scheduling platform to book study visit appointments. You may be required to login to book and manage your appointment time for your clinic visit.

To enable you to <location using message> receive messages and to manage your study appointments, some limited personal information (such as your name, mobile phone number and email) may be transferred to the vendors of the third party platforms. The vendor may be located

locally or in another country. The platforms used by the study team have been carefully chosen so that your personal information will be stored securely and processed only in accordance with applicable data protection and privacy laws and regulations. The vendors of the relevant platforms are not permitted to share your personal information with any third parties, and may use your personal information solely to communicate with you regarding the study.

Collection of Hospital data
In addition, we will obtain details about your health from <insert name of government body who holds the hospital level data> who collects information about presentations to hospitals and emergency departments for medical care in <insert state name>.

Collecting this information will help us to determine if the BCG reduces the likelihood of getting admitted to hospital, whether it is cost effective and will help us measure the outcomes at the end of the study.

For us to obtain details from <insert name of government body who holds the hospital level data>, we will require you to complete the consent form authorising the study to access your complete hospital records.

The specific health data we would like to obtain from <insert name of government body who holds the hospital level data> is for 12 months from the time you consent to the study. It will include details of your hospitalisations and emergency department visits such as diagnosis, length of stay and its costs.

This data collection within the trial has been approved by the Human Research Ethics Committee at the Royal Children's Hospital. With your consent, we will provide your identifying information (your name, address, date of birth, country of birth and <country specific detail ie. Medicare care number>) to <insert name of government body who holds the data>. Based on only this identifying information, these organisations will identify the health related data they hold about you and release to the trial researchers only information that is consistent with the aims of this research project.

Information about how your data will be protected is in section 16 of this form.

Collection of data on herpes simplex recurrences (exploratory objective)
As BCG could also help to prevent other viral infection, we will ask you whether you have recurrent herpetic infection (such as cold sores on the lips). This will be asked at enrolment and in the questionnaires at 3, 6, 9 and 12 months after your enrolment.

OPTIONAL CONSENT – Contact for future research
Because you have been involved in this trial, there may be future studies for which you are eligible. Should this occur, we would like to contact you to find out if you are interested in participating. If you agree to this, please tick the box on the final page of this form.

OPTIONAL CONSENT – Biobanking of Samples
We are asking you to consider allowing us to store any remaining samples and data at the end of this trial for use in future research relating to immunology, vaccines or infectious diseases.

Samples would be stored, labelled with a code, at MCRI laboratories (Infectious Diseases Group) in Melbourne.

For tests that require equipment or technical expertise not available in Melbourne, select specimens may be sent to collaborating laboratories outside of Melbourne (interstate and/or overseas) for further testing.

Any research conducted with your samples will be approved by a Human Research Ethics Committee. We do not plan to contact you for your permission to conduct this future research.

If you agree to this, please tick the box on the final page of this form.

OPTIONAL CONSENT – Genetic analysis

Our bodies are made up of different types of cells. Inside these cells you find genes. Genes are passed down in families from parents to children: you get half your genes from your mother and half from your father. Our genes contain all the information that makes us what we are, including our eye colour, blood type, and height and whether we are born as a boy or a girl.

There are about 23,000 genes that make up a human being and genes are arranged along a chemical substance called DNA. If you provide consent for genetic analysis we will extract DNA from your blood sample. We will look to see if there are genetic features in your DNA that might be associated with COVID-19 responses, how your immune system functions, how the vaccinations changed your immune responses, and whether they alter the ability for BCG to protect against COVID-19.

The genetic analysis that we are doing is for research purposes only and the significance of the results are unknown, therefore we will not provide individual results to you.

This part of our study is voluntary, if you agree to this, please tick the box on the final page of this form.

<Australian sites: optional inclusion

OPTIONAL CONSENT – stool sample collection for microbiome analysis

The gut microbiome refers to the types and relative amounts of different bacteria and organisms that are found in the gut. Many previous studies have shown that the gut microbiome can have strong influences on immune responses in the body including, potentially, immune responses to vaccination.

If you provide consent for stool sample collection, we will provide you with a collection kit for you to take home and you will be able to return the sample in the mail. There will be no financial cost to you to do this as we will provide you with everything you need to collect the sample and a postage-paid envelope to return the sample. We will then extract DNA from your stool sample and we will determine the abundance of microbes (and the genes they encode) in your sample and investigate whether the gut microbiome is associated with immune responses to the BCG vaccine or any of the other outcomes being measured in the trial.

The microbiome analysis that we are doing is for research purposes only and the significance of the results are unknown, therefore we will not provide individual results to you.

This part of our study is voluntary, if you agree to this, please tick the box on the final page of this form.>

<site specific: OPTIONAL CONSENT – additional biological sample during episode of illness

We can learn more about COVID-19 infections and how BCG might help to protect against or reduce the severity of COVID-19 by collecting biological samples such as blood and saliva/respiratory swabs from people with the infection. This will help us to answer important questions including: What does the immune response to COVID-19 look like? Why do some people have more severe COVID-19 illnesses than others? How does BCG change the way your body responds to COVID-19 and other infections?

If you provide consent for additional biological sample collection during an episode of illness, a trained member of the study team may take a blood sample (up to 30mL) and saliva/respiratory swab/s from you during or up to one month after resolution of an episode of illness with fever or respiratory symptoms.

The sample collection will be done by trained staff at a study site or at your home. We will aim to take these samples at the same time as any other clinical or research samples where possible to minimise the number of tests for you.

This part of our study is voluntary, if you agree to this, please tick the box on the final page of this form.>

4 What do I have to do?

You will need to:

- Complete a diary questionnaire about your vaccination site and any local reaction you have. The questionnaire will include the option to send us a photograph of your vaccine site (taken with your smartphone)
- Fill out a questionnaire each time you are unwell with a fever or respiratory symptoms during the study <country specific: using a smartphone application designed for the trial or via phone calls>
- Complete 4 longer questionnaires (approximately 10 minutes) 3, 6, 9 and 12 months after your enrolment
- Reply to a weekly prompt from <country specific: the study app or via phone >with yes/no as to whether if you have not been unwell with a fever or respiratory symptoms. If we don't hear from you we will send an email reminder and may also phone you.
- Undergo respiratory swab testing for COVID-19 on each occasion you have any symptoms consistent with this infection
- Attend a study visit for randomisation, vaccination and blood collection, and four follow-up study visits for blood collection. <country/site specific: The blood collections at 6 months and 9 months may be done by self-administered finger prick blood spots that you return/post to the study site instead of study visits>

5 Other relevant information about the trial

We will not tell the hospital that you work for which of their staff members have consented, refused or were ineligible to participate in this trial.

There are no costs associated with participating in this trial, nor will you be paid. All medication, tests and medical care required as part of the trial will be provided to you free of charge.

Some research studies do not allow participants to be in two studies. We allow this but other studies may not. If you participate in this trial you will not be able to participate in trials of other preventative measures for COVID-19.

6 Do I have to take part in this trial?

Participation in any research project is voluntary. If you do not wish to take part, you do not have to. If you decide to take part and later change your mind, you are free to withdraw from the trial at any stage.

If you do decide to take part, you will need to sign this Participant Information and Consent Form. We will give you a copy to keep.

Your decision whether to take part or not to take part, or to take part and then withdraw, will not affect your relationship with [Institution].

7 What are the alternatives to participation?

If you decide not to be in this trial you can possibly take part in other trials testing other preventive interventions.

8 What are the possible benefits of taking part?

We cannot guarantee or promise that you will receive any benefits from this trial. However, we hope that the BCG vaccine may boost your immune system. It may provide you with non-specific protection to other illnesses.

Information we collect in this trial will help to inform how we respond to outbreaks of new diseases in the future.

9 What are the possible risks and disadvantages of taking part?

BCG is one of the most widely used vaccines in the world with an established safety record. It has been given to children since the 1920s. Most vaccines are injected into muscle, BCG is a little different as it is given just under the skin (into the 'intradermal' layer) of the left upper arm. BCG immunisation hurts a little, but this is minimised when given by experienced immunisation staff such as those who will be performing the procedure in this study.

The usual expected reaction to BCG vaccination is redness and/or a small 'papule' (a pimple or lump) at the injection site that appears weeks to months after vaccination. A few weeks later, the papule usually softens and breaks down to a small ulcer (an open sore - usually less than 15 mm in diameter). The ulcer is painless and may last from weeks to months. Once the ulcer has healed, this usually (but not always) leaves a small flat scar. Most people in Australia over the age of 50 and any that lived or travelled to a country with high levels of TB as a child, will have this scar.

Having an ulcer will not impact your ability to go to work. You can cover it with a bandage during the day while it is an open wound.

BCG vaccination can occasionally cause adverse effects, these usually get better by themselves, without requiring any specific treatment. The risk of these reactions is minimised by use of correct immunisation technique by trained staff. You may have none, some or all of the effects listed below, and they may be mild, moderate or severe. If you have any of these side effects, or are worried about them, contact us.

Participants who have had active TB in the past will be excluded. If someone has had active TB in the past, they are immune to TB so there is no indication to give BCG clinically. Because of this, there is no data available on the safety of giving BCG to people who have had active TB in the past.

Common adverse reactions:
These reactions are seen in less than 1 in 100 people immunised with BCG and usually resolve without any specific treatment:

- Abscess at the injection site or a larger ulcer
- Keloid scar at injection site (it means 'a scar thicker than usual')
- Swelling of local gland (lymph node) near the injection site (usually under the arm or near the neck)

Rare adverse reaction (less than 1 in 1000):

- Infection of the armpit lymph node, with swelling, abscess or ulcer.

Very rare adverse reaction (less than 1 in 1 million)
These conditions are usually associated with underlying inherited issues with the patient's immune system.

- Disseminated BCG infection, where the vaccine bacteria spread throughout the body or to the bone occurs in 1-4 in 1 million doses.
- Anaphylaxis (a severe allergic reaction) to the BCG vaccine has been reported only 2-3 times in the 100 years the BCG vaccine has been used.

An excessive response to the BCG vaccine may result in an ulcer with some discharge. If this happens, you should encourage the ulcer to dry and avoid abrasion (by tight clothes, for example).

Information for participants who have previously had a BCG vaccine or previous positive tuberculosis screening test (suggesting previous BCG vaccine or exposure/natural infection):
You can be in the study whether or not you have had the BCG vaccine in the past. There is no data available on the safety of giving BCG to people who have had active TB in the past. If you have had TB you should not have BCG vaccine.

If you have had a BCG vaccination previously, there is an increased risk that you may have an earlier, “accelerated” reaction which may begin within 24-48 hours of vaccination with toughening of the tissue followed by pustule formation in 5-7 days and healing within 10-15 days. Local skin lesions (ulceration and discharge) are more frequent in adults who have had a previous BCG vaccine than those who have never had BCG vaccine before. However, the risk of severe armpit lymph gland infection and disseminated BCG or reactivated tuberculosis disease has not been found to be more common in adults who have had previous BCG vaccine or positive tuberculosis screening tests.

Revaccination with the BCG as a part of this trial does not align with current vaccination guidelines, however it has been carefully considered upon systematic review of the literature to date. Adverse events will be actively monitored during the trial and medical review available for any participants who have concerns about their BCG vaccination site or scar.

Potential interaction between BCG and COVID-19 illness
Although there is a hypothetical risk that BCG vaccination could worsen the COVID-19 illness (via an exaggerated immune response) we consider this highly unlikely. We think BCG vaccine is more likely to protect against COVID-19, by reducing the severity of the illness caused by the virus. You may or may not receive any benefit from having the BCG vaccine.

Risks related to Placebo injection
Having an injection can sometimes cause very minor pain from the needle or be uncomfortable. The placebo injection will be administered by a trained immunization nurse.

Adverse effects related to blood collection and throat swabs
Having a blood sample collected may cause some discomfort or bruising. Trained members of the research team will collect these samples. Having a throat or nasal swab can sometimes be uncomfortable.

10 What will happen to my test samples?

<<Insert information relating to local storage of samples here>>

Your blood samples and throat and nasal swabs obtained for the purpose of this trial may be transferred to the Murdoch Children’s Research Institute (MCRI). They may be stored in freezers at the Infectious Diseases and Microbiology research laboratory at the MCRI until analysis. Your samples will not be sold by MCRI.

For tests that require equipment or technical expertise not available in Melbourne, select specimens may be sent to collaborating laboratories outside of Melbourne (interstate and/or overseas) for further testing. Samples that leave Australia are not protected by Australian law.

Your samples will be stored labelled with a participant code, not your name or other identifying information. Only the research team will have access to the code.

Only the members of the research team will be able to access your samples and will update reports on their location and processing. The freezers are locked and can only be opened by members of the research team who have access to the key.

11 What if new information arises during this trial?

Sometimes during the course of a trial, new information becomes available about the intervention that is being studied. In this particular case, if we happen to find that BCG is highly effective to prevent COVID-19 disease and/or severity, we will offer BCG vaccine to the participants randomised to the control group (intervention group 1). On the contrary, if BCG appears to be harmful, ie higher rates of disease and/or severity, we will alert participants in the BCG group of the greater risk which may allow them to seek alternative ways to protect themselves from getting the COVID-19 disease.

12 Can I have other treatments during this trial?

You can continue to take your regular medication during the trial.

As the BCG vaccine is live-attenuated, you should not receive any other live-attenuated vaccine (such as measles-mumps-rubella, varicella or yellow fever vaccines) in the month following your inclusion in the trial. Also you cannot receive any vaccinations in the same arm for 3 months after the vaccine is given. However, you can receive all inactivated vaccines at any time in the other arm.

While you are in this study it is important that you do not go and get the BCG vaccine elsewhere.

While you participate in this trial you may not be able to participate in new drug trials or other trials that are aimed at healthcare workers. You should not participate in trials of any other preventative measures for COVID-19 while you are participating in this trial.

13 What if I withdraw from this trial?

Withdrawing from this trial will not guarantee that you can participate in other COVID-19 related interventional trials. Once you have been enrolled in this trial you may not be eligible for other trials.

If you decide to withdraw from the trial, please notify us. This notice will allow us to discuss any health risks or special requirements linked to withdrawing. You do not have to tell us why you are withdrawing.

If you do withdraw your consent during the trial, the study doctor and relevant study staff will not collect additional personal information from you, although personal information already collected will be retained to ensure that the results of the trial can be measured properly. You should be aware that data collected by the sponsor up to the time you withdraw will form part of the trial results. If you do not want them to do this, you must tell them before you join the trial.

14 Could this trial be stopped unexpectedly?

This research project may be stopped unexpectedly for a variety of reasons. These may include reasons such as:

- Unacceptable side effects

- The BCG vaccine being shown to work and not need further testing
- Decisions made by the study team or local regulatory/health authorities.

15 What happens when the trial ends?

After 12 months, the trial will be over and we will contact you to let you know which treatment group you were in. After 12 months we will not contact you for further follow-up related to this trial.

If you have agreed, we may contact you about future research.

Part 2 How is the research project being conducted?

16 What will happen to information about me?

Data will be stored in coded/re-identifiable form which will be password protected.

The principal investigators, co investigators, study team, The Royal Children's Hospital ethics committee and biostatistician will have access to your information as identified by your allocated study number.

The collected information will be stored secure at MCRI in locked filing cabinets or in restricted access folders on the Institute's network drive and will only be accessible to the research team.

We are required to keep information collected as part of a trial for at least 15 years. The research information may be destroyed or kept indefinitely in secure storage after this time. Your information will be stored for future ethically approved research.

Any information we collect that can identify you will be treated as confidential and used only in this project unless otherwise specified. The information will be re-identifiable. This means that we will remove your name and give the information a special code number. Only the BRACE trial research team can match your name to the code number, if it is necessary to do so.

Any information obtained for the purpose of this research project that can identify you will be treated as confidential and securely stored. It will be disclosed only with your permission, or as required by law.

Information about you may be obtained from hospital records for the purposes of this research.

Your hospital information will not be reported in a way that isolates you as an individual. Results will be grouped together, summarised and not identify you in any way.

Your health records and any information obtained during the study are subject to inspection (for the purpose of verifying the procedures and the data) by the MCRI, the organisation relevant to this PICF, [organisation name] or as required by law. By signing the consent section, you authorise release of, or access to, this confidential information to the relevant study personnel and regulatory authorities as noted above.

We will present these results at scientific conferences and publish them in scientific journals.
The results will not identify any individuals, only group information will be presented. In any publication and/or presentation, information will be provided in such a way that you cannot be identified, except with your permission.

To advance science, medicine and public health, we will also need to share your de-identified data with other ethically approved research projects, data repositories, biobanks, or medical journals. When we need to do this, we will remove identifying details such as your name, date of

birth and address and give the data a special code number. Only the BRACE trial research team on this project will be able to match your name to their code number. Information that leaves Australia is not protected by Australian law.

We will put security measures in place to protect your data if and when we give it to other people.

Despite our best efforts, there is a small chance that you could be re-identified by someone outside of this research project. In the unlikely event that this happens, someone from the research team will contact you. If, at any point, you think that your may have been re-identified, please let us know.

17 Complaints and compensation

If you suffer any injuries or complications as a result of this research project, you should contact the study team as soon as possible and you will be assisted with arranging appropriate medical treatment. If you are eligible for Medicare, you can receive any medical treatment required to treat the injury or complication, free of charge, as a public patient in any Australian public hospital.

18 Who is organising and funding the research?

This trial is being funded by the Bill and Melinda Gates Foundation, [insert details of site funding] and other philanthropic organisations. No member of the research team will obtain any financial benefit from their involvement in this project (other than their ordinary wages).

This research is being conducted by a collaboration involving researchers based at hospitals globally and the Murdoch Children's Research Institute.

19 Who has reviewed the research project?

All research in Australia involving humans is reviewed by an independent group of people called a Human Research Ethics Committee (HREC). The ethical aspects of this research project have been approved by the HREC of The Royal Children's Hospital.

This project will be carried out according to the National Statement on Ethical Conduct in Human Research (2007). This statement has been developed to protect the interests of people who agree to participate in human research studies.

[insert details of ethics and governance mechanisms outside Australia as required]

20 Further information and who to contact

The person you may need to contact will depend on the nature of your query.

If you want any further information concerning this project or if you have any medical problems which may be related to your involvement in the project (for example, any side effects), you can contact the principal study doctor on *[phone number]* or any of the following people:

Clinical contact person

Position	BRACE trial program manager
Telephone	+61 409 846 988
Email	brace@mcri.edu.au

Local Site Clinical Contact Person

Name	*[Name]*
Position	*[Position]*
Telephone	*[Phone number]*
Email	*[Email address]*

For matters relating to research at the site at which you are participating, the details of the local site complaints person are:

Complaints contact person

Position	The Director, Research Ethics and Governance, The Royal Children's Hospital
Telephone	+61 3 9345 5044
Email	Rch.ethics@rch.org.au

If you have any complaints about any aspect of the project, the way it is being conducted or any questions about being a research participant in general, then you may contact:

Reviewing HREC approving this research and HREC Executive Officer details

Reviewing HREC name	The Royal Children's Hospital Human Research Ethics Committee
Telephone	+61 3 9345 5044
Email	Rch.ethics@rch.org.au

Local HREC Office contact (Single Site - Research Governance Officer)

Name	*[Name]*
Position	*[Position]*
Telephone	*[Phone number]*
Email	*[Email address]*

Pittet LF, *et al. BMJ Open* 2023; 11:e052101. doi: 10.1136/bmjopen-2021-052101

Consent Form - *Adult providing own consent*

**Title**	BCG vaccination to Reduce the impact of COVID-19 in healthcare workers (BRACE) Trial
**Short Title**	BRACE
**Protocol Number**	HREC number 62586
**Project Sponsor**	Murdoch Children's Research Institute (MCRI)
**Chief Principal Investigator/ Principal Investigator**	Prof Nigel Curtis */ Principal Investigator]*
**Location** *(where CPI/PI will recruit)*	*[Location where the research will be conducted]*

Consent Agreement

I have read the Participant Information Sheet or someone has read it to me in a language that I understand.

I understand the purposes, procedures and risks of the research described in the project.

I give permission for my doctors, other health professionals, hospitals or laboratories outside this hospital to release information to Murdoch Children's Research Institute concerning my disease and treatment for the purposes of this project. I understand that such information will remain confidential.

I have had an opportunity to ask questions and I am satisfied with the answers I have received.

I freely agree to participate in this research project as described and understand that I am free to withdraw at any time during the study without affecting my future health care.

I understand that I will be given a signed copy of this document to keep.

I understand that taking part is this trial may therefore stop me from participating in other trials that do not allow this.

OPTIONAL CONSENT:

☐ I do	☐ I do not	consent to be contacted about future ethically approved research related to this project.
☐ I do	☐ I do not	consent to my samples being placed in the biobank and used for future ethically approved research related to immunology, vaccines or infectious diseases.
☐ I do	☐ I do not	consent to genetic analysis of my samples.
☐ I do	☐ I do not	<site specific: consent to provide additional biological sample during episode of illness>
☐ I do	☐ I do not	<Australia sites optional inclusion consent to stool sample collection and microbiome analysis.>

Declaration by Participant – for participants who have read the information

Name of Participant (please print) ______________________________

Signature ______________________ Date ______________________

Pittet LF, *et al. BMJ Open* 2023; 11:e052101. doi: 10.1136/bmjopen-2021-052101

Declaration - for participants unable to read the information and consent form<br>See Note for Guidance on Good Clinical Practice CPMP/ICH/135/95 Section 4.8.9. A legally acceptable representative may be a witness*.<br>Witness to the informed consent process<br><br>Name (please print) ______________________<br><br>Signature ______________________ Date ______________________<br>* Witness is not to be the Investigator, a member of the study team or their delegate. Witness must be 18 years or older.

Declaration by Study Doctor/Senior Researcher†

I have given a verbal explanation of the research project, its procedures and risks and I believe that the participant has understood that explanation.

Name of Study Doctor/<br>Senior Researcher† (please print) ______________________<br><br>Signature ______________________ Date ______________________

† A senior member of the research team must provide the explanation of, and information concerning, the research project.

Note: All parties signing the consent section must date their own signature.

Pittet LF, et al. BMJ Open 2023; 11:e052101. doi: 10.1136/bmjopen-2021-052101

BRACE_3mo survey_v4.1_10 02 2021

**Participant communication:**
**Email invitation wording:**<br><br>Hello [ec_fname],<br><br>Thankyou for your generous contribution to the BRACE trial. Your 3 month survey is now ready for you to complete.<br><br>You can access this survey by following the link below. It should take no longer than 10-15 minutes to complete depending on your answers.<br><br>Please answer these questions relating to the 3 month period since randomisation.<br><br>If you have any questions, please do not hesitate to contact us.<br><br>Thankyou for your time and ongoing support of the BRACE trial.<br><br>**The BRACE Trial Team**<br><br>**brace@mcri.edu.au**<br><br>You may open the survey in your web browser by clicking the link below:<br><br>[survey-url]<br><br>This link is unique to you and should not be forwarded to others.
**Survey Preamble:**<br><br>Thankyou for taking part in the BRACE trial<br><br>Your answers should reflect what happened in the last 3 months since you enrolled in the BRACE trial. We want to check with you, the data you have sent us via the app and make sure we haven't missed anything. We also have other questions for you to answer on your general health, exposure to COVID-19, tuberculosis, and cold sores recurrences.<br><br>The 3 month survey will take approximately 10-15 minutes to complete depending on your answers. You can save your responses and return to the survey at any time by clicking the link in the email.<br><br>Please answer these questions relating to the 3 month period since randomisation.<br><br>If you have any questions or problems relating to the survey, please do not hesitate to contact us.<br><br>Thankyou for your ongoing support of the BRACE trial.

BRACE_3mo survey_v4.1_10 02 2021

**Question**	**Options**

**Episodes of Illness including symptoms of fever, cough, shortness of breath and sore throat**	
Conditional logic<br>If a participants has reported no episodes of illness the following question appears:	
During the last 3 months you have NOT reported any episodes of illness which included any Fever, Cough, Sore throat and/or Shortness of breath symptoms.<br>Can you confirm that this is correct and that you have been without symptoms for the last 3 months?	1. Yes, I have been without the above symptoms for 3 months<br>0. No, I have had episode(s) of illness with the above symptoms to declare*<br><br>*please DO NOT include an episode of illness you are currently in - continue entering this information in your APP
Conditional logic<br>If a participants has reported episodes of illness, the following question appears showing them all their reported data, where x is the episode number.<br>x is between 1 and 4	
Over the last 3 months you have reported to us on x occasion(s) that you have had an episode of illness with one or more of the following symptoms: Fever, Cough, Sore throat and/or Shortness of breath.<br><br>Please check details below and confirm whether or not they are accurate.<br><br>Episode x:<br>Symptoms:<br>Tested for COVID:<br>Number of days too sick to work:<br>Number of days confined to bed:<br>Number of days hospitalised:<br><br>We understand that you may have had episodes of illness which consisted of other symptoms, however the episodes we would like you to report on here need to include symptoms of fever, cough, sore throat, and/or shortness of breath.<br><br>Can you confirm that 1) the above details are correct and that 2) you have been well outside these episodes?	1. Above details ARE correct and I have NOT had other episodes of illness which include symptoms of fever, cough, sore throat, and/or shortness of breath outside these episodes<br>2. Above details ARE correct but I HAVE HAD extra episode(s) of illness which involved fever, cough, sore throat or shortness of breath to declare<br>3. The above details ARE NOT correct (incorrect details or details missing)
Conditional logic<br>If a participant selects option 2 or 3 in the previous question, they receive the following:	
Please select all episodes requiring correction:	1. Episode 1

BRACE_3mo survey_v4.1_10 02 2021

Question	Options
	2. Episode 2<br>3. Episode 3<br>4. Episode 4

**CORRECTIONS to episodes of Illness including symptoms of fever, cough, shortness of breath and sore throat**	
The following series of questions will repeat for the number of episodes selected in the previous question	
Which aspects of Episode x require correction?	1. Date started<br>2. Date ended<br>3. Symptoms during episode<br>4. COVID-19 test date<br>5. COVID-19 test result<br>6. Days too sick to work<br>7. Days confined to bed<br>8. Days in hospital<br>9. This episode did not happen – please remove (add note below)
Date Episode x started	Date entry
Data Episode x ended	Date entry
**During this episode of illness, please select the symptoms you experienced:**	
Fever (> 38 degrees Celcius)	1. Yes
Intermittent cough	1. Yes
Persistent cough	1. Yes
Shortness of breath or difficulty breathing	1. Yes
Sore throat	1. Yes
Runny / blocked nose	1. Yes
Headache	1. Yes
Muscle and/or joint pain	1. Yes
Fatigue	1. Yes
Nausea, vomiting and/or diarrhoea	1. Yes
Loss of taste and/or smell	1. Yes
For how many days did you have an intermittent cough?	Integer
For how many days did you have a persistent cough?	Integer
For how many days did you have shortness of breath or difficulty breathing?	Integer
For how many days did you have a sore throat?	Integer
For how many days did you have a runny/blocked nose?	Integer
For how many days did you have a headache?	Integer
For how many days did you have muscle or joint pain?	Integer
For how many days did you have fatigue?	Integer
For how many days did you have vomiting/nausea/diarrhoea?	Integer
For how many days did you have a loss of taste or smell?	Integer

BRACE_3mo survey_v4.1_10 02 2021

Question	Options
COVID-19 test date for this episode	Date field
COVID-19 test result	1. Positive<br>2. Negative<br>3. Waiting on result
For how many consecutive days were you physically too unwell to work?	Integer
For how many consecutive days during this episode of illness were you confined to bed? (Meaning you found it very difficult to do any normal daily activities)	Integer
For how many days during this episode of illness did you stay overnight in hospital as a patient?	Integer
Do you have any other comments about this episode of illness?	Text field
**Missing end of episodes for episodes of Illness including symptoms of fever, cough, shortness of breath and sore throat**	
The following series of questions will repeat for the number of episodes in which the participant has not submitted an end of episode survey	
Fever (> 38 degrees Celcius)	1. Yes
Intermittent cough	1. Yes
Persistent cough	1. Yes
Shortness of breath or difficulty breathing	1. Yes
Sore throat	1. Yes
Runny / blocked nose	1. Yes
Headache	1. Yes
Muscle and/or joint pain	1. Yes
Fatigue	1. Yes
Nausea, vomiting and/or diarrhoea	1. Yes
Loss of taste and/or smell	1. Yes
For how many days did you have an intermittent cough?	Integer
For how many days did you have a persistent cough?	Integer
For how many days did you have shortness of breath or difficulty breathing?	Integer
For how many days did you have a sore throat?	Integer
For how many days did you have a runny/blocked nose?	Integer
For how many days did you have a headache?	Integer
For how many days did you have muscle or joint pain?	Integer
For how many days did you have fatigue?	Integer
For how many days did you have vomiting/nausea/diarrhoea?	Integer
For how many days did you have a loss of taste or smell?	Integer

BRACE_3mo survey_v4.1_10 02 2021

Question	Options
For how many consecutive days during this episode of illness were you too physically unwell to work?	Integer
How many days would you have normally worked during this episode of illness?	Integer
For how many of these workdays did you actually not go to work?	Integer
For how many consecutive days during this episode of illness were you confined to bed? (Meaning you found it very difficult to do any normal daily activities)	Integer
For how many days during this episode of illness did you stay overnight in hospital as a patient?	Integer
Which hospital(s)?	Text field
Were you tested for COVID-19 during this episode of illness?	0. No<br>1. Yes tested once<br>2. Yes tested twice<br>3. Yes tested 3 times
The following question series will repeat for each test indicated:	
Date of first test for COVID-19	Date field
What was the result of your first test for COVID-19?	1. Positive<br>2. Negative<br>3. Waiting on the result
Where were you tested?	1. At your workplace hospital<br>2. At another hospital<br>3. Through a GP<br>4. Through a BRACE swab<br>5. Other
Method of testing	2. Respiratory swab, traditional PCR testing (result therefore not available immediately)<br>6. Respiratory swab, with rapid testing (result in less than 1 hour)<br>1. Blood test<br>3. Finger prick blood test<br>5. Other<br>4. Unsure
If other, please specify:	
Did you have a POSITIVE test for any other virus? (Not including COVID-19)	1. Yes<br>0. No
Which was the other virus that tested positive? (e.g. influenza)	Text field
Have you seen your GP during this episode of illness?	1. Yes<br>0. No
Did you attend a hospital Emergency Department as a patient during this episode of illness?	1. Yes<br>0. No
Did you have pneumonia confirmed on x-ray?	1. Yes<br>0. No

BRACE_3mo survey_v4.1_10 02 2021

**Question**	**Options**
Did you receive oxygen?	1. Yes<br>0. No
How many days did you require oxygen for? (During this episode of illness)	Integer
Is there anything else you would like to tell us about any episode(s) of illness* you had in the last 3 months?<br><br>*illness which included any of Fever, Cough, Sore throat and/or Shortness of breath	Text field<br><br>*(We might need to contact you so that we can accurately enter data in the database)

Apart from the episodes of illness you just provided corrections to, have you had additional episodes of illness to declare? Please only include additional episodes of illness which include symptoms of fever, cough, shortness of breath or a sore throat	1. Yes<br>0. No
How many episodes of illness have you had which included any symptoms of fever, cough, sore throat or shortness of breath and lasted 3 or more days?	1. 1 episode<br>2. 2 episodes<br>3. 3 episodes<br>4. 4 episodes

The following series of questions will repeat for the number of episodes selected in the previous question	
**Additional Episode x**	
Episode x	
Episode x – start date: (when did your symptoms start?)	Date field
Episode x – end date: (when were you free of symptoms?)	Date field
**During this episode of illness, please select the symptoms you experienced:**	
Fever (> 38 degrees Celcius)	1. Yes
Intermittent cough	1. Yes
Persistent cough	1. Yes
Shortness of breath or difficulty breathing	1. Yes
Sore throat	1. Yes
Runny / blocked nose	1. Yes
Headache	1. Yes
Muscle and/or joint pain	1. Yes
Fatigue	1. Yes
Nausea, vomiting and/or diarrhoea	1. Yes
Loss of taste and/or smell	1. Yes
For how many days did you have an intermittent cough?	Integer
For how many days did you have a persistent cough?	Integer
For how many days did you have shortness of breath or difficulty breathing?	Integer
For how many days did you have a sore throat?	Integer

BRACE_3mo survey_v4.1_10 02 2021

Question	Options
For how many days did you have a runny/blocked nose?	Integer
For how many days did you have a headache?	Integer
For how many days did you have muscle or joint pain?	Integer
For how many days did you have fatigue?	Integer
For how many days did you have vomiting/nausea/diarrhoea?	Integer
For how many days did you have a loss of taste or smell?	Integer
For how many consecutive days during this episode of illness were you too physically unwell to work?	Integer
How many days would you have normally worked during this episode of illness?	Integer
For how many of these workdays did you actually not go to work?	Integer
For how many consecutive days during this episode of illness were you confined to bed? (Meaning you found it very difficult to do any normal daily activities)	Integer
For how many days during this episode of illness did you stay overnight in hospital as a patient?	Integer
Which hospital(s)?	Text field
Were you tested for COVID-19 during this episode of illness?	0. No<br>1. Yes tested once<br>2. Yes tested twice<br>3. Yes tested 3 times
The following question series will repeat for each test indicated:	
Date of first test for COVID-19	Date field
What was the result of your first test for COVID-19?	1. Positive<br>2. Negative<br>3. Waiting on the result
Where were you tested?	1. At your workplace hospital<br>2. At another hospital<br>3. Through a GP<br>4. Through a BRACE swab<br>5. Other
Method of testing	2. Respiratory swab, traditional PCR testing (result therefore not available immediately)<br>6. Respiratory swab, with rapid testing (result in less than 1 hour)<br>1. Blood test<br>3. Finger prick blood test<br>5. Other<br>4. Unsure
Did you have a POSITIVE test for any other virus? (Not including COVID-19)	1. Yes<br>0. No

BRACE_3mo survey_v4.1_10 02 2021

Question	Options
Which was the other virus that tested positive? (e.g. influenza)	Text field
Have you seen your GP during this episode of illness?	1. Yes<br>0. No
Did you attend a hospital Emergency Department as a patient during this episode of illness?	1. Yes<br>0. No
Did you have pneumonia confirmed on x-ray?	1. Yes<br>0. No
Did you receive oxygen?	1. Yes<br>0. No
How many days did you require oxygen for? (During this episode of illness)	Integer
Is there anything else you would like to tell us about any episode(s) of illness* you had in the last 3 months?<br><br>*illness which included any of Fever, Cough, Sore throat and/or Shortness of breath	Text field<br><br>*(We might need to contact you so that we can accurately enter data in the database)
**Other COVID-19 tests**	
The following series of questions will repeat for the number of covid tests during other symptoms the participant has reported (up to 4 covid tests)	
[On date, you reported having a COVID-19 test with these symptoms: prior symptom(s)]<br><br>Was this more than just a single day of illness?	1. Yes, more than one day<br>0. No, just a single day
If single day of symptoms: [previous question=no]	
What was the result of your COVID-19 test?	1. Positive<br>2. Negative<br>3. Waiting on the result
If more than a single day of illness [previous questions=yes]	
Start date of episode of illness with no fever, cough, sore throat or shortness of breath during which you had a COVID-19 test:	Date field
End date of this episode of illness: (when were you free of symptoms?)	Date field
**During this episode of illness please select the symptoms you experienced:**	
Fever (> 38 degrees Celcius)	1. Yes
Intermittent cough	1. Yes
Persistent cough	1. Yes
Shortness of breath or difficulty breathing	1. Yes
Sore throat	1. Yes
Runny / blocked nose	1. Yes
Headache	1. Yes

BRACE_3mo survey_v4.1_10 02 2021

Question	Options
Muscle and/or joint ache	1. Yes
Fatigue	1. Yes
Nausea, vomiting and/or diarrhoea	1. Yes
Loss of taste and/or smell	1. Yes
For how many days did you have a fever?	Integer
For how many days did you have an intermittent cough?	Integer
For how many days did you have a persistent cough?	Integer
For how many days did you have shortness of breath or difficulty breathing?	Integer
For how many days did you have a sore throat?	Integer
For how many days did you have a runny/blocked nose?	Integer
For how many days did you have a headache?	Integer
For how many days did you have muscle or joint ache?	Integer
For how many days did you have fatigue?	Integer
For how many days did you have vomiting / nausea / diarrhoea?	Integer
For how many days did you have a loss of taste or smell?	Integer
COVID-19 test date for this episode:	Date field
COVID-19 test result	1. Positive<br>2. Negative<br>3. Waiting on the result
For how many consecutive days were you physically too unwell to work?	Integer
For how many consecutive days during this episode of illness were you confined to bed? (Meaning you found it very difficult to do any normal daily activities)	Integer
For how many days during this episode of illness did you stay overnight in hospital as a patient?	Integer
Have you seen your GP during this episode of illness?	1. Yes<br>0. No
Did you have pneumonia confirmed on x-ray?	1. Yes<br>0. No
Did you receive oxygen?	1. Yes<br>0. No
How many days did you require oxygen for? (During this episode of illness)	Integer

**Additional COVID-19 tests**	
Apart from any COVID-19 tests already mentioned as part of episodes of illness were you tested for COVID-19 at any other time during the last 3 months?	0. No<br>1. Yes one other time<br>2. Yes two other times<br>3. Yes three other times

BRACE_3mo survey_v4.1_10 02 2021

**Question**	**Options**
**First additional COVID-19 test**<br>This will repeat for each additional COVID-19 test	
Date of the additional COVID-19 test:	Date field
Result of the COVID-19 test:	1. Positive<br>2. Negative<br>3. Waiting on the result
Was this test done when you were asymptomatic?	1. Yes<br>0. No
What symptoms were you experiencing when you had this test?	Text field

**Confirmation of any hospitalisations**	
We just wanted to double check with you whether you've been hospitalised in the last 3 months?	0. No, I have not been hospitalised in the last 3 months<br>1. Yes, I was hospitalised
How many times were you hospitalised?	1. Once<br>2. Twice<br>3. Three times
The following series of questions repeat for each reported hospitalisation	
Date you were first admitted to hospital:	Date field
Date you were discharged from hospital after your first admission:	Date field
Did you receive oxygen during your first admission?	1. Yes<br>0. No
Number of days you received oxygen:	Integer
Were you admitted to the critical / intensive care unit (ICU) during this hospitalisation?	1. Yes<br>0. No
Date you were admitted to ICU:	Date field
Date you were discharged from ICU:	Date field
Were you assisted to breath through the use of mechanical ventilation?	1. Yes<br>0. No
Number of days you were assisted to breathe by mechanical ventilation:	Integer
What was the reason for your first hospitalisation?	1. COVID-19 related<br>2. Other infection, not COVID-19 related<br>3. Trauma, accident<br>4. Elective surgery<br>5. Pregnancy related<br>6. Related to an underlying chronic disease<br>7. Other cause, not infectious
Please give us more detail on the reason for this hospitalisation:	Text field
Which hospital were you admitted to:	Text field
Just to summarise, overall in the last 3 months, have you been absent from work for any reason?<br>For example, this includes absence due to illness, vaccine reaction, holiday, quarantine.<br>Note that working from home is not considered as being absent from work.	1, Yes<br>0, No

BRACE_3mo survey_v4.1_10 02 2021

**Question**	**Options**
Please tell us how many days were you absent from work for each of the following reasons:	Header
Issue with vaccination site	integer
Mandatory quarantine while mildly ill and/or waiting for COVID-19 test result	integer
Too ill to go to work (but not hospitalised)	integer
Hospitalisation	integer
Mandatory quarantine while not ill (e.g. following travel or contact with COVID-19 case)	integer
Annual leave, holidays, planned absence	integer
Carer leave	integer
Pregnancy-related leave or hospitalisation	integer
Absence for any other reason(s)	integer
Number of days absent (total)	calculated field
We have calculated you have been absent for a total of <u>[m3_work_total_calc]</u>.<br>Could you please confirm this is accurate?	1, Yes<br>0, No (please adjust the days reported above)
Please detail here the other reason(s) and the number of days of absence for each of the other reason(s)	notes field

**COVID-19 Exposure**	
You previously answered working in the [answer from baseline survey]. Have you changed workplace in the last 3 months?	1. Yes<br>0. No
You previously answered working in the [free text answer from baseline survey]. Have you changed workplace in the last 3 months?	1. Yes<br>0. No
If yes, what department best describes your workplace now?	1. Emergency Department<br>2. Intensive Care Unit / High Dependency Unit<br>3. Operating Theatre<br>4. General ward<br>5. Pharmacy<br>6. Other ward/area<br>7. Paramedic / Ambulance<br>8. Aged care facility<br>9. Practice outside of hospital
If other, please specify:	Text field
On an average week, in last 3 months, how many hours are you in direct contact with patients?	0. No direct patient contact<br>1. < 10 hours<br>2. 10 - 20 hours<br>3. >20 hours
Have there been confirmed COVID-19 patients in your department?	0. No (not that I'm aware of)<br>1. Yes, there has been at least one confirmed case of COVID-19
Have you spent 15 minutes or more in direct contact with a confirmed COVID-19 patient?	0. No (not that I'm aware of)<br>1. Yes, but I was always wearing PPE (Personal Protective Equipment)<br>2. Yes, and I was not always wearing PPE (Personal Protective Equipment)

BRACE_3mo survey_v4.1_10 02 2021

**Question**	**Options**
Have any of the people living in your household been confirmed as having a COVID-19 infection?	1. Yes<br>0. No
Have you been exposed to a confirmed COVID-19 case outside your workplace or household?	1. Yes<br>0. No
**Medication**	
When you enrolled in the BRACE study, you reported taking [answer from baseline survey].<br><br>Did you take any of these medications for more than 30 days in a row, in the last 3 months?	1. lopinavir-ritonavir (e.g. Kaletra)<br>2. Hydroxychloroquine<br>3. Azithromycin<br>4. Oseltamivir (eg. Tamiflu)<br>5. Antihypertensive medication (to reduce blood pressure)<br>6. None of the above
If possible, could you add the name of the antihypertensive medication that you take:	Text field
**Vaccinations**	
Have you received a COVID-19 specific vaccine in the last 3 months (e.g. Moderna AG, Pfizer, AstraZeneca)?	1. Yes<br>0. No
Which COVID-19 vaccine did you receive?	1, Astra Zenica/Oxford (ChAdOx1, Covishield)<br>2, Pfizer/BioNTech (BNT162b2, Comirnaty)<br>3, Moderna (mRNA-1273)<br>5, Sinovac (CoronaVac)<br>6, Novavax (NVX-CoV2373)<br>7, Johnson & Johnson (Ad26.COV2.S)<br>8, Gam-Covid-Vac (Sputnik V)<br><< Insert other COVID-19 vaccine>>
If other, please specify:	Text field
For each vaccine selected the following questions are asked:	
How many doses of [COVID-19-specific vaccine name] have you received in the last 3 months?	1. One<br>2. Two<br><< Insert number of doses>>
For each dose selected the following questions are asked:	
When did you receive the first dose of [COVID-19-specific vaccine name]?	
When did you receive the second dose of [COVID-19-specific vaccine name]?	
Did you receive any other vaccines in the last 3 months (apart from those received in the context of the BRACE trial)?	1. Yes<br>0. No
If yes, which vaccine(s) did you receive?	1. Diphtheria-tetanus vaccine (ADT Booster)<br>2. Diphtheria-tetanus-pertussis vaccine (Boostrix, Adacel, Tripacel)<br>3. Diphtheria-tetanus-pertussis-polio vaccine (Boostrix-IPV, Adacel Polio, Quadracel)<br>4. Polio vaccine (IPOL)<br>5. Hepatitis B vaccine (Engerix-B, H-B-Vax II)<br>6. Hepatitis A vaccine (Havrix, Avaxim, Vaqta)<br>7. Hepatitis A-hepatitis B vaccine (Twinrix)<br>8. Hepatitis A-typhoid vaccine (Vivaxim)

BRACE_3mo survey_v4.1_10 02 2021

Question	Options
	9. Typhoid injected vaccine (Typhim Vi)<br>10. Typhoid oral vaccine (Vivotif Oral)<br>11. Influenza vaccine (Afluria, Fluad Quad, Fluarix, FluQuadri, Influvac, Vaxigrip, Vaxigroup)<br>12. Papillomavirus vaccine (Cervarix, Gardasil)<br>13. Meningococcal vaccine (Menveo, Menactra, MenQuadfi, NeisVac, Bexsero, Trumenba)<br>14. Pneumococcal vaccine (Prevenar, Synflorix, Pneumosil, Pneumovax)<br>15. Japanese encephalitis vaccine (Imojev, JEspect)<br>16. Rabies vaccine (Rabipur)<br>17. Yellow fever vaccine (Stamaril)<br>18. Measles-mumps-rubella (Priorix, M-M-R II, ProQuad)<br>19. Measles-mumps-rubella-varicella (Priorix-tetra, ProQuad)<br>20. Varicella vaccine (Varilrix, Varivax)<br>21. Zoster live vaccine (Zostavaq)<br>22. Zoster non-live vaccine (Shingrix)<br>23. Tuberculosis vaccine (BCG) outside the context of the trial<br>24. Other
How many other vaccine(s) did you receive?	1. 1<br>2. 2<br>3. 3
Other vaccine 1/2/3 - please describe:	Text field
When did you receive the [as above] vaccine?<br>This question is asked for each vaccine checked in the previous question	Date field
Which meningococcal vaccine did you receive?	1. Menveo, Menactra, MenQuadfi<br>2. NeisVac<br>3. Bexsero<br>4. Trumbena
Which pneumococcal vaccine did you receive?	1. Conjugated vaccine (Prevenar, Synflorix, Pneumosil)<br>2. Non-conjugated vaccine (Pneumovax)
**Other clinical trial**	
Since recruitment in the BRACE trial on [ra_rand_datetime] have you been included in another COVID-19 clinical trial?	1. Yes<br>0. No
If yes to above:	
Which other clinical trial are you in?	Text field
What vaccine or intervention did you receive in the context of the other trial?	Text field
When did you enter the other trial?	Date field
**Tuberculosis Exposure**	
Have you stayed in a high tuberculosis burden country in the last 3 months?	1. Yes<br>0. No

BRACE_3mo survey_v4.1_10 02 2021

**Question**	**Options**
(Including: India, China, Indonesia, the Philippines, Pakistan, Nigeria, Bangladesh and South Africa.)	References: https://www.who.int/news-room/fact-sheets/detail/tuberculosis, http://www.stoptb.org/countries/tbdata.asp
Have you had a tuberculin skin test in the last 3 months?	0. No<br>1. Yes, it was negative, < 5mm<br>2. Yes, the reading was 5-10mm<br>3. Yes, the reading was 10-15mm<br>4. Yes, the reading was >15mm
Have you been exposed to a suspected or confirmed case of tuberculosis in the last 3 months?	0. No (not that I'm aware of)<br>1. Yes, I've been exposed to a suspected case<br>2. Yes, I've been exposed to a confirmed case
Have you been diagnosed with latent or active tuberculosis in the last 3 months?	0. No<br>1. Yes, I've been diagnosed with latent tuberculosis<br>2. Yes, I've been diagnosed with active tuberculosis
What treatment did you receive to treat latent or active tuberculosis?	Text field
**Vaccine site reaction**	
Conditional logic<br>Participant receive a different question depending if they completed their Vaccine Diary or not	
You completed your vaccine diary for the 2 weeks following vaccination.<br><br>Did you experience any of the following (pain, redness, swelling, tenderness) BEYOND this 2 week period?	0. No<br>1. Yes, I experienced pain<br>2. Yes, I have noticed redness at the vaccination site<br>3. Yes, I have noticed swelling at the vaccination site<br>4. Yes, I have noticed tenderness at the vaccination site
You did not complete your vaccine diary following vaccination.<br><br>Did you experience any of the following (pain, redness, swelling, tenderness) after vaccination?	0. No<br>1. Yes, I experienced pain<br>2. Yes, I have noticed redness at the vaccination site<br>3. Yes, I have noticed swelling at the vaccination site<br>4. Yes, I have noticed tenderness at the vaccination site
Conditional logic<br>Participant receives the following questions depending on their answers above	
How many days after vaccination did the pain start? At day number:	Integer<br>NB: Day number 1 is the day you received your vaccination
For how many days did the pain last?	Integer<br>Days
How many days after vaccination did the redness start? At day number:	Integer<br>NB: Day number 1 is the day you received your vaccination

BRACE_3mo survey_v4.1_10 02 2021

Question	Options
For how many days did the redness last?	Integer<br>Days
What was the largest diameter of the redness, at its worst (in cm)?	Number<br>Please provide answer in cm
How many days after vaccination did the swelling start? At day number:	Integer<br>NB: Day number 1 is the day you received your vaccination
For how many days did the swelling last?	Integer<br>Days
What was the largest diameter of the swelling at its worst (in cm)?	Number<br>Please provide answer in cm
How many days after vaccination did the tenderness start? At day number:	Integer<br>NB: Day number 1 is the day you received your vaccination
For how many days did the tenderness last?	Integer
Did this significantly interfere with your daily activities?	1. It did not significantly interfere with my daily activities<br>2. It somewhat interfered with my daily activities<br>3. It prevented me from doing my daily activities
Please describe how it interfered, and for how long:	Text field
Regarding your vaccination site, did you have to use medication or consult a medical doctor?	1. I did not need to take any medication, nor see a medical doctor<br>2. I had to consult a medical doctor or be hospitalised<br>3. I had to use pain medication
Did you see a medical doctor from the BRACE team, or was it external to the study?	1. BRACE team doctor only<br>2. External doctor only<br>3. Both BRACE team and external doctor
Please describe when you saw the doctor, and what was discussed:	Text field
Which medication did you take?	Text field
For how many days did you use this medication?	Integer<br>Days
Regarding the level of tenderness only:<br><br>How would you describe level of discomfort at its worst, in the past 3 months?	1. Mild discomfort to touch<br>2. Discomfort with movement<br>3. Significant discomfort at rest
Please describe when the discomfort occurred, and for how long:	Text field
Have you noticed or felt swollen glands close to the vaccination site?<br><br>(Usually felt under the armpit or in the neck on the vaccination side of the body)	0. No<br>1. Yes<br>2. Unsure
Where have you noticed or felt a swollen gland?	1. Under the armpit<br>2. In the neck

BRACE_3mo survey_v4.1_10 02 2021

Question	Options
	3. Other
If other, please tell us where:	Text field
How big was the swollen gland (in cm) under the armpit?	Number
How big was the swollen gland (in cm) in the neck?	Number
How big was the swollen gland (in cm) in another location?	Number
Has there been pus coming out of the swollen gland under the armpit?	1. Yes<br>0. No
Has there been pus coming out of the swollen gland in the neck?	1. Yes<br>0. No
Has there been pus coming out of the swollen gland in another location?	1. Yes<br>0. No
<u>Message to participants</u><br>We would love to see a picture of your vaccination site, even if it is not visible anymore.<br><br>How to take the best picture:<br>- Attach a standard-sized object to your upper arm (e.g. coin or measuring tape or ruler) using rolled up sticky tape or BluTack, adjacent to the vaccination site.<br>- Hold your phone approx. 15cm away from the area being photographed.<br>- Ensure the entire injection site and coin are in the photo and in focus.	
Please upload your photo here.	Option to upload a file
Which of the following best describes the vaccination site today?	1. No mark<br>2. Skin colour mark without redness, normal scar formation<br>3. Red mark<br>4. Red mark with discharge<br>5. Red mark with crusting<br>6. Ulcer (open sore)<br>7. Vaccination site still looks 'angry' with swelling and/or redness all around it<br>8. Keloid scar formation (meaning an abnormal thick scar)<br>9. Other
If other, please describe:	Text field
Have you experienced any of the following symptoms at your vaccination site in the last 3 months?	8. No scar or normal scarring<br>1. Ulcer (open sore) 2. Large ulcer or sore (>1.5 cm in diameter)<br>3. Persistent discharge >2 weeks<br>4. Swelling around the vaccination site<br>5. Redness around the vaccination site<br>6. Keloid scar formation (meaning an abnormal thick scar)

BRACE_3mo survey_v4.1_10 02 2021

**Question**	**Options**
	7. Other
If other, please describe:	Text field
**HSV questions**	
Have you had a cold sore episode since you enrolled in the BRACE trial on [date of randomisation]?<br><br>These are small painful blisters on the lips or around the mouth, also known as fever blisters or herpetic infection	1. Yes<br>0. No
How many cold sore episodes did you have in the last 3 months?	Integer<br>Please answer to the best of your memory.
When did the first episode start? Please only consider the last 3 months	Date field<br>Please approximate the date to the best of your memory.
Have you noticed any change in your cold sores recurrence in the last 3 months, in terms of:<br><br>- Frequency (how often you get cold sores)<br>- Duration (how long a cold sore episode lasts)<br>- Severity (how painful, disabling, extensive the lesions are)<br>- Impact on quality of life (social, aesthetic, work, etc.)	1. Yes<br>0. No
**To what extent has it changed?**	
Frequency<br>(how often you get cold sores)	1. The episodes were less frequent<br>2. The episodes were more frequent<br>3. The episodes occur at the same frequency
Duration<br>(how long a cold sore episode lasts)	1. The episodes were shorter<br>2. The episodes were longer<br>3. The episodes had the same duration
Severity<br>(how painful, disabling, extensive the lesions are)	1. The episodes were less severe<br>2. The episodes were more severe<br>3. The episodes had the same severity
Impact on quality of life<br>(social, aesthetic, work, etc.)	1. The episodes had less impact on my quality of life<br>2. The episodes had more impact on my quality of life<br>3. The impact on my quality of life did not change
You previously said that you have taken prophylactic (preventive) treatment [answer from baseline survey], to prevent cold sore recurrences.<br><br>Were you taking [answer from baseline survey] on the day of randomisation ([date of randomisation])?	1. Yes<br>0. No
Are you still taking [answer from baseline survey] today?	1. Yes<br>0. No

BRACE_3mo survey_v4.1_10 02 2021

**Question**	**Options**
You previously said that you have taken prophylactic (preventive) treatment [answer from baseline survey], to prevent cold sore recurrences.<br><br>Were you taking [answer from baseline survey] on the day of randomisation [date]?	1. Yes<br>0. No
Have you received any treatment for cold sores in the last 3 months?	1. Yes<br>0. No
If yes, why?	1. To treat an active cold sore<br>2. To prevent further cold sores (treatment typically lasting more than 1 month)<br>3. Both to treat and to prevent cold sores
Which preventive treatment have you received in the last 3 months?<br>(Tick all that apply).	1. Aciclovir (also called Zovirax, Acyclo-V, Lovir)<br>2. Valaciclovir (also called Valtrex, Valacor, Zelitrex, Shilova)<br>3. Famciclovir (also called Famir, Favic, Famlo, Ezovir)<br>4. Lysine<br>5. Other, please specify<br>6. Don't know
If other, please specify:	Text field
For each treatment ticked, the following series of questions are asked	
For how long were you taking Aciclovir? Please only consider the last 3 months<br><br>Please answer in months. If less than a month, please answer in fraction of month (1 week = 0.24 months).	Number<br>In months, or fraction of months
Are you still taking Aciclovir treatment today?	1. Yes<br>0. No
Thank you so much for your participation in the BRACE trial!<br>Please do not hesitate to contact us via e-mail or phone if you have any concerns.<br>For Victoria: brace@mcri.edu.au<br>For Western Australia: brace@telethonkids.org.au<br>For South Australia: BRACE.trial@sahmri.com<br>For Netherlands: BRACE@umcutrecht.nl<br>For Spain: BRACE@umcutrecht.nl<br>For UK: bracetrial@exeter.ac.uk	
**Medicare number** (If participant did not provide Medicare number at enrolment)	
Please provide your Medicare card number:	Text field
