# Supplementary material for: BCG vaccination to reduce the impact of COVID-19 in healthcare workers: Protocol for a randomised controlled trial (BRACE trial)
Source: BMJ Open. 2021 Oct 27;11(10):e052101. doi: 10.1136/bmjopen-2021-052101 (PMC8557250; doi:10.1136/bmjopen-2021-052101)
Supplement: Supplementary data [file bmjopen-2021-052101supp002.pdf]

Supplementary Table 1: Sample size calculation for the primary outcomes at the final analysis, the interim analysis, and the pre-planned meta-analysis of the two stages of the severe COVID-19 outcome

	Estimation of occurrence		Risk ratio	Alpha (2-sided p-value)		Power	Total number of participants required	% loss to follow up	Total number of participants required (adjusted by drop out)
	Control group	BCG group		Allowed	% global				
**Primary outcomes**									
1st co-primary outcome: symptomatic COVID-19	55%	45%	0.82	0.005	10%	95%	2016	-	-
2nd co-primary outcome: severe COVID-19	4%	2.7%	0.67	0.04	80%	80%	6076	16%	7244
**Interim analysis**									
Interim analysis stopping rule: incidence of severe COVID-19	66 cases of severe COVID-19	33 cases of severe COVID-19	0.5	0.005	10%	72%	100 cases of severe COVID-19	-	-
**Meta-analysis**									
Meta-analysis; incidence of severe COVID-19	4%	2.7%	0.67			90%	8062	20%	10078
